# p38β-mediated BiP phosphorylation drives stemness and chemoresistance by suppressing UPR activation in hepatocellular carcinoma

**DOI:** 10.1038/s41467-026-76073-7

**Published:** 2026-08-05

**Authors:** Liang Xu, Ianto Bosheng Huang, Minghe Zhang, Yunong Xie, Bing Li, Linglin Liu, Long Hin Ng, Yimiao He, Yan Liu, Tin-Lok Wong, Xiaoyun Lu, Terence Kin-Wah Lee, Jing-Ping Yun, Lei Jin, Stephanie Ma, Man Tong

**Affiliations:** 1 School of Biomedical Sciences, The Chinese University of Hong Kong, Hong Kong, China; 2 School of Biomedical Sciences, Li Ka Shing Faculty of Medicine, The University of Hong Kong, Hong Kong, China; 3 Laboratory for Synthetic Chemistry and Chemical Biology Limited, Health@innoHK, Hong Kong, China; 4 State Key Laboratory of Metabolic Dysregulation and Prevention and Treatment of Esophageal Cancer, Tianjian Laboratory of Advanced Biomedical Sciences, School of Convergence Medicine, Zhengzhou University, Zhengzhou, China; 5 International Cooperative Laboratory of Traditional Chinese Medicine Modernization and Innovative Drug Discovery of Chinese Ministry of Education (MOE), Guangzhou City Key Laboratory of Precision Chemical Drug Development, Guangzhou City Key Laboratory of Precision Chemical Drug Development, School of Pharmacy, Jinan University, Guangzhou, China; 6 Department of Applied Biology and Chemical Technology, The Hong Kong Polytechnic University, Hong Kong, China; 7 Department of Pathology, Sun Yat-Sen University Cancer Centre, Guangzhou, China; 8 State Key Laboratory of Liver Research, The University of Hong Kong, Hong Kong, China; 9 Department of Clinical Oncology, Shenzhen Key Laboratory for Cancer Metastasis and Personalized Therapy, The University of Hong Kong – Shenzhen Hospital, Shenzhen, China

**Keywords:** Cancer stem cells, Oncogenes

## Abstract

Tumor-initiating cells (TICs) promote tumor initiation and therapy resistance, yet the kinase regulators that sustain TICs remain incompletely defined. Here, we identify the stress kinase p38β (MAPK11) supports TIC maintenance and drug resistance in hepatocellular carcinoma (HCC). Integrated analysis of chemotherapy-enriched HCC spheroids, and DepMap data prioritized p38β as a kinase linked to stemness and chemoresistance. High p38β expression correlates with poor prognosis and aggressive clinicopathological features in HCC patients. Mechanistically, p38β phosphorylates the endoplasmic reticulum (ER) chaperone BiP at threonine 648, enhancing its association with the unfolded protein response (UPR) sensors PERK and IRE1-α. This modification suppresses UPR activation and reduces unfolded protein accumulation, thereby preserving ER proteostasis under chemotherapeutic stress. Functionally, p38β-driven BiP phosphorylation sustains TIC phenotypes and cisplatin resistance in vitro and in vivo. BiP inhibition with HA15 restores UPR signaling and sensitizes patient-derived xenograft and organoid models to cisplatin, revealing a targetable p38β–BiP axis in HCC.

## Abstract (web summary)

Tumor-initiating cells drive chemoresistance in hepatocellular carcinoma (HCC). Here, the authors discover that p38β/MAPK11 phosphorylation of BiP promotes stemness and chemoresistance in HCC.

## Introduction

Drug resistance remains a central obstacle in the treatment of hepatocellular carcinoma (HCC), where most patients experience tumor progression and relapse despite therapies including chemotherapy, radiotherapy or targeted therapy^1,2^. Mounting evidence suggests that cytotoxic treatments fail to eradicate tumor-initiating cells (TICs), a subpopulation with tumor-initiating and stem-like properties, capable of withstanding therapy and driving recurrence. The molecular circuitry that enables TICs to endure therapeutic stress, particularly via kinase-driven signaling pathways, remains poorly defined. Emerging evidence points to cellular stress adaptive responses, such as the endoplasmic reticulum (ER) stress pathway, as critical for TIC survival under therapeutic pressure^3–5^, yet how these programs interface with oncogenic kinase signaling to sustain stem-like states and resistance remains unclear.

As one of the most extensively characterized cellular stress-adaptive programs, the ER stress response enables cancer cells to survive under hostile microenvironmental conditions, including hypoxia, nutrient deprivation, and oxidative stress^6^. Persistent ER stress activates the unfolded protein response (UPR), a tripartite signaling network centered on the ER transmembrane sensors PERK, IRE1α, and ATF6, which collectively coordinate translational attenuation, transcriptional reprogramming, and proteostasis restoration^7–10^. Under homeostatic conditions, all three sensors are maintained in an inactive state through binding to the chaperone BiP/GRP78, positioning BiP as the master gatekeeper of UPR activation^7,9,11^. Upon stress-induced dissociation of BiP, PERK suppresses global protein synthesis via eIF2α phosphorylation while selectively inducing ATF4-dependent adaptive programs^12^; IRE1α initiates unconventional splicing of XBP1 to reinforce ER folding and lipid biosynthesis capacity^13,14^; and ATF6 translocates to the Golgi to activate chaperone and ER-associated degradation pathways^15^.

Importantly, UPR signaling displays a context-dependent duality in cancer. Sustained or excessive activation of PERK-CHOP or IRE1-JNK pathways can trigger apoptosis^16^. In contrast, a finely tuned, adaptive UPR supports tumor cell survival by enforcing a low-protein-synthesis state, enhancing metabolic plasticity, and conferring resistance to cytotoxic stress^6,16,17^. Recent studies further implicate UPR signaling in the maintenance of TICs by enhancing redox homeostasis, and rewiring transcriptional programs linked to stemness, plasticity, and immune evasion^16,18,19^. These observations underscore the role of UPR, in particular its BiP-centered regulatory node, as a critical stress-buffering system that may cooperate with oncogenic kinase signaling to sustain TIC phenotypes under therapeutic pressure.

The p38 mitogen-activated protein kinase (MAPK) family, best known for mediating cellular stress responses, has also been implicated in cancer progression and therapeutic resistance^20,21^. It comprises four isoforms: p38α, p38β (MAPK11), p38γ, and p38δ^22^. While p38α has been extensively characterized in oncogenesis^21,23^, the biological role of p38β, a distinct isoform capable of autoactivation via cis autophosphorylation at T180 within its activation loop, spontaneously in vitro and in a regulated manner in cells^24,25^, remains poorly understood. Whether p38β contributes to TIC maintenance, drug resistance, or adaptation to ER stress remains mostly unknown. Given that kinase signaling plays a central role in coordinating the UPR^9,26^, it is plausible that p38β functions as a molecular integrator linking cytotoxic stress to TIC persistence.

In this study, we integrated kinome-focused transcriptomic profiling of chemotherapy-enriched, TIC-like HCC spheroids with DepMap (DEMETER2) dependency data to prioritize kinases linked to stemness and chemoresistance. Through integrative analysis, we identified p38β as a top candidate required for both tumor-initiating capacity and drug resistance. Mechanistically, we reveal that p38β promotes BiP phosphorylation, attenuates UPR activation, and preserves proteostasis under stress, thereby sustaining cancer stemness and chemoresistance. These findings uncover a previously unappreciated p38β-BiP axis as a critical regulator of ER homeostasis in TICs and a potential therapeutic target in refractory HCC.

## Results

### High p38β expression correlates with poor prognosis and chemotherapy resistance in HCC

**Fig. 1:**
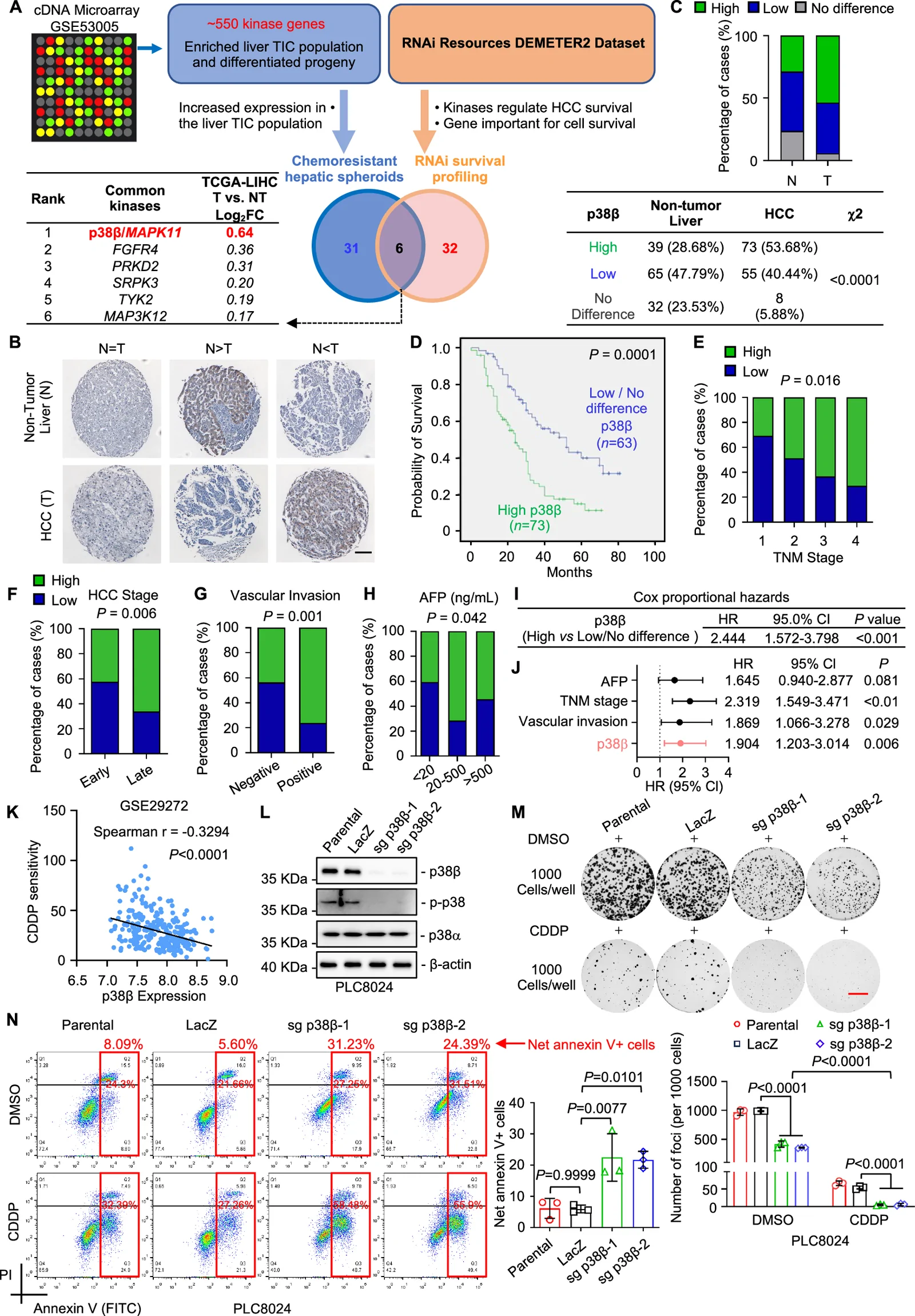
Identification of p38β as a critical kinase supporting chemoresistance in hepatic TICs. **A** Integration of the published HCC spheroid microarray dataset (GEO: GSE53005) with the DepMap DEMETER2 RNAi dataset identified six overlapping kinases associated with spheroid chemoresistance and cancer cell survival, with p38β/MAPK11 as the top candidate. **B**, **C** IHC staining (**B**) and quantification of p38β expression (**C**) in paired HCC (T) and adjacent non-tumor liver (N) tissues (*n* = 136 pairs). Three representative pairs illustrate *N* = T, *N* > T, and *N* < T staining patterns. Scale bar, 100 μm. **D** Kaplan-Meier overall survival analysis stratified by p38β expression; two-sided log-rank test (no multiple comparison adjustment). **E–H** Associations between p38β expression and TNM stage (**E**), HCC stage (**F**), vascular invasion (**G**) and AFP (**H**) in 136 HCC cases. **I** Univariate two-sided Cox proportional hazards analysis of overall survival stratified by p38β expression; hazard ratio (HR) and 95% confidence interval (CI) are shown without multiple comparison adjustment. **J** Multivariable Cox regression analysis including AFP, TNM stage, vascular invasion, and p38β. HRs with 95% CIs are shown. **K** p38β expression shows a moderate negative correlation with CDDP sensitivity in the NCI-60 panel (*n* = 60 cancer cell lines), using a two-sided Spearman’s correlation test with CellMiner data. **L** Western blot analysis validating CRISPR-Cas9 sgRNA-mediated knockout of p38β and assessing phospho-p38 and p38α levels in PLC8024 HCC cells. The samples derive from the same experiment, but separate gels for p38β, p-p38, p38α and *β*-actin were processed in parallel. **M** Foci formation assays in the indicated PLC8024 cells treated with DMSO or CDDP (10 μM). Bottom, quantification of foci numbers. Scale bar, 1 cm; *n* = 3 independent experiments. **N** Annexin V flow-cytometric analysis of p38β-deficient PLC8024 cells after CDDP treatment (10 μM, 24 h; *n* = 3 independent experiments). Data represent three independent experiments (**L**). Values are mean ± SD (**M**, **N**). Chi-square test (**C**, **E**–**H**). One-way ANOVA followed by Tukey’s multiple-comparisons test (**M**, **N**).

To investigate kinase-dependent mechanisms that sustain chemoresistance in liver cancer, we leveraged our previously published transcriptomic profiling of a chemoresistant hepatic tumor sphere (TIC-enriched) model (GSE53005)^27^. Briefly, tumor-initiating cells were enriched from the HCC cell line PLC/PRF/5 by serial passages in serum-free spheroid culture supplemented with doxorubicin (2 mg/mL) and cisplatin (1 mg/mL), selecting for a self-renewing, chemoresistant population. The chemoresistant tumor spheres and their differentiated counterparts were subjected to cDNA microarray profiling. We acknowledge that this transcriptomic profiling of chemoresistant hepatic tumor spheres was performed at drug doses 300–10,000 times higher than those typically used in experimental and clinical settings. This high-drug selection model was used here as a candidate-generating resource rather than as a direct short-term pharmacological response model. Using this dataset, we identified 37 differentially expressed kinase genes (log2 fold-change > 0.5) between chemoresistant hepatic tumor spheres and their differentiated counterparts. To prioritize kinases that are important for HCC cell survival, genetic dependency scores of HCC cell lines obtained from the DepMap DEMETER2 RNAi dataset (https://depmap.org/R2-D2/) were compared. Kinase genes were ranked by their average dependency across HCC lines (0 indicates no dependency, and more negative values indicate stronger dependency), and we applied a heuristic cutoff (gene effect ≤ −0.3) to define a candidate set, yielding 38 kinase genes. These two gene lists were integrated and revealed six overlapping kinases required for both chemoresistance and cell survival, with p38β (MAPK11) emerging as a prioritized candidate with the largest expression fold-change between HCC tumor and non-tumor samples from the TCGA-LIHC database (Fig. 1A).

To determine the clinical relevance of p38β, we performed immunohistochemical (IHC) analysis on a tissue microarray (TMA) comprising 136 paired HCC tumor (T) and adjacent non-tumor (N) liver samples. p38β expression was elevated in 53.68% of tumor tissues, compared to 28.68% of adjacent non-tumor tissues (Fig. 1B, C). Kaplan-Meier survival analysis further revealed that high p38β expression correlated with poorer overall survival in HCC patients (Fig. 1D). Moreover, elevated p38β levels were strongly associated with advanced disease features, including higher TNM stage, late HCC staging, vascular invasion (Fig. 1E–G and Supplementary Data 1), as well as moderately correlated with increased serum alpha-fetoprotein (AFP) levels (Fig. 1H and Supplementary Data 1). This association was supported by univariate Cox analysis (HR = 2.444, 95% CI: 1.572-3.798, *P* < 0.001), and remained significant in multivariate models adjusting for TNM stage and vascular invasion (Fig. 1I, J). These findings establish p38β as a clinically relevant kinase that is upregulated in HCC and may contribute to both disease progression and therapeutic resistance.

Consistent with the clinical findings, p38β protein level was moderately to highly upregulated in most HCC cell lines relative to immortalized normal liver cell line MIHA, with the highest expression observed in PLC8024 and Hep3B cells (Supplementary Fig. 1A, B). Sphere-formation assays revealed that PLC8024 (p38β-high) exhibited significantly higher self-renewal capacity than MHCC97L (p38β-low) (Supplementary Fig. 1C), while cell viability assays showed that PLC8024 cells were less sensitive to cisplatin, as indicated by a higher IC_50_ value (Supplementary Fig. 1D). Analysis of the CellMiner database (https://discover.nci.nih.gov/cellminer/) further revealed a moderate positive correlation between p38β expression and resistance to several chemotherapeutic agents, including cisplatin (CDDP) (Fig. 1K). To evaluate the functional relevance of p38β upregulation, we used two independent CRISPR-Cas9 single-guide RNAs (sgRNAs) to knockout p38β in PLC8024 cells. Western blot analysis confirmed efficient loss of p38β protein without affecting p38α (MAPK14) expression, indicating the specificity of the sgRNAs and excluding off-target silencing of the closely related p38α isoform (Fig. 1L and Supplementary Fig. 1E–G). Functionally, p38β knockout impaired proliferative potential and restored CDDP sensitivity (Fig. 1M, N). To determine whether this phenotype reflects a p38β-specific requirement rather than a p38 MAPK family-wide effect, we selectively silenced other p38 MAPK family members. Knockdown of p38γ (MAPK12), or p38δ (MAPK13) failed to alter foci formation and CDDP sensitivity in PLC8024 cells, whereas knockdown of p38α (MAPK14) showed a slight inhibitory effect on both phenotypes (Supplementary Fig. 1H–P), underscoring a non-redundant dependence on p38β. Together, these results indicate that p38β supports both the oncogenic and chemoresistant phenotypes of HCC cells.

### p38β-high HCC cells exhibit stemness characteristics

**Fig. 2:**
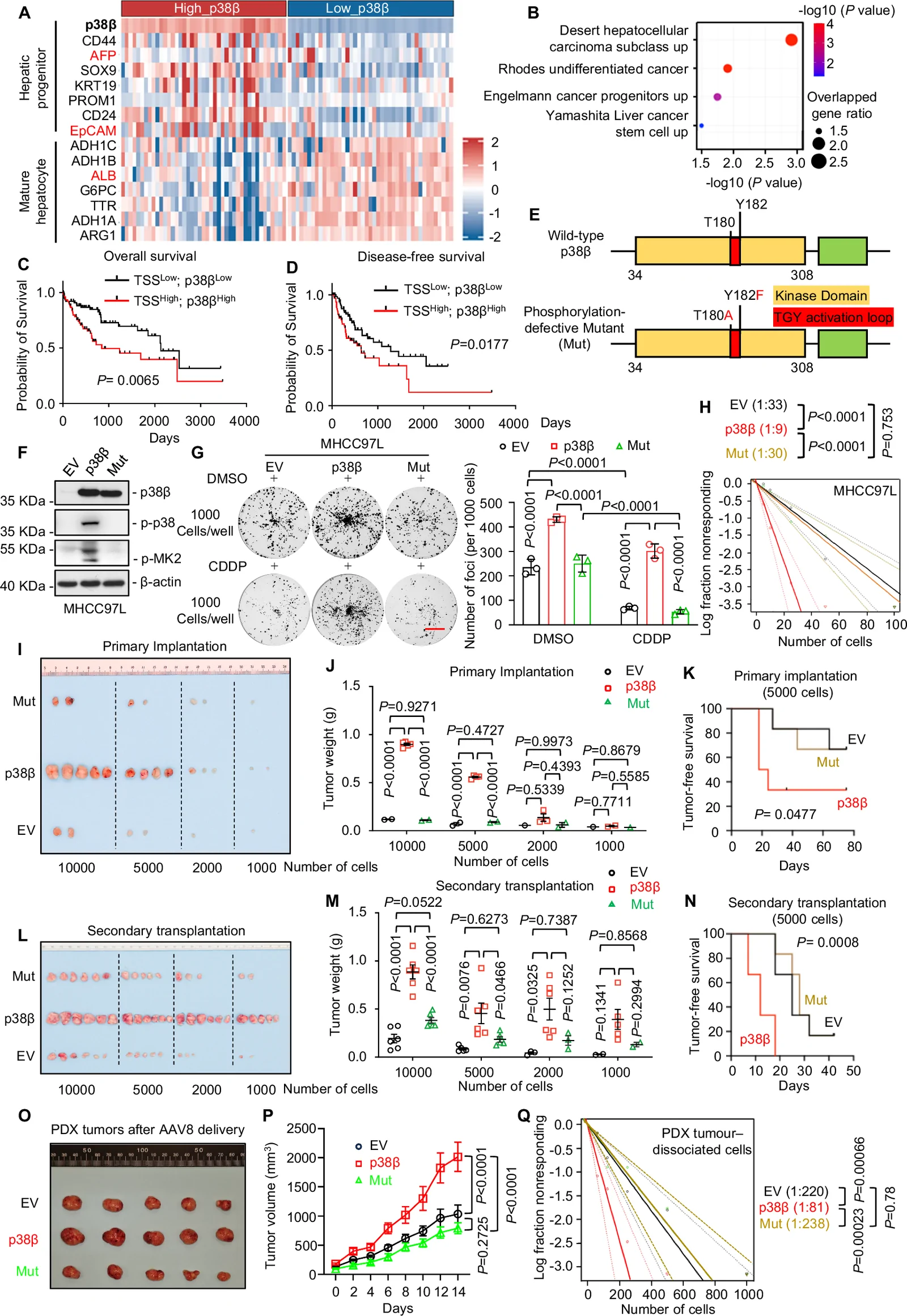
p38β-high HCC cells exhibit stemness characteristics. **A** Hepatic progenitor and mature hepatocyte markers in p38β-high versus p38β-low TCGA-LIHC tumors. **B** Gene set enrichment analysis (two‑sided) of TCGA HCC samples with high versus low p38β expression. **C**, **D** Kaplan–Meier analysis of overall (**C**) and disease-free survival (**D**) in HCC patients, stratified by p38β and TSS expression levels; median TSS expression was used as the cutoff. *n* = 157 patients (**A–D**). **E** Schematic of wild-type (WT) and phosphorylation-defective p38β mutant (Mut), in which threonine 180 (T180) and tyrosine 182 (Y182) in the TGY activation loop were replaced by alanine and phenylalanine, respectively. **F** Western blot analysis of p38β, phospho-p38 and phospho-MK2 in MHCC97L cells transfected with empty vector (EV), p38β WT, or Mut. The samples derive from the same experiment, but separate gels for p38β, p-p38, p-MK2 and β-actin were processed in parallel. **G** Foci formation assays in the indicated MHCC97L cells treated with DMSO or CDDP (10 μM). Right, quantification of foci numbers. Scale bar, 1 cm; *n* = 3 independent experiments. **H** Limiting dilution tumorsphere formation assays in the indicated MHCC97L cells (*n* = 3 independent experiments). **I–K** Primary limiting-dilution transplantation of MHCC97L cells expressing EV, WT or Mut into NOD/SCID mice at 10,000, 5000, 2000 or 1000 cells per mouse (*n* = 8 per group). Tumor incidence (**I**), tumor weight (**J**) and tumor-free survival (**K**) are shown for the 5000-cell group. **L–N** Serial secondary transplantation of dissociated primary tumors into NOD/SCID mice at limiting dilutions (*n* = 6 per group). **O**, **P** Representative images of tumors (**O**) and tumor growth curves (**P**) following intratumoral AAV8-mediated overexpression of EV, p38β WT or Mut in established HCC PDX tumors, as indicated. (*n* = 5 mice per group). **Q** ELDA plot comparing sphere-forming frequency in PDX tumor-dissociated cells (*n* = 6 mice per group). Data represent three independent experiments (**F**). Values are mean ± SD (**G**, **J**, **M**). Two‑sided log‑rank test, no multiple comparison adjustment (**C**, **D**, **K**, **N**). One-way (**G**, **J**, **M**) or two-way (**P**) ANOVA followed by Tukey’s multiple comparison. Two-sided ELDA likelihood-ratio test (**H**, **Q**).

To gain mechanistic insight into the oncogenic role of p38β in HCC, we compared the transcriptomic profiles in tumors with high versus low p38β expression from TCGA-LIHC dataset. Differential gene expression profiling revealed that high p38β tumors exhibited elevated levels of hepatic progenitor markers (e.g., AFP, SOX9 and EpCAM) and reduced mature hepatocyte markers (e.g., ALB, G6PC), indicative of a less differentiated, stem-like cellular state (Fig. 2A)^28,29^. Moreover, gene set enrichment analysis revealed that genes upregulated in the high p38β group were significantly enriched in transcriptional programs associated with cancer stemness and poor differentiation, including the Rhodes undifferentiated cancer profile, the Engelmann cancer progenitor signature, and Yamashita liver cancer stem cell signature (Fig. 2B). Kaplan-Meier survival analysis further demonstrated that patients with high tumor stemness scores (TSS^High^) and high p38β expression (p38β^High^) exhibited significantly poorer clinical outcomes compared to those with low levels of both markers (TSS^Low^; p38β^Low^) (Fig. 2C, D). In support of a functional role of p38β in tumor self-renewal capacity, both pharmacological and genetic approaches were applied. MHCC97L cells overexpressing p38β or EV control together with the silencing of p38α were treated with the p38α/β inhibitor SB203580, as a selective p38β inhibitor is not commercially available (Supplementary Fig. 2A–C). SB203580 treatment significantly reduced tumorsphere formation in p38β overexpressing cells with p38α silencing, but not in empty vector (EV) control (Supplementary Fig. 2D). CRISPR-mediated knockout of p38β in PLC8024 cells similarly reduced tumorsphere-initiating cell frequency, indicating impaired self-renewal capacity of HCC stem-like cells (Supplementary Fig. 2E). Together, these findings establish p38β as a key regulator of stemness and cellular dedifferentiation in HCC.

To further confirm the functional relevance and kinase activity of p38β in HCC, we generated wild-type p38β (p38β WT) and a phosphorylation-defective mutant (p38β Mut), in which the activating residues Thr180 (T180) and Tyr182 (Y182) were substituted with alanine (A) and phenylalanine (F), respectively, to prevent kinase activation (Fig. 2E). We then overexpressed either p38β WT or Mut in MHCC97L cells, which exhibit relatively low endogenous p38β expression among a panel of HCC cell lines (Supplementary Fig. 1A). We verified the functional integrity of the kinase activity in these constructs by assessing total and phosphorylated p38β levels, as well as the phosphorylation of MAPKAPK2 (MK2) which is a downstream substrate of p38β^30^, in MHCC97L cells overexpressing p38β WT or Mut. p38β WT significantly increased MK2 phosphorylation, whereas the Mut form had no detectable effect (Fig. 2F and Supplementary Fig. 2F–H), confirming that p38β WT is catalytically active and capable of engaging its downstream signaling cascade. Functionally, p38β WT overexpression, but not p38β Mut, promoted CDDP tolerance and self-renewal capacity in both 2D cell (Fig. 2G and Supplementary Fig. 3A) and 3D tumorsphere cultures (Fig. 2H and Supplementary Fig. 3B, C). Consistently, overexpression of p38β WT, but not Mut, promoted CDDP tolerance and tumorsphere initiating capacity in another low p38β-expressing HCC cell line Huh-7 (Supplementary Fig. 3D–G).

To assess the functional role of p38β in vivo, we conducted limiting dilution transplantation assays in NOD/SCID mice using MHCC97L cells overexpressing p38β WT, Mut, or EV (Supplementary Fig. 4A). In primary implantation, p38β WT cells displayed significantly increased TIC frequency, resulting in higher tumor incidence, larger tumor burden and shorter tumor-free survival, particularly at 5000-cell doses, compared with EV and p38β Mut groups (Fig. 2I–K and Supplementary Fig. 4B–D). Serial secondary transplantation further confirmed sustained tumor-initiating capacity in p38β WT-derived cells, whereas p38β Mut and EV cells exhibited significantly reduced engraftment potential across nearly all cell doses (Fig. 2L–N and Supplementary Fig. 4E–G). Consistently, ex vivo limiting dilution tumorsphere assays using primary tumor-derived cells confirmed a significantly higher TIC frequency in the p38β WT group (Supplementary Fig. 4H). Additional mouse model using adeno-associated virus serotype 8 (AAV8)-mediated transduction of p38β WT and Mut confirmed a stronger tumorigenic potential in p38β WT-overexpressed human patient-derived xenograft (PDX #2) (Fig. 2O, P) and an enhanced tumorsphere initiating capacity of resected p38β WT-overexpressed tumor cells (Fig. 2Q and Supplementary Fig. 4I). IHC staining of AAV8-transduced PDX tumors confirmed that p38β WT overexpression resulted in increased proliferative activity, as indicated by elevated PCNA staining in p38β WT group compared with EV and Mut groups (Supplementary Fig. 4J–L). Together, these findings establish p38β as a key regulator of tumor-initiating capacity in HCC and underscore the essential role of its kinase activity in sustaining oncogenicity and stem-like properties in vivo.

### p38β drives stemness and drug resistance in HCC via binding BiP

**Fig. 3:**
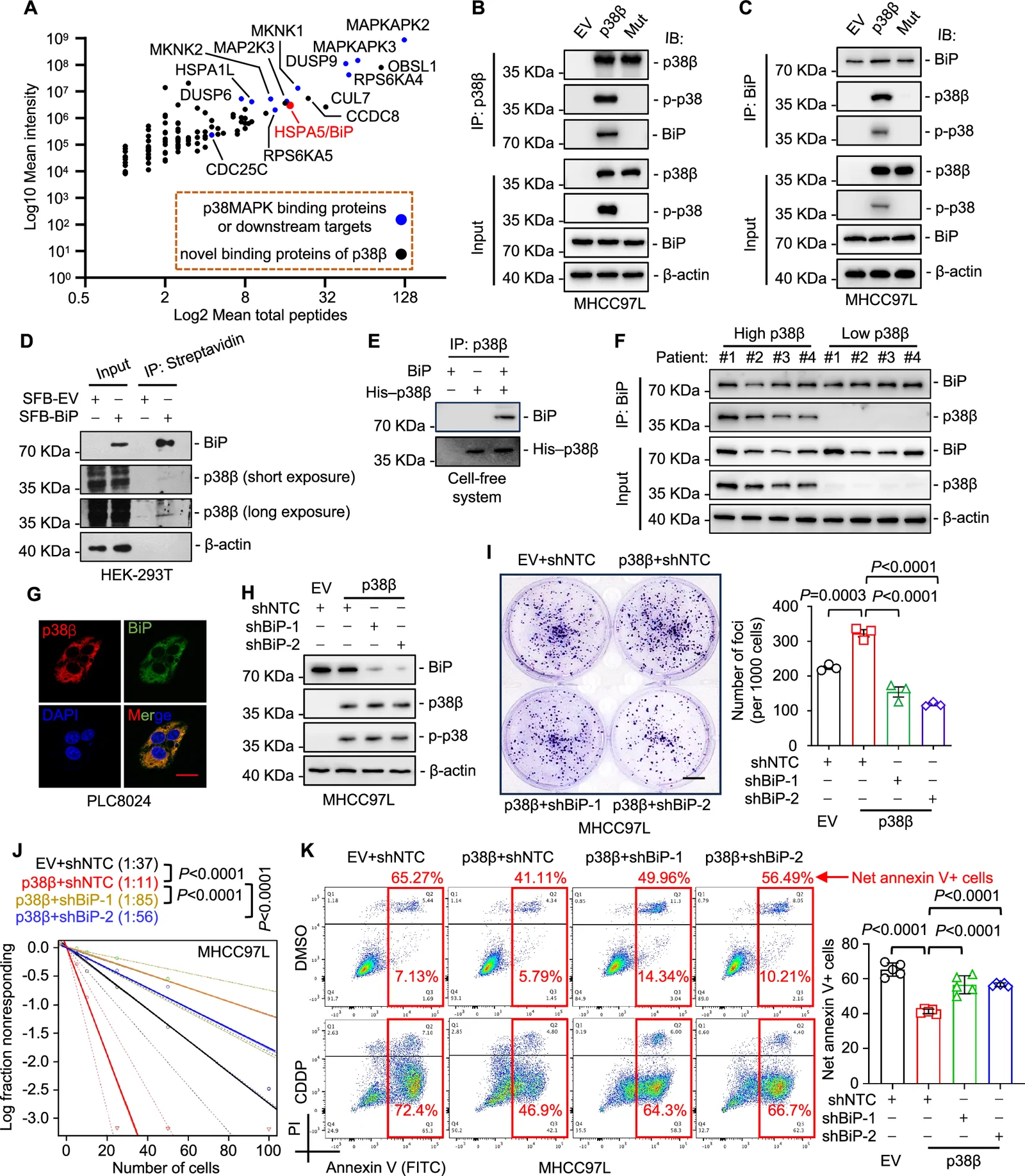
p38β drives stemness and drug resistance in HCC via binding BiP. **A** A Scatter plot showing the potential p38β-binding proteins identified through tandem affinity purification-mass spectrometry (TAP-MS) analysis. **B**, **C** Co-IP assays in MHCC97L cells expressing EV, p38β WT, or Mut, performed by pulldown of either p38β (**B**) or BiP (**C**), demonstrating their interaction. **D** Streptavidin pulldown assays were performed to analyze the interaction between p38β and BiP in HEK-293T cells transfected with SFB-BiP. **E** In vitro binding assays assessed the direct interaction between p38β and BiP using purified recombinant proteins. **F** Endogenous p38β and BiP were immunoprecipitated from patient-derived HCC tissues exhibiting either high or low p38β expression. **G** Representative immunofluorescence images showing co-localization of p38β and BiP in PLC8024 cells. Scale bar, 10 μm. **H** Western blot analysis of BiP, p38β expression, and p38β phosphorylation in MHCC97L HCC cells overexpressing p38β, with or without BiP knockdown. For (**B–F**, **H**), samples within each experiment derived from the same experiment, but separate gels for the indicated proteins and β-actin, where applicable, were processed in parallel. **I**, **J** Foci formation (**I**) and limiting dilution tumorsphere formation assays (**J**) revealed that BiP knockdown reversed the increase in oncogenic and self-renewal capacity caused by p38β overexpression in HCC cells. Right, quantification of foci numbers (**I**). Scale bar, 1 cm; *n* = 3 independent experiments. **K** Flow cytometry analysis showed that BiP knockdown restored the reduced proportion of Annexin V positive cells caused by p38β overexpression in MHCC97L cells after CDDP (10 μM, 24 h) treatment (*n* = 5). Data represent three independent experiments (**B–H**). Values are mean ± SD (**I**, **K**). one-way ANOVA followed by Tukey’s multiple comparison (**I**, **K**). Two-sided ELDA likelihood-ratio test (**J**).

To elucidate the mechanism by which elevated p38β drives stemness and chemoresistance in HCC, we performed tandem affinity purification coupled with mass spectrometry (TAP-MS) on whole-cell lysates from HEK293T cells expressing tagged p38β, aiming to identify its potential interacting proteins. This proteomic analysis identified multiple reported p38 targets and interacting proteins, including MAPKAPK2/3, RPS6KA4/5, MKNK1/2, and DUSP6/9 (Fig. 3A and Supplementary Data 2). Among the putative p38β-interacting proteins, HSPA5 (BiP), which is an ER-resident chaperone, was prioritized as one of the previously unreported binding proteins of p38β, exhibiting comparable intensity and peptide numbers to those of established p38β interactors (Fig. 3A). BiP is a master regulator of the UPR and has been implicated in the maintenance of cancer stem-like traits and chemoresistance across multiple tumor types^11,31^. Given the documented oncogenic functions of BiP and the availability of a specific inhibitor HA15, we proposed that BiP may represent a potential downstream target of p38β with therapeutic translation potential. Co-immunoprecipitation (Co-IP) assays validated the interaction between p38β and BiP in MHCC97L cells (Fig. 3B, C and Supplementary Fig. 5A–L). Notably, a kinase-dead mutant of p38β (Mut) failed to bind BiP, suggesting that the interaction depends on p38β kinase activity (Fig. 3B, C and Supplementary Fig. 5A–L). To confirm this interaction from the BiP side, we overexpressed S-protein-FLAG-Streptavidin-binding peptide (SFB)-tagged BiP and performed streptavidin-based pull-down assay, which further verified the association between BiP and endogenous p38β (Fig. 3D and Supplementary Fig. 5M, N). To determine whether this interaction is direct rather than mediated by additional cofactors, we next performed a cell-free in vitro binding assay using recombinant His-tagged p38β and BiP proteins. Consistent with the cellular Co-IP results, BiP can be pulled down together with p38β in vitro, demonstrating a direct physical interaction independent of other cellular components (Fig. 3E and Supplementary Fig. 5O, P). Importantly, using lysates from patient-derived HCC tissues with either high or low p38β expression, we further found that BiP co-precipitated more efficiently with p38β in high-expression samples, supporting the physiological relevance of this interaction (Fig. 3F and Supplementary Fig. 5Q–U). To assess whether this interaction is spatially feasible within cells, we conducted immunofluorescence staining followed by confocal microscopy. Confocal imaging revealed colocalization of BiP and p38β within the cytoplasmic region (Fig. 3G). These observations support the spatial concordance of a cytosolic or ER-associated pool of BiP and p38β. To further determine whether this interaction is isoform-specific, we performed parallel co-IP assay targeting p38α (MAPK14). Under identical experimental condition, p38α failed to pull down BiP in PLC8024 cells (Supplementary Fig. 5V–X). These findings establish an isoform-specific role for p38β with BiP interaction.

To determine whether p38β drives stemness and chemoresistance in HCC through BiP, we silenced BiP using two independent short hairpin RNAs (shRNAs) in p38β-overexpressing MHCC97L cells (Fig. 3H; Supplementary Fig. 5Y-AA). Knockdown of BiP significantly reduced p38β-driven foci formation and tumorsphere initiating capacity (Fig. 3I, J and Supplementary Fig. 6A). Moreover, BiP silencing could reverse CDDP resistance induced by p38β overexpression, as evidenced by increased apoptosis in Annexin V/PI flow cytometry analysis (Fig. 3K). To further confirm these findings, we treated both control (EV) and p38β-overexpressing MHCC97L cells with HA15, which is a selective BiP inhibitor that impairs its chaperone function and triggers sustained UPR activation. HA15 treatment phenocopied the effects of BiP knockdown, significantly reducing foci formation, decreasing tumorsphere initiating frequency, and restoring CDDP sensitivity in p38β-overexpressing cells (Supplementary Fig. 6B–D). Together, these findings support that p38β confers HCC self-renewal and chemoresistance through BiP.

### BiP Thr648 phosphorylation is essential for p38β-driven stemness and drug resistance in HCC

**Fig. 4:**
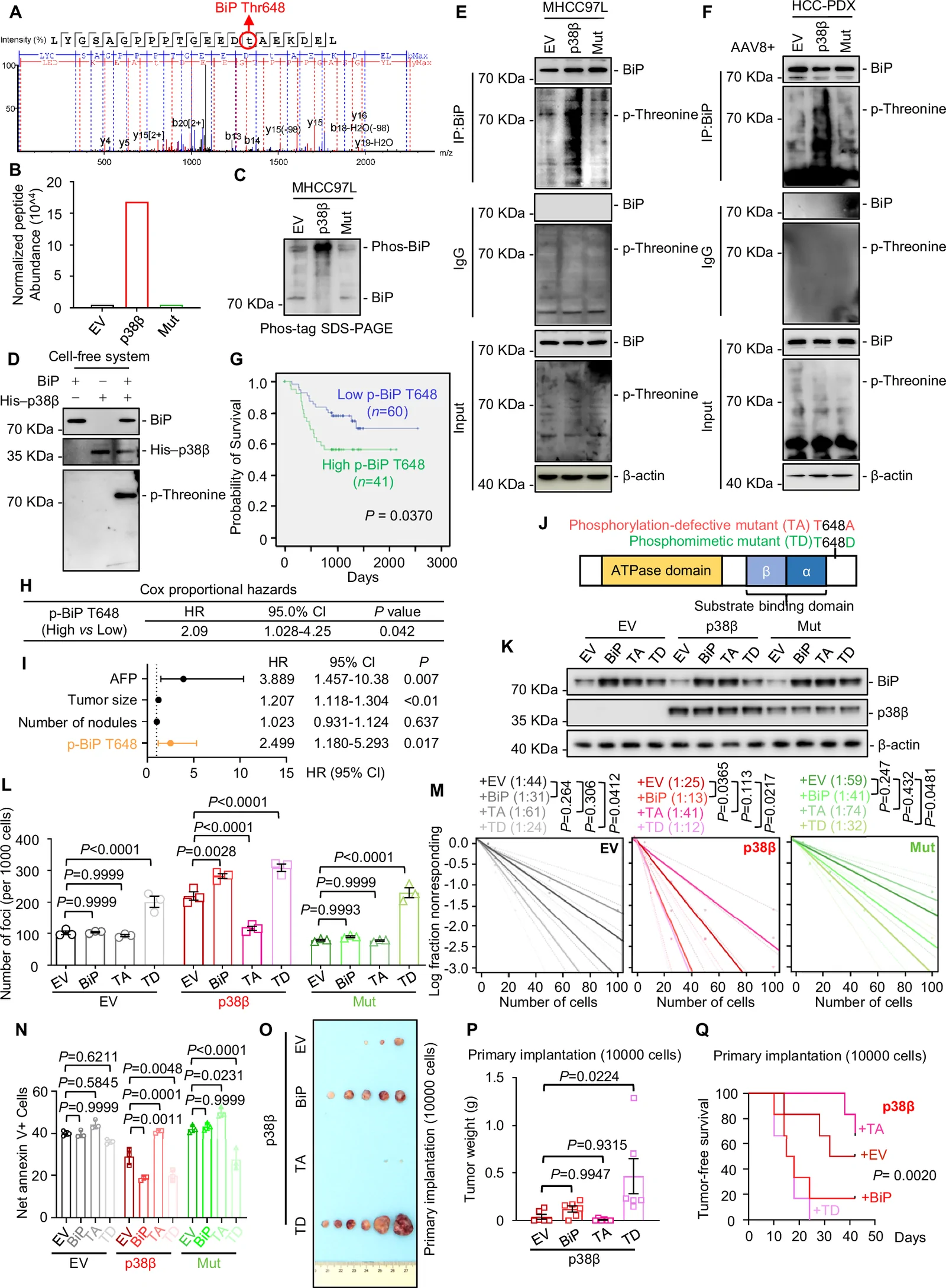
BiP Thr648 phosphorylation is essential for p38β-driven stemness and drug resistance in HCC. **A** Representative MS/MS spectrum of the phosphopeptide LYGSAGPPPTGEEDtAEKDEL (t, phosphorylated threonine), confirming BiP Thr648 phosphorylation. **B** Quantitative phosphoproteomic analysis of the BiP Thr648 phosphopeptide based on normalized log2 MS1 intensities. **C** Phosphate-affinity (Phos-tag) SDS-PAGE analysis of BiP phosphorylation in the indicated MHCC97L cells. **D** In vitro kinase assays assessing p38β-mediated BiP phosphorylation using purified recombinant proteins, and pan-phospho-threonine immunoblotting. **E**, **F** BiP threonine phosphorylation in the indicated MHCC97L cells and HCC PDX tumors, assessed by BiP immunoprecipitation and pan-phospho-threonine immunoblotting. **G** Kaplan–Meier overall survival analysis of the HCC phosphoproteomic cohort (PDC000199) stratified by BiP Thr648 phosphorylation. **H** Univariate two-sided Cox proportional hazards analysis of overall survival stratified by p-BiP T648 expression (High *vs* Low); HR and 95% CI are shown, without multiple comparison adjustment. **I** Multivariable two‑sided Cox regression analysis (no multiple comparison adjustment) including AFP, Tumor size, Number of nodules and p-BiP T648. Forest plot shows HR with 95% CI; corresponding estimates are provided. *n* = 101 patients (**G–I**). **J** Schematic illustration of Phosphorylation-defective BiP T648A (TA) and phosphomimetic T648D (TD) constructs. **K** Immunoblotting of BiP and p38β in the indicated MHCC97L cells. For (**D-F**, **K**), samples within each experiment derived from the same experiment, but separate gels for the indicated proteins and controls, where applicable, were processed in parallel. **L**, **M** Foci formation (**L**) and Limiting dilution tumorsphere formation (**M**) assays in the indicated cells (*n* = 3 independent experiments). **N** Annexin V flow-cytometric analysis of apoptosis following DMSO or CDDP treatment (10 μM, 24 h; *n* = 3 independent experiments). **O-Q** Limiting-dilution transplantation of p38β-overexpressing MHCC97L cells expressing EV, BiP, TA or TD into NOD/SCID mice. Tumor incidence (**O**), tumor weight (**P**) and tumor-free survival (**Q**) are shown for the 10,000-cell group. (*n* = 6 mice per group). Data represent three independent experiments (**C–F**, **K**). Values are mean ± SD (**L**, **N**, **P**). One-way ANOVA followed by Tukey’s multiple-comparisons test (**L**, **N**, **P**). Two-sided ELDA likelihood-ratio test (**M**). Two-sided log-rank test, with no multiple-comparison adjustment (**G**, **Q**).

Post-translational modifications, particularly phosphorylation, are critical regulatory mechanisms that underpin kinase-driven chemoresistance across various cancer types^32–34^. Given that p38β is a serine/threonine kinase, we hypothesized that it may promote HCC stemness and chemoresistance by directly modulating BiP activity through phosphorylation. We first examined BiP expression levels and found that p38β knockout did not significantly alter total BiP protein abundance (Supplementary Fig. 7A, B). Building on our finding that p38β directly and functionally engages BiP, we next sought to determine whether p38β regulates BiP through phosphorylation. To this end, phosphoproteomic profiling in p38β-overexpressing MHCC97L cells uncovered a robust increase in phosphorylation of BiP at threonine 648 (Thr648, T648), a modification not previously associated with p38β signaling (Fig. 4A, B and Supplementary Fig. 7C and Supplementary Data 3). To validate this modification in HCC cells, we employed phosphate-affinity (Phos-tag) SDS-PAGE to directly visualize phosphorylation-dependent mobility shifts of BiP in p38β-overexpressing MHCC97L cells. BiP displayed a significantly slower-migrating band in p38β-overexpressing cells compared with EV and Mut, consistent with the expected shift caused by phosphorylation (Fig. 4C and Supplementary Fig. 7D). To validate whether BiP is directly phosphorylated by p38β, an in vitro kinase assay using an activated recombinant p38β and BiP proteins demonstrated that p38β could directly phosphorylate BiP at Threonine residue (Fig. 4D and Supplementary Fig. 7E). Consistently, BiP immunoprecipitation followed by immunoblotting with a pan-phospho-Threonine antibody revealed increased threonine phosphorylation of immunoprecipitated BiP upon p38β WT overexpression, but not upon overexpression of the kinase-dead Mut (Fig. 4E and Supplementary Fig. 7F). In vivo delivery of AAV8-p38β-WT into established HCC PDX #2 tumors resulted in detectable threonine phosphorylation of immunoprecipitated BiP, which was significantly reduced in tumors expressing kinase-dead p38β Mut or EV (Fig. 4F and Supplementary Fig. 7G). In addition, comparison between HCC cell lines further supported this association, as PLC8024 cells with high endogenous p38β expression exhibited stronger BiP phosphorylation signal than MHCC97L cells, which express relatively low levels of p38β (Supplementary Fig. 7H, I). Importantly, p38β-dependent binding and phosphorylation were not observed on the abundant HSP70 isoform HSPA8 (Supplementary Fig. 7J–N), supporting p38β’s selectivity for BiP rather than a general HSP70-family effect. Together, these results demonstrate that BiP threonine phosphorylation is driven by catalytically active p38β in HCC cells and in vivo HCC PDX model.

We next explored the clinical relevance of this modification. Kaplan-Meier survival analysis further revealed that high p-BiP T648 expression correlated with poorer overall survival in HCC patients (Fig. 4G). This association was supported by univariate Cox analysis (HR = 2.09, 95% CI: 1.028–4.25, *P* = 0.042), and remained significant in multivariate models adjusting for tumor size and AFP level (Fig. 4H, I). To further validate the functional significance of BiP phosphorylation at Thr648, we generated wild-type (BiP), phosphorylation-deficient (T648A, TA), and phosphomimetic (T648D, TD) BiP constructs (Fig. 4J), and ectopically expressed them in MHCC97L cells overexpressing EV, p38β WT, or kinase-dead p38β Mut. Expression of BiP and p38β was confirmed by Western blotting (Fig. 4K and Supplementary Fig. 7O, P). Functional assays revealed that overexpression of wild-type BiP and TD mutant significantly enhanced foci formation and tumorsphere initiating capacity, indicative of increased oncogenicity and self-renewal capacity (Fig. 4L, M and Supplementary Fig. 8A, B). In contrast, TA mutant suppressed these phenotypes, particularly in the group of p38β overexpression (Fig. 4L, M and Supplementary Fig. 8A, B). Additionally, wild-type BiP and TD mutant significantly enhanced CDDP resistance induced by p38β overexpression, whereas TA mutant failed to promote chemoresistance, as evidenced by decreased apoptosis in Annexin V/PI flow cytometry analysis (Fig. 4N and Supplementary Fig. 8C). To evaluate whether BiP Thr648 phosphorylation also regulates tumor-initiating capacity in vivo, we performed limiting dilution transplantation assays in NOD/SCID mice using MHCC97L cells expressing EV, BiP, TA, or TD mutants in the context of p38β WT overexpression. Consistent with in vitro results, wild-type BiP and TD mutant significantly increased tumor-initiating frequency and tumor burden, while TA mutant failed to enhance these phenotypes (Fig. 4O–Q and Supplementary Fig. 8D–F). Collectively, these findings suggest that BiP Thr648 phosphorylation is required for p38β-driven stemness and drug resistance.

### p38β-driven stemness and drug resistance via BiP-dependent UPR signaling in HCC

**Fig. 5:**
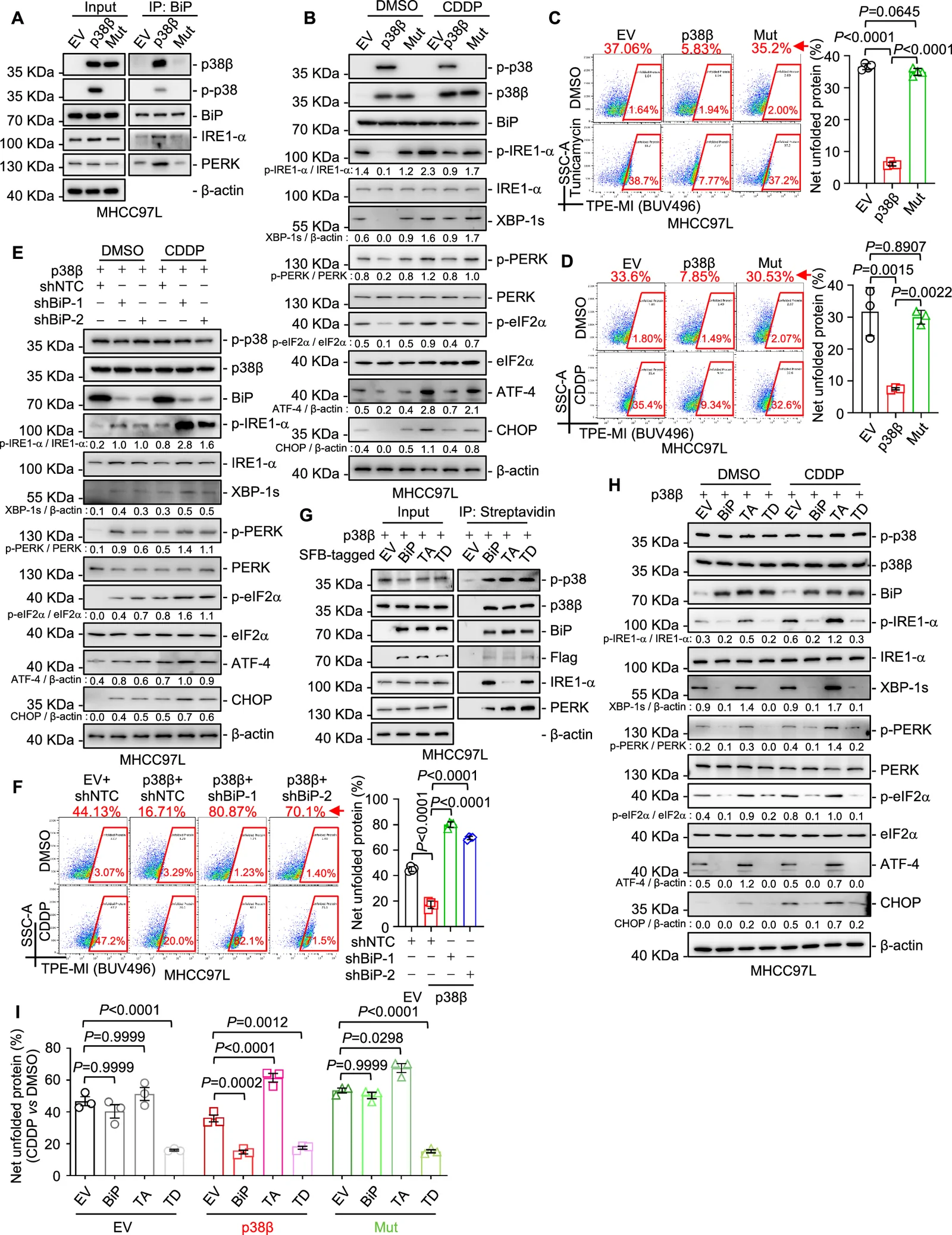
p38β-driven stemness and drug resistance via BiP-dependent UPR signaling in HCC. **A** IP assays in MHCC97L cells expressing EV, p38β WT, or Mut were performed by pulling down BiP to assess its interaction with IRE1-α or PERK. **B** Western blot analysis of UPR pathway activation in MHCC97L cells expressing EV, p38β WT, or Mut, following treatment with DMSO or CDDP (10 µM, 24 h). **C**, **D** Flow cytometry analysis using TPE-MI (BUV496) demonstrated that p38β-overexpressing MHCC97L cells exhibited significantly reduced unfolded protein accumulation following treatment with either Tunicamycin (0.5μg/mL, 24 h; *n* = 3 independent experiments) (**C**) or CDDP (10 µM, 24 h; *n* = 3 independent experiments) (**D**). This reduction was not observed in cells expressing EV or Mut. Tunicamycin served as a positive control for unfolded protein accumulation. **E** Western blot analysis of UPR pathway activation in the indicated MHCC97L cells treated with DMSO or CDDP (10 µM, 24 h). **F** Flow cytometry analysis using TPE-MI measured unfolded protein accumulation in p38β-overexpressing MHCC97L cells with or without BiP knockdown, following treatment with DMSO or CDDP (10 µM, 24 h; *n* = 4 independent experiments). **G** Streptavidin pulldown assays demonstrated that SFB-tagged BiP and its TD mutant, but not the TA mutant, enhanced the interaction between BiP and IRE1-α in p38β-overexpressing MHCC97L cells. **H** Western blot analysis of UPR pathway activation in the indicated MHCC97L cells treated with DMSO or CDDP (10 µM, 24 h). For (**A**, **B**, **E**, **G**, **H**), samples within each experiment derived from the same experiment, but separate gels for the indicated proteins and controls were processed in parallel. **I** Flow cytometry analysis of unfolded protein accumulation in the indicated MHCC97L cells treated with DMSO or CDDP (10 µM, 24 h), using TPE-MI staining (*n* = 3 independent experiments). Data represent three independent experiments (**A**, **B**, **E**, **G**, **H**). Values are mean ± SD (**C**, **D**, **F**, **I**). One-way ANOVA followed by Tukey’s multiple comparison (**C**, **D**, **F**, **I**).

BiP serves as a master regulator of the UPR by binding to and suppressing ER stress sensors, including IRE1-α, PERK, and ATF6, under homeostatic conditions^9,11^. Upon ER stress, BiP dissociates from these sensors, permitting their activation and initiation of downstream UPR signaling^11^. Due to undetectable endogenous ATF6 levels in our system, we focused on the IRE1-α and PERK pathways. Co-IP assays revealed that p38β overexpression enhanced BiP interaction with both IRE1-α and PERK, whereas this enhancement was not observed in the kinase-dead Mut group, indicating that p38β regulates BiP-mediated UPR in a kinase activity-dependent manner (Fig. 5A and Supplementary Fig. 9A–E). Given that BiP binding inhibits UPR sensor activation under homeostatic conditions, we examined the status of the proximal UPR branches. Western blot analysis showed that p38β overexpression significantly suppressed phosphorylation of both IRE1α and PERK under basal (DMSO) and chemotherapeutic (CDDP) conditions, indicating attenuation of upstream UPR sensor activation (Fig. 5B and Supplementary Fig. 9F–K). Consistently, downstream PERK–eIF2α–ATF4 signaling was blunted, as shown by reduced phospho-eIF2α and diminished ATF4 and CHOP induction, together with significantly decreased XBP1 splicing (Fig. 5B and Supplementary Fig. 9F–K). Conversely, p38β knockout produced the opposite effects, with robust increases in p-IRE1α, p-PERK, p-eIF2α, ATF4, CHOP, and spliced XBP1 (Supplementary Fig. 9L–R), further supporting a model in which loss of p38β relieves BiP-mediated suppression of UPR sensors and potentiates both PERK and IRE1α branches. These findings collectively demonstrate that p38β signaling broadly restrains UPR activation.

To functionally evaluate whether p38β affects ER proteostasis, we quantified unfolded protein levels using flow cytometry with tetraphenylethene maleimide (TPE-MI), a thiol-reactive dye that preferentially binds exposed cysteines in unfolded proteins^35^. Tunicamycin, a well-characterized ER stress inducer, was used as a positive control and significantly increased TPE-MI signal intensity (Fig. 5C). p38β-overexpressing cells exhibited significantly reduced TPE-MI fluorescence following both Tunicamycin and CDDP treatment, indicating a decreased burden of unfolded proteins (Fig. 5C, D). This reduction was not observed in cells expressing EV or p38β Mut (Fig. 5C, D). In addition, p38β WT overexpression significantly reduced Tunicamycin-induced apoptosis compared with EV and Mut (Supplementary Fig. 9S). Notably, p38β knockout significantly increased TPE-MI fluorescence under Tunicamycin treatment, reinforcing the notion that p38β suppresses unfolded protein accumulation during ER stress (Supplementary Fig. 9T). Together, these findings suggest that p38β signaling suppresses UPR activation, potentially by limiting the accumulation of unfolded proteins in response to chemotherapeutic stress.

To confirm whether the suppressive effect of p38β on UPR signaling is mediated through BiP, we next silenced BiP in p38β-overexpressing MHCC97L cells. Western blot analysis revealed that BiP silencing restored the phosphorylation of IRE1-α and PERK, together with reactivation of downstream effectors of both UPR branches, including increased phospho-eIF2α, enhanced ATF4 and CHOP induction, and elevated XBP1 mRNA splicing that had been suppressed by p38β overexpression, particularly under CDDP treatment conditions (Fig. 5E and Supplementary Fig. 10A–F). Consistently, flow cytometry using TPE-MI demonstrated that BiP knockdown increased the levels of unfolded proteins in p38β-overexpressing cells following both Tunicamycin and CDDP exposure, reversing the proteostasis-suppressive phenotype conferred by p38β (Fig. 5F and Supplementary Fig 10G). To further verify these findings, we treated control (EV) and p38β-overexpressing MHCC97L cells with HA15, a selective BiP inhibitor. HA15 treatment phenocopied the effects of BiP knockdown, pharmacologically reversing p38β-mediated suppression of UPR signaling in p38β-overexpressing cells (Supplementary Fig. 10H–N).

As we previously demonstrated that p38β regulates BiP-mediated UPR signaling in a kinase activity-dependent manner, we next investigated whether this effect is mediated through BiP phosphorylation. We overexpressed BiP, TA, or TD in MHCC97L cells overexpressing p38β WT. Streptavidin pull-down assays demonstrated that both wild-type BiP and TD mutant exhibited enhanced binding to IRE1-α compared to the TA mutant (Fig. 5G and Supplementary Fig. 10O-S), indicating that phosphorylation of BiP at Thr648 promotes its interaction with IRE1-α downstream of p38β. Consistently, Western blot analysis revealed that overexpression of wild-type BiP or TD mutant significantly suppressed phosphorylation of UPR sensors IRE1α and PERK, particularly under CDDP treatment in the context of p38β overexpression (Fig. 5H and Supplementary Fig. 11A–F). This suppression extended to downstream effectors of both UPR branches, including decreased p-eIF2α, attenuated ATF4 and CHOP induction, and significantly reduced XBP1 mRNA splicing. In contrast, TA mutant failed to inhibit UPR sensor phosphorylation, showing levels comparable to EV controls and the kinase-dead p38β Mut background (Fig. 5H and Supplementary Fig. 11A-F). These findings demonstrate that BiP phosphorylation at Thr648 is required for p38β-mediated repression of both PERK-eIF2α-ATF4 and IRE1α-XBP1 signaling.

Flow cytometry using TPE-MI demonstrated that, in the context of p38β overexpression, MHCC97L cells expressing wild-type BiP or TD mutant exhibited significantly lower fluorescence intensity than EV or TA-expressing cells under CDDP (Fig. 5I and Supplementary Fig. 11G) or Tunicamycin exposure (Supplementary Fig. 11H), indicating reduced accumulation of unfolded proteins during ER stress. Under the same experimental conditions, apoptosis was quantified by flow cytometry using Annexin V/PI staining, which revealed that wild-type BiP and TD significantly decreased the proportion of apoptotic cells compared with EV or TA-expressing cells following CDDP treatment (Fig. 4N). These findings support that BiP Thr648 phosphorylation not only suppresses unfolded protein burden but also confers a functional survival advantage under ER stress. Together, our data support a model in which p38β promotes BiP phosphorylation at Thr648, enhancing its interaction with the UPR sensor to attenuate UPR activation and thereby reduce unfolded protein accumulation and apoptosis.

### Targeting the p38β-BiP axis enhances CDDP sensitivity in PDX and PDO models

**Fig. 6:**
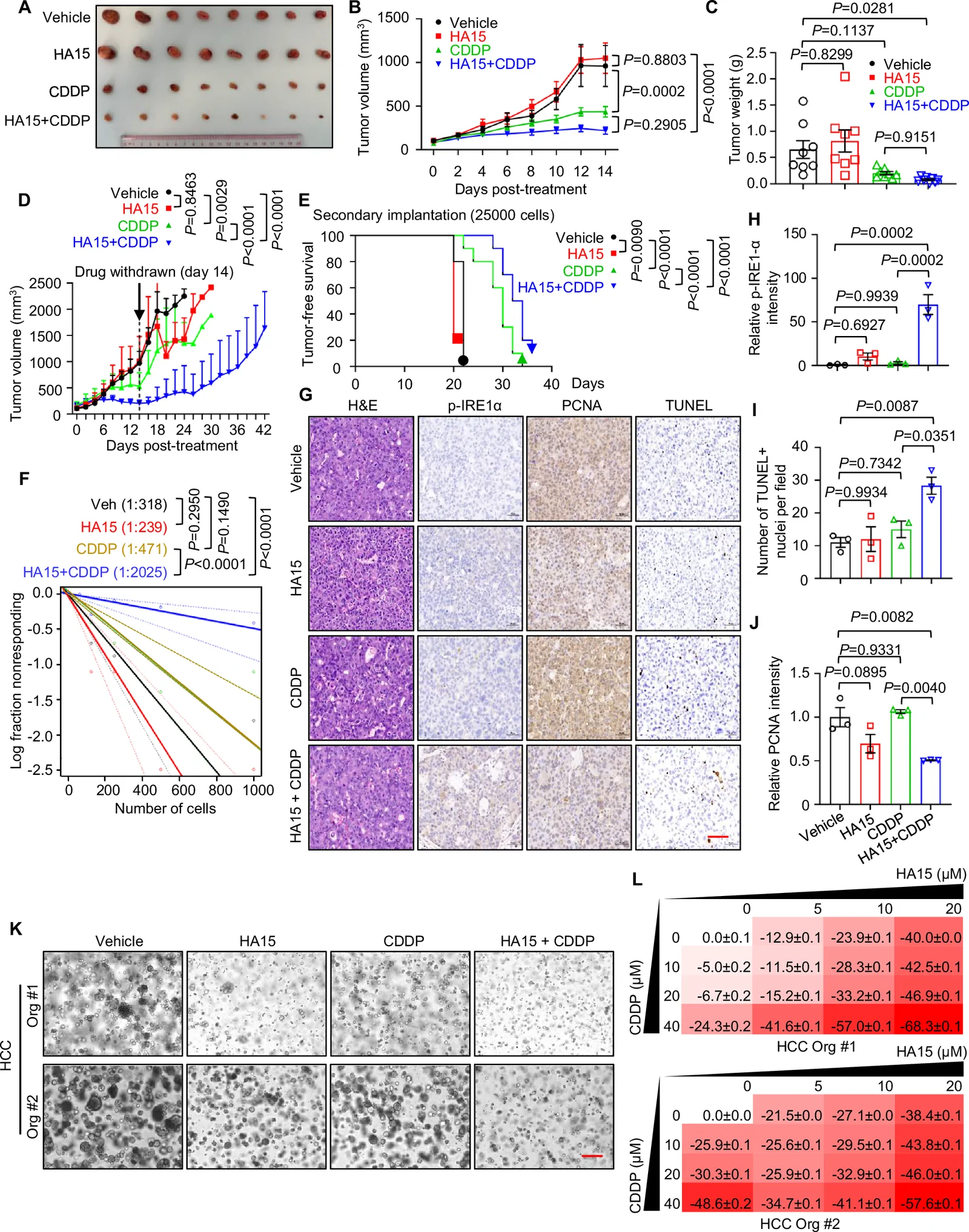
Targeting the p38β–BiP axis enhances CDDP sensitivity in PDX and PDO models. **A–C** Representative images (**A**), tumor growth curves (**B**), and tumor weights (**C**) of patient-derived xenografts (PDXs) treated with vehicle, HA15 (10 mg/kg, intraperitoneally [*i.p*.], every 2 days for 2 weeks), CDDP (5 mg/kg, i.p., every 2 days for 2 weeks), or the combination of HA15 and CDDP. *n* = 8 mice per group. **D** Tumor growth curves from an independent repeated PDX cohort incorporating a treatment-withdrawal: after the 14-day treatment phase, drugs were withdrawn, and tumor regrowth was monitored longitudinally. *n* = 8 mice per group. **E** Tumor-free survival of recipient mice after secondary implantation of tumor cells derived from the human PDX models shown in Fig. 6A and exposed to the indicated treatments. **F** Extreme Limiting Dilution Analysis (ELDA) of ex vivo limiting-dilution spheroid formation assay of dissociated tumor cells from human PDX models shown in Fig. 6A, with plating densities at 1,000, 500, 250, 125, and 62.5 cells per well (*n* = 6 mice per group). **G–J** IHC staining and quantification of IRE1-α (**G**, **H**), TUNEL (**G**, **I**) and PCNA (**G**, **J**) in PDXs subjected to indicated treatments. Representative IHC images are shown in (**G**). Scale bar, 50 µm. **K** Representative images of two independent patient-derived organoids (PDOs; labeled Org#1 and Org#2) treated with vehicle, HA15, CDDP, or the combination of HA15 and CDDP. Scale bar, 100 µm. **L** Raw dose-response matrix data presented as a table for two individual HCC PDOs. The numbers within the table represent the mean organoid viability compared against DMSO control ± SD (*n* = 3 replicates per condition). Data are presented as mean ± SD (**C**, **H**–**J**). One-way (**C**, **H**–**J**) or two-way ANOVA followed by Tukey’s multiple comparison (**B**, **D**). Two‑sided log‑rank test, no multiple comparison adjustment (**E**). Two-sided ELDA likelihood-ratio test (**F**).

To assess the therapeutic potential of targeting the p38β-BiP axis in a clinically relevant context, we adopted PDX#1 and two independent patient-derived organoids (PDO Org#1 and Org#2) models from advanced HCC specimens (Patient #1, #2, and #3 with high p38β expression in Fig. 3F). In the PDX model, tumor-bearing mice were randomized into four treatment groups: vehicle, HA15, CDDP and HA15 + CDDP combination (Fig. 6A). Tumor growth was monitored during a 14-day treatment phase. Both CDDP monotherapy and HA15 + CDDP combination therapy could suppress tumor growth during the on-treatment window (Fig. 6A–C). However, upon treatment withdrawal and longitudinal follow-up, HA15 + CDDP combination treatment group exhibited a more durable suppression of tumor regrowth compared with CDDP alone, demonstrating an impaired tumor re-initiation capacity (Fig. 6D). Moreover, secondary implantation of residual tumor cells into new recipient mice resulted in prolonged tumor-free survival in the HA15 + CDDP combination group (Fig. 6E). Consistently, limiting-dilution tumorsphere formation assays using residual PDX-derived tumor cells showed that the combination treatment significantly reduced tumorsphere-initiating frequency relative to CDDP alone (Fig. 6F and Supplementary Fig. 12A). Supporting these functional readouts, flow cytometry analysis revealed lower percentages of TICs markers CD133 and EpCAM in the combination group (Supplementary Fig. 12B). Mechanistically, IHC analyses showed increased p-IRE1α, reduced PCNA, and elevated TUNEL staining in HA15 + CDDP combination group, consistent with enhanced UPR activation, reduced proliferation, and increased apoptosis (Fig. 6G–J).

To determine whether this chemosensitizing effect is recapitulated in an ex vivo patient-derived setting, we next evaluated HA15 and CDDP in PDOs. The combination significantly suppressed PDO growth compared with either monotherapy, as visualized by bright-field imaging (Fig. 6K). We then quantified the drug interaction using a dose-response matrix followed by SynergyFinder analysis^36^, which revealed dose-dependent positive synergy predominantly at intermediate-to-high concentrations, with strongly positive Bliss and ZIP scores ( ~ 35–46) and no evidence of antagonism across the tested PDOs (Fig. 6L; Supplementary Fig. 12C). Both PDO lines exhibited consistent synergistic reductions in viability with combined HA15 and cisplatin treatment, though the magnitude of synergy varied slightly between organoid lines. To evaluate therapeutic selectivity, we extended the combination testing to normal human liver epithelial cell lines MIHA and THLE-3, in which viability was only modestly reduced (remaining > 50%) within the synergy-associated concentration range (e.g., CDDP 10 µM + HA15 5 µM) (Supplementary Fig. 13A, B). Moreover, treatment in normal human hepatocyte-derived organoids showed no overt hepatotoxicity at synergy-associated concentrations, supporting a favorable therapeutic window (Supplementary Fig. 13C). Together, these findings support that pharmacological targeting of the p38β-BiP axis sensitizes HCC tumors to chemotherapy by restoring UPR signaling and disrupting tumor stemness.

## Discussion

Therapeutic resistance remains a formidable challenge in HCC, largely driven by TICs that evade cytotoxic stress and fuel disease relapse^37,38^. While stress-adaptive responses are increasingly recognized as hallmarks of aggressive tumors^39,40^, the molecular pathways that integrate ER stress and TIC maintenance remain poorly defined. Here, we identify p38β as a key regulator of stemness and chemoresistance, acting through a previously unrecognized p38β-BiP axis that governs ER proteostasis.

Our kinome-focused transcriptomic analysis, combined with DepMap (DEMETER2) dependency scores, prioritized p38β in chemotherapy-enriched spheroids. Although p38α is the best-characterized p38 MAPK isoform and a canonical mediator of cellular stress signaling^23,41^, its functions in cancer are highly context dependent. p38α activation can enforce tumor-suppressive programs, such as p53-dependent cell-cycle arrest^42^, senescence^43^, and DNA-damage responses^44^, and genetic deletion of p38α accelerates tumor initiation in multiple tissues^45^. Conversely, in established cancers, p38α can promote metabolic adaptation, invasion, and therapy resistance, reflecting its ability to support tumor survival under chronic stress^41^. Given this duality, recent studies have emphasized the importance of isoform-specific dissection of p38 MAPK biology, as many phenotypes historically attributed to “p38 MAPK” are now recognized to be isoform-selective and not exclusively driven by p38α. In contrast to p38α, p38β possesses distinct biochemical and regulatory properties, including cis-autophosphorylation, prolonged activation under chronic stress, and divergent substrate and scaffolding interactions^24,46,47^^,^. These differences suggest non-redundant signaling architectures, yet the contribution of p38β to cancer biology has remained largely unexplored. Prior studies have implicated p38β—not p38α—in selective stress-response programs, but its role in TIC maintenance has remained mostly unknown. Our findings fill this gap by demonstrating that p38β, rather than p38α, is the critical isoform governing TIC maintenance and chemoresistance in HCC. p38β uniquely interacts with and phosphorylates BiP at Thr648, enhancing its association with the UPR sensors IRE1α and PERK. This modification reinforces BiP-mediated suppression of UPR activation, preserves ER proteostasis, and enables TICs to survive cytotoxic stress. Functionally, Thr648 phosphorylation is indispensable for p38β-driven stemness and therapeutic resistance, establishing the p38β–BiP axis as a distinct isoform-specific mechanism underlying TIC persistence. Notably, although in vivo genetic rescue of p38β would further strengthen this causal link, the significantly reduced proliferative capacity of p38β-KO cells precluded their efficient expansion for stable re-expression and transplantation in the present study.

BiP serves as a molecular gatekeeper of ER stress by sequestering UPR sensors under homeostatic conditions and releasing them upon stress^11,48,49^. While our mechanistic focus was limited to PERK and IRE1-α due to undetectable ATF6 levels in our system, we cannot exclude its involvement in other contexts. Beyond enhancing BiP binding to PERK and IRE1-α, Thr648 phosphorylation may also modulate BiP conformation or oligomerization dynamics, thereby shifting the activation threshold of UPR sensors^48,50,51^. An important mechanistic consideration is how the cytosolic kinase p38β gains access to BiP, which is classically regarded as an ER-luminal chaperone^5,6^. Increasing evidence suggests that BiP is not exclusively confined to the ER lumen; under stress conditions and during malignant progression, a fraction of BiP has been detected outside the ER, including in cytoplasmic and cell-surface-associated pools^52,53^. Conversely, although p38β is predominantly cytosolic, it can be activated at ER-associated sites, including the cytosolic face of the ER membrane and ER–mitochondria contact regions^54,55^. Consistent with this spatial concordance, our confocal imaging revealed partial overlap between p38β and BiP within the cytoplasmic region, together with detectable proximity of p38β to ER structures. Collectively, these observations support the existence of a cytosolic or ER-associated pool of BiP that is spatially accessible to p38β for phosphorylation at Thr648. Whether this post-translational modification integrates with other cellular stress pathways, such as oxidative stress or mitochondrial dysfunction, remains to be explored. Furthermore, as emerging evidence links UPR signaling to immune evasion and metabolic rewiring in cancer^3,56^, it is conceivable that the p38β-BiP axis intersects with broader tumor-promoting networks beyond ER proteostasis. Although our study employed representative HCC PDX and PDO models, additional clinical validation in larger, more molecularly diverse patient cohorts is warranted. Importantly, while BiP-mediated UPR suppression supports TIC survival under stress, excessive or sustained UPR activation, particularly through PERK or IRE1-α, has been shown to trigger apoptosis in TICs, suggesting a threshold- or duration-dependent role for ER stress in tumor evolution^16,57,58^.

Our results align with previous findings that BiP contributes to stemness maintenance in pancreatic cancer and glioma stem cells^5,59^. We extend this knowledge by demonstrating that its phosphorylation status critically shapes UPR signaling outcomes and thereby modulates TIC-associated stress tolerance. In the current therapeutic era, first-line systemic treatment for advanced HCC is largely based on immune checkpoint inhibitor– and multikinase/RTK inhibitor–containing regimens, such as atezolizumab plus bevacizumab or durvalumab plus tremelimumab, with TKIs including sorafenib and lenvatinib remaining important options in selected patients^60,61^. Although systemic cisplatin monotherapy is no longer a standard first-line approach, cisplatin remains clinically relevant through its central role in conventional and drug-eluting TACE, cisplatin-based HAIC protocols, and emerging sequential or combination strategies with TKIs and ICIs in unresectable or refractory disease^62,63^. Notably, cisplatin, multikinase inhibitors, and immune-mediated cytotoxicity can all impose substantial ER stress on tumor cells, and ER stress/UPR signaling is increasingly recognized as a determinant of HCC progression and therapeutic resistance^40,64,65^. Our data indicate that the p38β–BiP axis functions as a core ER stress–adaptive module that enables TICs to withstand proteotoxic pressure. Thus, beyond explaining cisplatin tolerance, this pathway may represent a broader TIC survival program engaged across multiple treatment modalities in the current ICI/TKI-based treatment landscape.

Given this broader role of the p38β–BiP axis in buffering ER stress across diverse therapeutic pressures, we next assessed whether pharmacological disruption of BiP could translate into tangible therapeutic benefit. Therapeutically, pharmacological inhibition of BiP using HA15 restored UPR activity, increased unfolded protein burden, and re-sensitized p38β-high HCC cells to CDDP. In clinically relevant PDX and PDO models, HA15 synergized with chemotherapy to suppress tumor growth, reduce TIC frequency, and enhance apoptosis. Although various strategies have been developed to target the UPR in cancer, few have specifically addressed TIC-enriched populations^66,67^. The BiP inhibitor HA15 has demonstrated preclinical efficacy, but its impact on stem-like tumor compartments remains unclear^68^. Our findings provide the first in vivo evidence that pharmacological inhibition of BiP disrupts TIC maintenance, particularly in tumors with elevated p38β activity. Given the druggable nature of p38β’s kinase domain, dual targeting of BiP and p38β may offer a synergistic approach to enhance therapeutic efficacy and overcome resistance.

Together, these findings position the p38β-BiP axis as a central stress-adaptive mechanism that links kinase signaling to stemness and drug resistance. This work addresses a key gap in TIC biology and opens new avenues for targeting stress-buffering pathways in refractory HCC. Future studies should explore the therapeutic window of p38β-BiP axis inhibition across tumor types enriched for TICs, such as colorectal, breast, or brain cancers, and investigate potential interactions with immune-based therapies targeting ER stress surveillance.

## Methods

All experiments were conducted in compliance with institutional and national ethical standards. Tissue microarray (TMA) was obtained from Professor Jing-Ping Yun at the Sun Yat-sen University Cancer Center in Guangzhou, China, with the approval of the Institutional Review Board for ethical review from the Sun Yat-sen University in Guangzhou, China, with written informed consent from all patients and no financial compensation provided. All animal experiments were approved by and conducted in accordance with the Hong Kong Animals (Control of Experiments) Ordinance, the Committee on the Use of Live Animals in Teaching and Research at The University of Hong Kong (Approval No. 4012-16), and the Animal Experimentation Ethics Committee of The Chinese University of Hong Kong (Approval No. 22-195-GRF). In accordance with the approved ethical guidelines, the humane endpoint was set at a maximal tumor diameter of 2 cm. Mice that reach or exceed this endpoint are immediately euthanized upon detection.

### Cell lines, PDOs and PDX

Human cell lines HepG2 (HB-8065), Hep3B (HB-8064) and HEK293T (CRL-3216) were obtained from the American Type Culture Collection (ATCC). HCC cell lines HuH-1 and Huh-7 were obtained from the Japanese Cancer Research Resources Bank (JCRB). HCC cell lines MHCC97L and MHCC97H, as well as the immortalized normal liver cell line MIHA, were generously provided by the Liver Cancer Institute of Fudan University. Normal liver cell line THLE-3 was purchased from ATCC. HCC cell line PLC8024 was obtained from the Institute of Virology, Chinese Academy of Medical Sciences (Beijing, China). All cell lines were cultured in Dulbecco’s Modified Eagle Medium (DMEM; Gibco, USA), except for HepG2, which was maintained in Eagle’s Minimum Essential Medium (EMEM; Gibco, USA). Media were supplemented with 10% fetal bovine serum (FBS; Gibco, USA) and 1% penicillin–streptomycin (Gibco, USA). Cells were incubated at 37 °C in a humidified atmosphere containing 5% CO₂. Mycoplasma contamination was monitored every three months, and all cell lines were authenticated by short tandem repeat (STR) profiling.

The two HCC PDOs used in this study are kind gifts from Dr. Meritxell Huch (Max Planck Institute). The culture conditions are as previously described^69^. Normal liver hepatocytes purchased from Thermo Fisher were cultured into normal liver organoids with the same culture conditions as HCC PDOs.

The PDX used in this study^70,71^ was established from resected primary HCC specimens obtained with informed patient consent and approval from the Institutional Review Board of the University of Hong Kong/Hospital Authority Hong Kong West Cluster. Clinicopathological characteristics of the PDX donor patients are summarized in Supplementary Data 4.

### Reagent or resource

A complete list of reagents and resources used in this study is provided in Supplementary Data 5.

### Cell transfection and infection

Wild-type (WT) and phosphorylation-defective mutant (Mut) forms of human p38β were cloned into the pEZ-Lv199 expression vector (GeneCopoeia). Human BiP wild-type (WT), phosphorylation-deficient (T648A, TA), and phosphomimetic (T648D, TD) mutants were cloned into the pLenti-CMV-Blast DEST vector (Addgene, Cat#17451). Cells were cultured in Opti-MEM medium (Invitrogen) and transfected with 2μg of plasmid DNA using Lipofectamine 3000 (Invitrogen) according to the manufacturer’s protocol.

SiRNAs were procured from GenePharma (Shanghai, China) and transfected using Lipofectamine 3000 reagent (Invitrogen) according to the manufacturer’s protocol. The siRNA sequences are listed in Supplementary Data 6.

For stable BiP knockdown, shRNA constructs targeting HSPA5 (shBiP-1: CCGGAGATTCAGCAACTGGTTAAAGCTCGAGCTTTAACCAGTTGC; shBiP-2: CCGGGAAATCGAAAGGATGGTTAATCTCGAGATTAACCATCCTT) were cloned into the pLKO.1 vector. A scrambled shRNA was used as a non-targeting control (shNTC). Lentiviral transduction was performed as previously described^72^.

For CRISPR/Cas9-mediated knockout of p38β, sgRNAs (sgp38β-1: GTCCAGAAGCCCGATGACCT and sgp38β-2: GTACTGGCTGAAGTAGGCGT) were cloned into the pCR-Blunt II-TOPO vector (Addgene, Cat#41824). Cells were co-transfected with plasmids encoding the sgRNAs, human Cas9 (Addgene, Cat#41815), and GFP using Lipofectamine 3000. After 72 hours, GFP-positive cells were isolated via flow cytometry (BD FACSAria I), and single-cell clones were expanded. Knockout efficiency was confirmed by Western blot analysis of p38β protein levels.

### Foci formation assay

1 × 10^³ cells were plated in six-well plates and cultured with medium changed every 3 days. After ~2 weeks, colonies were fixed with 4% paraformaldehyde, stained with 0.1% crystal violet, and quantified using ImageJ.

### In vitro limiting dilution tumorsphere assay

Cells were seeded at densities of 100, 50, 25, 10, or 5 cells (or specified otherwise) per 100 µL in PolyHEMA-coated 96-well plates using DMEM medium supplemented with 0.25% methylcellulose, insulin (4 μg/mL), B27 (1X), EGF (15 ng/mL), and FGF (20 ng/mL). Sphere formation was monitored for 10–14 days, and wells containing spheres ( > 50 μm in diameter) were scored as positive. All sphere counting was performed by an investigator blinded to the experimental group assignments. Tumor-initiating cell frequencies were quantified using Extreme Limiting Dilution Analysis (ELDA; https://bioinf.wehi.edu.au/software/elda/).

### Apoptosis assay

Cells were treated with CDDP for 48 h, then stained with Annexin V–FITC and propidium iodide for 30 min on ice. Apoptosis was assessed by flow cytometry (BD LSRFortessa), and data were analyzed using FlowJo v10.

### Viability assay

XTT assay: Cell viability was determined using the CytoDet Cell Viability Assay Kit (XTT) following the manufacturer’s instructions. Briefly, 4 × 10^³ cells were seeded in 96-well plates (100 μl medium per well) and cultured for 24, 48, 72, and 96 h. The XTT working solution was freshly prepared by mixing the XTT Reagent with the Electron Coupling Reagent at a 100:1 ratio, and 50 μl of the mixture was added to each well. After incubation at 37 °C for 2 h, the absorbance was measured at 450 nm using a microplate reader (Thermo Scientific Multiskan GO).

HCC PDOs treated with HA15 and CDDP for 72 h were subjected to cell viability measurement using the CellTiter-Glo 2.0 Assay (Promega), following the manufacturer’s instructions.

### Immunoprecipitation (IP)

For immunoprecipitation^73^, cells were lysed on ice for 30 min in ice-cold buffer containing protease and phosphatase inhibitors. The clarified lysates were incubated overnight at 4 °C with the indicated primary antibodies (1:100) or the corresponding species-matched IgG (used as a negative control), followed by incubation with protein A/G agarose beads (50 μl per sample) for 2 h at 4 °C with rotation. After washing three times with ice-cold lysis buffer, immune complexes were eluted by boiling in SDS sample buffer and analyzed by SDS-PAGE and immunoblotting.

### Phosphate-affinity (Phos-tag) SDS-PAGE

Phos-tag acrylamide SDS-PAGE was performed using 50 μM Phos-tag acrylamide and 1 mM MnCl₂ to resolve phosphorylated isoforms by reduced electrophoretic mobility. After electrophoresis, gels were destained in 10 mM EDTA to remove Mn²⁺ prior to transfer, and proteins were detected by immunoblotting with total BiP antibody.

### In vitro kinase assay

Recombinant His-tagged p38β and BiP proteins were incubated in kinase reaction buffer containing 25 mM Tris-HCl (pH 7.5) or HEPES (pH 7.5), 10 mM MgCl₂, 1 mM DTT, 50 mM NaCl, 100 μM ATP, 0.01% Tween-20, and 5% glycerol. The reactions were assembled using 50 nM p38β and 250 nM BiP. Kinase activity was initiated by adding a 2× ATP/MgCl₂ mix to achieve final concentrations of 10 mM MgCl₂ and 100 μM ATP. The mixtures were incubated at room temperature for 30 min, after which the reactions were terminated by adding 6×SDS loading buffer followed by heat denaturation at 95 °C for 5 min. The reaction products were resolved by SDS–PAGE and analyzed by immunoblotting.

### In vitro binding assay

Purified recombinant His–p38β and BiP proteins were used to examine their direct physical interaction. For the binding reaction, 1 µg of His–p38β was incubated with 2 µg of BiP in 500 µL binding buffer (20 mM Tris-HCl, pH 7.5, 150 mM NaCl, 1 mM DTT, 0.1% NP-40, 5% glycerol) for 2 h at 4 °C with gentle rotation. The reaction mixture was then incubated with anti-p38β antibody for 4 h at 4 °C, followed by the addition of Protein A/G agarose for an additional 4 h under the same conditions. Beads were washed five times with binding buffer to remove nonspecific proteins, and bound complexes were eluted by boiling in 6×SDS loading buffer, separated by SDS–PAGE, and analyzed by immunoblotting with anti-His and anti-BiP antibodies.

### Tandem affinity purification followed by mass spectrometry (TAP-MS)

TAP-MS was performed as previously described to identify p38β-interacting proteins^74^. Briefly, HEK293T cells were transfected with SFB-tagged p38β construct and lysed under non-denaturing conditions. Tandem affinity purification was carried out using streptavidin and S-protein beads. Eluted proteins were loaded on SDS-PAGE and stained with Coomassie Blue. The gel slices containing the eluted proteins were subjected to LC-MS/MS at the Taplin Mass Spectrometry Facility (Harvard Medical School). Potential binding proteins of p38β were distinguished from non-specific interacting proteins on the beads by cross-comparison against the list of common contaminant proteins derived from our previous studies^74,75^. The prioritized candidate p38β-associated proteins are listed in Supplementary Data 2. MS data are deposited in PRIDE: PXD067039.

### Phosphoproteomic analysis by LC–MS/MS

MHCC97L cells co-expressing SFB-tagged BiP with p38β WT, kinase-dead p38β Mut, or empty-vector (EV) controls, were immunoprecipitated with streptavidin beads for SFB-tagged BiP pull-down. SFB-tagged BiP immunoprecipitates were subjected to SDS-PAGE and Coomassie Blue staining, using a single biological replicate per condition. The gel lanes were separately cut into slices and subjected to in-gel digestion. The tryptic peptides were subjected to phosphoproteomic profiling by LC-MS/MS. LC–MS/MS analysis was conducted at the Proteomics and Metabolomics Core Facility (PMCore), Center for PanorOmic Sciences (CPOS), LKS Faculty of Medicine, The University of Hong Kong. Peptides were separated on a Dionex Ultimate 3000 nanoRSLC system (Thermo Fisher Scientific) coupled to an Orbitrap Fusion Tribrid Lumos mass spectrometer (Thermo Fisher Scientific). Raw mass spectrometry data were processed using MaxQuant 1.6.0.13. Raw data was searched against Human UniProt FASTA database (April 2020) containing 31, 165 entries, and also targeted HSPA5/BiP targeted full length protein sequence, using settings as below: oxidized methionine (M), acetylation (Protein N-term) were selected as dynamic modifications, and Phospho STY, carbamidomethyl (C) as fixed modifications with minimum peptide length of seven amino acids was enabled. Confident proteins were identified using a target-decoy approach with a reversed database, strict false-discovery rate 1% at peptide and peptide spectrum matches (PSMs) level; minimum ≥ 2 unique peptides, ≥2 peptide spectral matches (PSMs), localization probability score > 0.75, Delta score > 80. All identified BiP phosphorylation sites and associated quantitative values are provided in Supplementary Data 3, and the raw MS/MS data have been deposited in the PRIDE repository (accession PXD067039).

### Immunohistochemistry (IHC)

IHC staining was performed using the following procedure. Antigen retrieval was performed using citrate buffer. Primary antibody binding was detected with the Dako EnVision HRP Detection System and visualized using DAB substrate, following the manufacturer’s protocols. Quantitative analysis of staining intensity was performed using ImageJ software. Three mice per group were randomly selected for IHC staining and quantification. Field selection and scoring were performed in a blinded manner by two independent investigators to minimize bias. For each tumor sample, multiple representative fields (at least five fields per section) were captured, and the percentage of positive cells or staining intensity was averaged to obtain a per-animal mean. The same image-analysis pipeline and quantification criteria were applied consistently across all markers.

### Unfolded protein quantification

Intracellular levels of unfolded proteins were assessed using a thiol-reactive fluorescent probe (TPE-MI) generously provided by Dr. Yuning Hong (La Trobe University, Australia), as previously described^35^. Cells were incubated with the probe under recommended conditions, followed by washing to remove excess dye. Fluorescence intensity reflecting unfolded protein accumulation was measured by flow cytometry using a BD FACSAria II instrument (BD Biosciences). Flow cytometry data were processed and analyzed with FlowJo software (Tree Star) to quantify relative unfolded protein levels across experimental groups.

### Pharmacological treatment and intratumoral AAV8 delivery in HCC PDX

Male NOD/SCID mice aged 6 weeks bearing PDX tumors were randomized to receive HA15 (10 mg/kg, intraperitoneally [*i.p*.], every 2 days for 2 weeks) and CDDP (5 mg/kg, *i.p*., every 2 days for 2 weeks). AAV8 vectors (AAV8-CMV-MCS-eGFP) expressing luciferase reporter (EV), p38β WT or kinase-dead p38β Mut were produced by GenePharma (Shanghai, China). Established HCC PDX tumors were randomized and injected intratumorally with AAV8-EV, AAV8-p38β WT or AAV8-p38β Mut. For intratumoral delivery, each tumor was injected at three evenly spaced sites around the tumor circumference (approximately one-third of the perimeter apart), with injections performed sequentially to ensure uniform distribution. Tumor measurements and endpoint assessments were conducted in a non-blinded manner.

### In vivo limiting dilution transplantation assay

For xenograft establishment, HCC cells were resuspended in Matrigel (Corning, Cat#354234) at a 1:1 ratio and injected subcutaneously into the flanks of 6-week-old male NOD/SCID mice at graded cell doses (10,000, 5000, 2000 and 1000 cells). Tumor formation was monitored over a period of 2–3 months, and tumor incidence, volume, and tumor-free survival were recorded. For secondary transplantation, tumors derived from primary recipients were excised, dissociated into single-cell suspensions, and re-implanted into secondary NOD/SCID mice at limiting dilutions using the same procedure.

Tumor-initiating cell (TIC) frequency was calculated using extreme limiting dilution analysis (ELDA) (https://bioinf.wehi.edu.au/software/elda/). Tumor volumes were measured using calipers and calculated as (length × width²)/2. At experimental end point, mice were euthanized, and tumors were harvested, weighed, and processed for downstream analysis. Tumor tissues were fixed in formalin, embedded in paraffin, and subjected to hematoxylin and eosin (H&E) staining. IHC staining was performed to evaluate the expression of p-IRE1-α, proliferating cell nuclear antigen (PCNA), and TUNEL positivity.

### HCC patient stratification for clinicopathological analyses

In-house HCC tumor samples from tissue microarray were classified according to p38β expression relative to their matched adjacent non-tumor tissue. Staining intensity was quantified using ImageJ software. Cases in which the tumor H-score exceeded that of the corresponding non-tumor tissue were designated as “p38β-high”, whereas those with lower tumor H-scores were defined as “p38β-low”. Samples with equivalent H-scores between tumor and adjacent tissue were categorized as “no difference”.

Quantification of site-specific BiP phosphorylation at Thr648 in the HCC phosphoproteomic cohort (PDC000199) was based on the phosphopeptide LYGSAGPPPTGEEDtAEKDEL (t = pThr) with log_2_-normalized MS1 intensity values. Cases were stratified according to phosphorylated BiP (Thr648) levels, with values ≥ 0 defined as the high group (*n* = 41) and values < 0 as the low group (*n* = 60).

### Statistics and reproducibility

Quantitative data are expressed as the mean ± standard deviation (SD). Statistical analyses were performed using GraphPad Prism (version 10.1.2) and SPSS software (version 21.0). An unpaired two-tailed Student’s t-test was used for comparing two groups, while one- or two-way ANOVA with Tukey’s test was used for multiple groups. Kaplan–Meier survival curves were compared using two-sided log-rank tests. *P* value less than 0.05 was considered statistically significant. Sample sizes were determined based on prior experiments and published studies, not by formal statistical calculations. Unless otherwise indicated, experiments were independently repeated at least three times with similar results, including experiments for which representative images are shown. Details of sample sizes are provided in the figure legends. All data were included in the analysis.

### Reporting summary

Further information on research design is available in the Nature Portfolio Reporting Summary linked to this article.

## Supplementary information

## Source data

## Data Availability

The transcriptomic dataset of chemotherapy-enriched HCC spheroids used in this study is publicly available in the Gene Expression Omnibus (GEO) database under accession code GSE53005. Transcriptomic levels of MAPK11 and stemness marker genes are available in The Cancer Genome Atlas Liver Hepatocellular Carcinoma (TCGA-LIHC). Site-specific BiP phosphorylation levels in HCC patients are available in Clinical Proteomic Tumor Analysis Consortium (CPTAC, PDC000199) (https://proteomic.datacommons.cancer.gov/pdc/study/PDC000199). All mass spectrometry datasets generated in this study, including phosphoproteomic and other proteomic analyses, have been deposited in the PRIDE repository under accession code PXD067039. All other data supporting the findings of this study, including quantitative results from functional assays, are available within the Source Data file. Source data are provided with this paper.

## References

[1] Ladd, A. D., Duarte, S., Sahin, I. & Zarrinpar, A. Mechanisms of drug resistance in HCC. *Hepatology* **79**, 926–940 (2024). 36680397 10.1097/HEP.0000000000000237

[2] Kudo, M. et al. Lenvatinib versus sorafenib in first-line treatment of patients with unresectable hepatocellular carcinoma: a randomised phase 3 non-inferiority trial. *Lancet* **391**, 1163–1173 (2018). 29433850 10.1016/S0140-6736(18)30207-1

[3] Cubillos-Ruiz, J. R., Bettigole, S. E. & Glimcher, L. H. Tumorigenic and immunosuppressive effects of endoplasmic reticulum stress in cancer. *Cell* **168**, 692–706 (2017). 28187289 10.1016/j.cell.2016.12.004 PMC5333759

[4] Scheller-Wendorff, M. & Muller-Tidow, C. IRE1alpha maintains HSC stemness under ER-stress. *Nat. Cell Biol.* **21**, 297–298 (2019). 30778221 10.1038/s41556-019-0295-4

[5] Dauer, P. et al. ER stress sensor, glucose regulatory protein 78 (GRP78) regulates redox status in pancreatic cancer thereby maintaining “stemness. *Cell Death Dis.* **10**, 132 (2019). 30755605 10.1038/s41419-019-1408-5 PMC6372649

[6] Chen, X. & Cubillos-Ruiz, J. R. Endoplasmic reticulum stress signals in the tumour and its microenvironment. *Nat. Rev. Cancer* **21**, 71–88 (2021). 33214692 10.1038/s41568-020-00312-2 PMC7927882

[7] Ron, D. & Walter, P. Signal integration in the endoplasmic reticulum unfolded protein response. *Nat. Rev. Mol. Cell Biol.* **8**, 519–529 (2007). 17565364 10.1038/nrm2199

[8] Hetz, C., Chevet, E. & Harding, H. P. Targeting the unfolded protein response in disease. *Nat. Rev. Drug Discov.* **12**, 703–719 (2013). 23989796 10.1038/nrd3976

[9] Walter, P. & Ron, D. The unfolded protein response: from stress pathway to homeostatic regulation. *Science* **334**, 1081–1086 (2011). 22116877 10.1126/science.1209038

[10] Wiseman, R. L., Mesgarzadeh, J. S. & Hendershot, L. M. Reshaping endoplasmic reticulum quality control through the unfolded protein response. *Mol. Cell* **82**, 1477–1491 (2022). 35452616 10.1016/j.molcel.2022.03.025 PMC9038009

[11] Bertolotti, A., Zhang, Y., Hendershot, L. M., Harding, H. P. & Ron, D. Dynamic interaction of BiP and ER stress transducers in the unfolded-protein response. *Nat. Cell Biol.* **2**, 326–332 (2000). 10854322 10.1038/35014014

[12] Harding, H. P. et al. Regulated translation initiation controls stress-induced gene expression in mammalian cells. *Mol. Cell* **6**, 1099–1108 (2000). 11106749 10.1016/s1097-2765(00)00108-8

[13] Calfon, M. et al. IRE1 couples endoplasmic reticulum load to secretory capacity by processing the XBP-1 mRNA. *Nature* **415**, 92–96 (2002). 11780124 10.1038/415092a

[14] Lee, A. H., Scapa, E. F., Cohen, D. E. & Glimcher, L. H. Regulation of hepatic lipogenesis by the transcription factor XBP1. *Science* **320**, 1492–1496 (2008). 18556558 10.1126/science.1158042 PMC3620093

[15] Shen, J., Chen, X., Hendershot, L. & Prywes, R. ER stress regulation of ATF6 localization by dissociation of BiP/GRP78 binding and unmasking of Golgi localization signals. *Dev. Cell* **3**, 99–111 (2002). 12110171 10.1016/s1534-5807(02)00203-4

[16] Hetz, C. & Papa, F. R. The unfolded protein response and cell fate control. *Mol. Cell* **69**, 169–181 (2018). 29107536 10.1016/j.molcel.2017.06.017

[17] Hetz, C., Axten, J. M. & Patterson, J. B. Pharmacological targeting of the unfolded protein response for disease intervention. *Nat. Chem. Biol.* **15**, 764–775 (2019). 31320759 10.1038/s41589-019-0326-2

[18] Liang, D., Khoonkari, M., Avril, T., Chevet, E. & Kruyt, F. A. E. The unfolded protein response as regulator of cancer stemness and differentiation: mechanisms and implications for cancer therapy. *Biochem Pharm.* **192**, 114737 (2021). 34411568 10.1016/j.bcp.2021.114737

[19] Iqbal, M. A. et al. The integrated stress response promotes neural stem cell survival under conditions of mitochondrial dysfunction in neurodegeneration. *Aging Cell* **23**, e14165 (2024). 38757355 10.1111/acel.14165 PMC11258489

[20] Han, J., Lee, J. D., Bibbs, L. & Ulevitch, R. J. A MAP kinase targeted by endotoxin and hyperosmolarity in mammalian cells. *Science* **265**, 808–811 (1994). 7914033 10.1126/science.7914033

[21] Wagner, E. F. & Nebreda, A. R. Signal integration by JNK and p38 MAPK pathways in cancer development. *Nat. Rev. Cancer* **9**, 537–549 (2009). 19629069 10.1038/nrc2694

[22] Martinez-Limon, A., Joaquin, M., Caballero, M., Posas, F. & de Nadal, E. The p38 Pathway: from Biology to Cancer Therapy. *Int J. Mol. Sci.* **21**, 1913 (2020). 32168915 10.3390/ijms21061913 PMC7139330

[23] Dolado, I. et al. p38alpha MAP kinase as a sensor of reactive oxygen species in tumorigenesis. *Cancer Cell* **11**, 191–205 (2007). 17292829 10.1016/j.ccr.2006.12.013

[24] Beenstock, J. et al. The p38beta mitogen-activated protein kinase possesses an intrinsic autophosphorylation activity, generated by a short region composed of the alpha-G helix and MAPK insert. *J. Biol. Chem.* **289**, 23546–23556 (2014). 25006254 10.1074/jbc.M114.578237 PMC4156038

[25] Roche, O. et al. p38beta and cancer: the beginning of the road. *Int J. Mol. Sci.* **21**, 7524 (2020). 33053909 10.3390/ijms21207524 PMC7589630

[26] Liu, S. et al. Mammalian IRE1alpha dynamically and functionally coalesces with stress granules. *Nat. Cell Biol.* **26**, 917–931 (2024). 38714852 10.1038/s41556-024-01418-7

[27] Lee, T. K. et al. Blockade of CD47-mediated cathepsin S/protease-activated receptor 2 signaling provides a therapeutic target for hepatocellular carcinoma. *Hepatology* **60**, 179–191 (2014). 24523067 10.1002/hep.27070

[28] Yovchev, M. I., Grozdanov, P. N., Joseph, B., Gupta, S. & Dabeva, M. D. Novel hepatic progenitor cell surface markers in the adult rat liver. *Hepatology* **45**, 139–149 (2007). 17187413 10.1002/hep.21448

[29] Zhang, K. et al. In vitro expansion of primary human hepatocytes with efficient liver repopulation capacity. *Cell Stem Cell* **23**, 806–819 e804 (2018). 30416071 10.1016/j.stem.2018.10.018

[30] Borisova, M. E. et al. p38-MK2 signaling axis regulates RNA metabolism after UV-light-induced DNA damage. *Nat. Commun.* **9**, 1017 (2018). 10.1038/s41467-018-03417-3 PMC5845016 29523821

[31] Lin, M., Mo, Y., Li, C. M., Liu, Y. Z. & Feng, X. P. GRP78 as a potential therapeutic target in cancer treatment: an updated review of its role in chemoradiotherapy resistance of cancer cells. *Med. Oncol.* **42**, 49 (2025). 39827214 10.1007/s12032-024-02586-0

[32] Cohen, P. The origins of protein phosphorylation. *Nat. Cell Biol.* **4**, E127–E130 (2002). 11988757 10.1038/ncb0502-e127

[33] Wei, Y. et al. EGFR-mediated Beclin 1 phosphorylation in autophagy suppression, tumor progression, and tumor chemoresistance. *Cell* **154**, 1269–1284 (2013). 24034250 10.1016/j.cell.2013.08.015 PMC3917713

[34] Wang, J. et al. A novel pathway for stemness propagation and chemoresistance in non-small cell lung cancer via phosphorylated PKM2-loaded small extracellular vesicles. *Theranostics* **15**, 3439–3461 (2025). 40093893 10.7150/thno.103722 PMC11905138

[35] Chen, M. Z. et al. A thiol probe for measuring unfolded protein load and proteostasis in cells. *Nat. Commun.* **8**, 474 (2017). 28883394 10.1038/s41467-017-00203-5 PMC5589734

[36] Ianevski, A., Giri, A. K. & Aittokallio, T. SynergyFinder 3.0: an interactive analysis and consensus interpretation of multi-drug synergies across multiple samples. *Nucleic Acids Res.* **50**, W739–W743 (2022). 35580060 10.1093/nar/gkac382 PMC9252834

[37] Li, N. & Zhu, Y. Targeting liver cancer stem cells for the treatment of hepatocellular carcinoma. *Ther. Adv. Gastroenterol.* **12**, 1756284818821560 (2019). 30719075 10.1177/1756284818821560 PMC6348509

[38] Yang, C. et al. Evolving therapeutic landscape of advanced hepatocellular carcinoma. *Nat. Rev. Gastroenterol. Hepatol.* **20**, 203–222 (2023). 36369487 10.1038/s41575-022-00704-9

[39] Zuo, W. F. et al. Heat shock proteins as hallmarks of cancer: insights from molecular mechanisms to therapeutic strategies. *J. Hematol. Oncol.* **17**, 81 (2024). 39232809 10.1186/s13045-024-01601-1 PMC11375894

[40] Urra, H., Dufey, E., Avril, T., Chevet, E. & Hetz, C. Endoplasmic reticulum stress and the hallmarks of cancer. *Trends Cancer* **2**, 252–262 (2016). 28741511 10.1016/j.trecan.2016.03.007

[41] Igea, A. & Nebreda, A. R. The stress kinase p38alpha as a target for cancer therapy. *Cancer Res.* **75**, 3997–4002 (2015). 26377941 10.1158/0008-5472.CAN-15-0173

[42] Zheng, F. et al. p38alpha MAPK-mediated induction and interaction of FOXO3a and p53 contribute to the inhibited-growth and induced-apoptosis of human lung adenocarcinoma cells by berberine. *J. Exp. Clin. Cancer Res* **33**, 36 (2014). 24766860 10.1186/1756-9966-33-36 PMC4013801

[43] Slobodnyuk, K. et al. Autophagy-induced senescence is regulated by p38alpha signaling. *Cell Death Dis.* **10**, 376 (2019). 31092814 10.1038/s41419-019-1607-0 PMC6520338

[44] Canovas, B. et al. Targeting p38alpha increases DNA damage, chromosome instability, and the anti-tumoral response to taxanes in breast cancer cells. *Cancer Cell* **33**, 1094–1110 e1098 (2018). 29805078 10.1016/j.ccell.2018.04.010

[45] Hui, L. et al. p38alpha suppresses normal and cancer cell proliferation by antagonizing the JNK-c-Jun pathway. *Nat. Genet* **39**, 741–749 (2007). 17468757 10.1038/ng2033

[46] Yuan, W. et al. Modulating p38 MAPK signaling by proteostasis mechanisms supports tissue integrity during growth and aging. *Nat. Commun.* **14**, 4543 (2023). 37507441 10.1038/s41467-023-40317-7 PMC10382525

[47] Yuan, Q. et al. p38 mediated ACSL4 phosphorylation drives stress-induced esophageal squamous cell carcinoma growth through Src myristoylation. *Nat. Commun.* **16**, 3319 (2025). 40195298 10.1038/s41467-025-58342-z PMC11976994

[48] Carrara, M., Prischi, F., Nowak, P. R., Kopp, M. C. & Ali, M. M. Noncanonical binding of BiP ATPase domain to Ire1 and Perk is dissociated by unfolded protein CH1 to initiate ER stress signaling. *Elife* **4**, 03522 (2015). 10.7554/eLife.03522 PMC4337721 25692299

[49] Kimata, Y., Oikawa, D., Shimizu, Y., Ishiwata-Kimata, Y. & Kohno, K. A role for BiP as an adjustor for the endoplasmic reticulum stress-sensing protein Ire1. *J. Cell Biol.* **167**, 445–456 (2004). 15520230 10.1083/jcb.200405153 PMC2172501

[50] Kopp, M. C., Larburu, N., Durairaj, V., Adams, C. J. & Ali, M. M. U. UPR proteins IRE1 and PERK switch BiP from chaperone to ER stress sensor. *Nat. Struct. Mol. Biol.* **26**, 1053–1062 (2019). 31695187 10.1038/s41594-019-0324-9 PMC6858872

[51] Pincus, D. et al. BiP binding to the ER-stress sensor Ire1 tunes the homeostatic behavior of the unfolded protein response. *PLoS Biol.* **8**, e1000415 (2010). 20625545 10.1371/journal.pbio.1000415 PMC2897766

[52] Lee, A. S. Stress-induced translocation of the endoplasmic reticulum chaperone GRP78/BiP and its impact on human disease and therapy. *Proc. Natl. Acad. Sci. USA* **122**, e2412246122 (2025). 40699920 10.1073/pnas.2412246122 PMC12318151

[53] Sicari, D. et al. Reflux of endoplasmic reticulum proteins to the cytosol inactivates tumor suppressors. *EMBO Rep.* **22**, e51412 (2021). 33710763 10.15252/embr.202051412 PMC8724677

[54] Li, L. et al. p38 MAP kinase-dependent phosphorylation of the Gp78 E3 ubiquitin ligase controls ER-mitochondria association and mitochondria motility. *Mol. Biol. Cell* **26**, 3828–3840 (2015). 26337390 10.1091/mbc.E15-02-0120 PMC4626067

[55] Li, W. et al. Phosphorylation of LAMP2A by p38 MAPK couples ER stress to chaperone-mediated autophagy. *Nat. Commun.* **8**, 1763 (2017). 29176575 10.1038/s41467-017-01609-x PMC5701254

[56] Grenier, A. et al. AMPK-PERK axis represses oxidative metabolism and enhances apoptotic priming of mitochondria in acute myeloid leukemia. *Cell Rep.* **38**, 110197 (2022). 34986346 10.1016/j.celrep.2021.110197

[57] Lee, A. S. Glucose-regulated proteins in cancer: molecular mechanisms and therapeutic potential. *Nat. Rev. Cancer* **14**, 263–276 (2014). 24658275 10.1038/nrc3701 PMC4158750

[58] Maly, D. J. & Papa, F. R. Druggable sensors of the unfolded protein response. *Nat. Chem. Biol.* **10**, 892–901 (2014). 25325700 10.1038/nchembio.1664 PMC4664160

[59] Chen, Z. et al. Cell surface GRP78 regulates BACE2 via lysosome-dependent manner to maintain mesenchymal phenotype of glioma stem cells. *J. Exp. Clin. Cancer Res* **40**, 20 (2021). 33413577 10.1186/s13046-020-01807-4 PMC7791784

[60] Gordan, J. D. et al. Systemic therapy for advanced hepatocellular carcinoma: ASCO guideline update. *J. Clin. Oncol.* **42**, 1830–1850 (2024). 38502889 10.1200/JCO.23.02745

[61] Fong, K. Y. et al. First-line systemic therapies for advanced hepatocellular carcinoma: a systematic review and patient-level network meta-analysis. *Liver Cancer* **12**, 7–18 (2023). 36872922 10.1159/000526639 PMC9982345

[62] Hamaya, S., Oura, K., Morishita, A. & Masaki, T. Cisplatin in liver cancer therapy. *Int J. Mol. Sci.* **24**, 10858 (2023). 37446035 10.3390/ijms241310858 PMC10341575

[63] Si, T., Huang, Z., Khorsandi, S. E., Ma, Y. & Heaton, N. Hepatic arterial infusion chemotherapy versus transarterial chemoembolization for unresectable hepatocellular carcinoma: a systematic review with meta-analysis. *Front. Bioeng. Biotechnol.* **10**, 1010824 (2022). 36237208 10.3389/fbioe.2022.1010824 PMC9551027

[64] Mandic, A., Hansson, J., Linder, S. & Shoshan, M. C. Cisplatin induces endoplasmic reticulum stress and nucleus-independent apoptotic signaling. *J. Biol. Chem.* **278**, 9100–9106 (2003). 12509415 10.1074/jbc.M210284200

[65] Shi, Y. H. et al. Targeting autophagy enhances sorafenib lethality for hepatocellular carcinoma via ER stress-related apoptosis. *Autophagy* **7**, 1159–1172 (2011). 21691147 10.4161/auto.7.10.16818

[66] Unal, B. et al. Targeting IRE1alpha reprograms the tumor microenvironment and enhances anti-tumor immunity in prostate cancer. *Nat. Commun.* **15**, 8895 (2024). 39406723 10.1038/s41467-024-53039-1 PMC11480464

[67] Wang, J. et al. XBP1s activates METTL3/METTL14 for ER-phagy and paclitaxel sensitivity regulation in breast cancer. *Cancer Lett.* **596**, 216846 (2024). 38582397 10.1016/j.canlet.2024.216846

[68] Yang, Y. et al. Tumorous IRE1alpha facilitates CD8(+)T cells-dependent anti-tumor immunity and improves immunotherapy efficacy in melanoma. *Cell Commun. Signal* **22**, 83 (2024). 38291473 10.1186/s12964-024-01470-8 PMC10826282

[69] Tong, M. et al. Loss of tyrosine catabolic enzyme HPD promotes glutamine anaplerosis through mTOR signaling in liver cancer. *Cell Rep.* **37**, 109976 (2021). 34731599 10.1016/j.celrep.2021.109976

[70] Tong, M. et al. Efficacy of annexin A3 blockade in sensitizing hepatocellular carcinoma to sorafenib and regorafenib. *J. Hepatol.* **69**, 826–839 (2018). 29885413 10.1016/j.jhep.2018.05.034

[71] Mok, E. H. K. et al. Caspase-3-induced activation of SREBP2 drives drug resistance via promotion of cholesterol biosynthesis in hepatocellular carcinoma. *Cancer Res*. **82**, 3102–3115 (2022). 10.1158/0008-5472.CAN-21-2934 35767704

[72] Xu, L. et al. PHB2 promotes SHIP2 ubiquitination via the E3 ligase NEDD4 to regulate AKT signaling in gastric cancer. *J. Exp. Clin. Cancer Res.* **43**, 17 (2024). 38200519 10.1186/s13046-023-02937-1 PMC10782615

[73] Xu, L. et al. SNORD80-guided 2’-O-methylation stabilizes the lncRNA GAS5 to regulate cellular stress responses. *Proc. Natl. Acad. Sci. USA* **122**, e2418996122 (2025). 39946530 10.1073/pnas.2418996122 PMC11848286

[74] Che, N. et al. PRMT6 deficiency induces autophagy in hostile microenvironments of hepatocellular carcinoma tumors by regulating BAG5-associated HSC70 stability. *Cancer Lett.* **501**, 247–262 (2021). 33186656 10.1016/j.canlet.2020.11.002

[75] Chan, L. H. et al. PRMT6 Regulates RAS/RAF Binding and MEK/ERK-mediated cancer stemness activities in hepatocellular carcinoma through CRAF methylation. *Cell Rep.* **25**, 690–701 e698 (2018). 30332648 10.1016/j.celrep.2018.09.053

