# Supplementary material for: p38β-mediated BiP phosphorylation drives stemness and chemoresistance by suppressing UPR activation in hepatocellular carcinoma
Source: Nat Commun. 2026 Aug 5;17:9415. doi: 10.1038/s41467-026-76073-7 (PMC13538570; doi:10.1038/s41467-026-76073-7)
Supplement: Supplementary file 1 [file 41467_2026_76073_MOESM1_ESM.pdf]

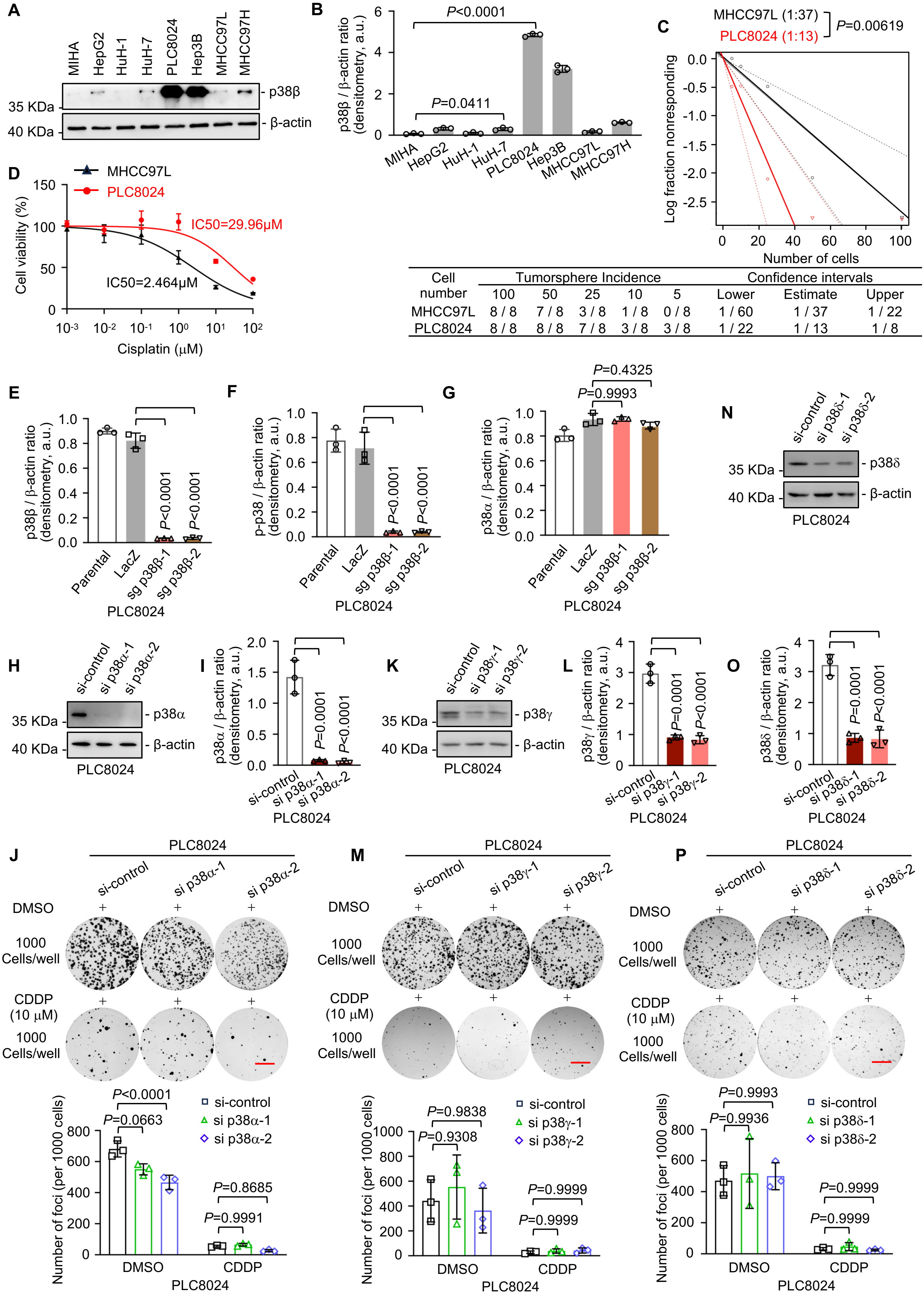

Cell number	Tumorsphere Incidence					Confidence intervals		
	100	50	25	10	5	Lower	Estimate	Upper
MHCC97L	8 / 8	7 / 8	3 / 8	1 / 8	0 / 8	1 / 60	1 / 37	1 / 22
PLC8024	8 / 8	8 / 8	7 / 8	3 / 8	3 / 8	1 / 22	1 / 13	1 / 8

Supplementary Figure 1. p38β is highly expressed in HCC cell lines and shows a non-redundant role among p38 MAPK isoforms in cisplatin response. A, B Western blot analysis of p38β expression in a panel of HCC cell lines and the normal liver cell line MIHA (**A**), with densitometric quantification of p38β normalized to β-actin (**B**). **C** ELDA comparing sphere-forming frequency between MHCC97L and PLC8024 cells. Top, ELDA plot with fitted lines. Bottom, estimated stem-cell frequency (1/x) with 95% confidence intervals and goodness-of-fit statistics. (n = 3 independent experiments). **D** Dose-response curves of MHCC97L and PLC8024 cells treated with increasing concentrations of cisplatin for 72 hours, with cell viability measured by XTT assay. IC_{50} values are indicated. (n = 3 independent experiments). **E-G** Densitometric quantification of the western blot bands shown in Figure 1L, p38β (**E**), p-p38β (**F**) and p38*α* (**G**) levels were normalized to β-actin. **H, K, N** Immunoblot validation of p38α (**H**), p38γ (**K**) and p38δ (**N**) knockdown in PLC8024 cells using two independent siRNAs. For panels D, H, K and N, samples within each experiment derived from the same experiment, but separate gels for the indicated proteins and controls were processed in parallel. **I, L, O** Corresponding densitometric quantification, normalized to β-actin. **J, M, P** Foci formation assays of PLC8024 cells following knockdown of p38α (**J**), p38γ (**M**) or p38δ (**P**) under DMSO or CDDP (10 µM) treatment as indicated (1,000 cells/well). Bottom (**J, M, P**), quantification of foci numbers. Scale bar, 1 cm; n = 3 independent experiments.

Data represent three independent experiments (**A, H, K, N**). Values are mean ± SD (**B, E-G, I, J, L, M, O, P**). Two-sided ELDA likelihood-ratio test (**C**). One-way ANOVA followed by Tukey's multiple comparison (**B, E-G, I, J, L, M, O, P**).

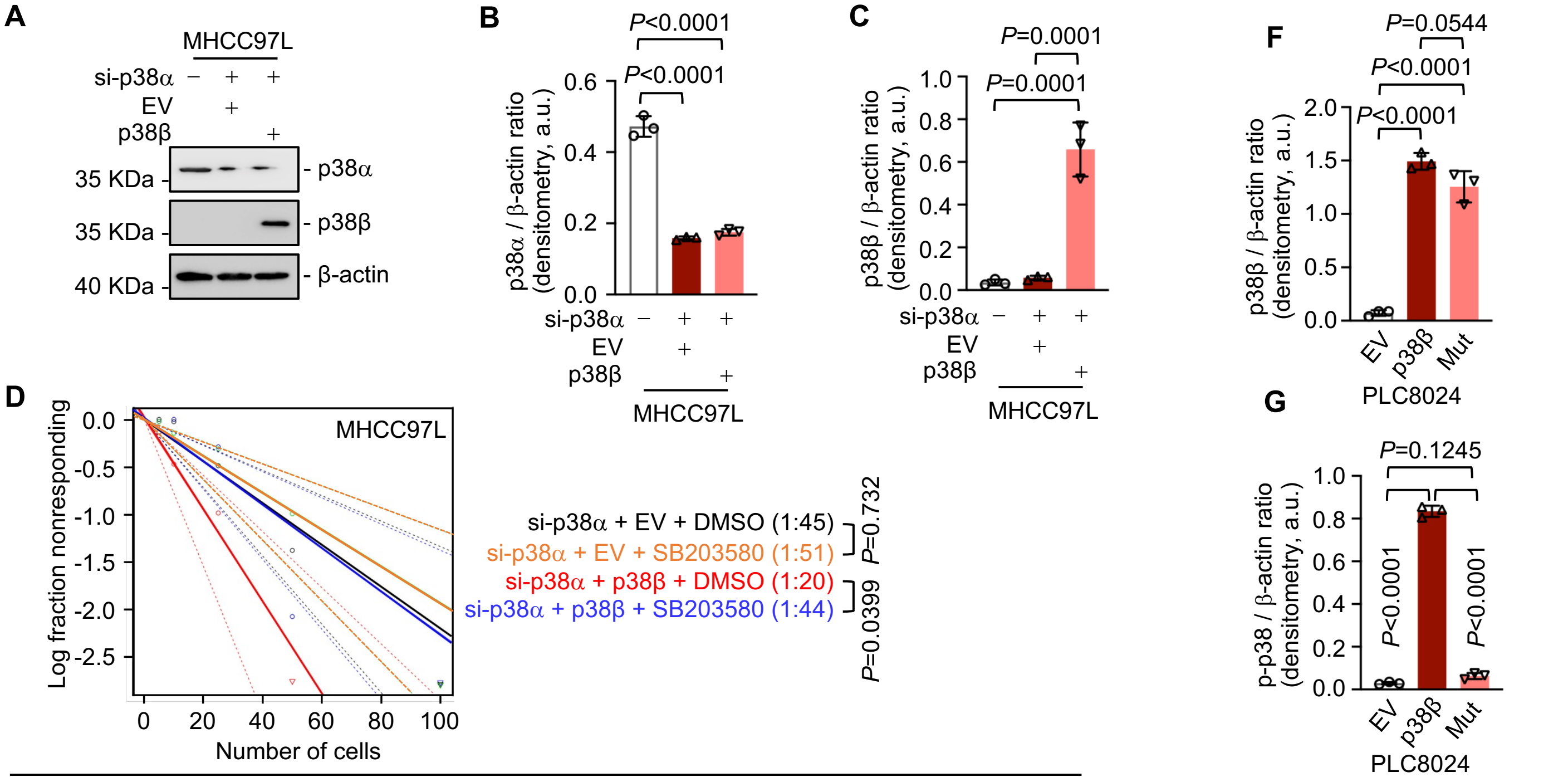

Cell number	Tumorsphere incidence					Confidence intervals		
	100	50	25	10	5	Lower	Estimate	Upper
si-p38α + EV + DMSO	8 / 8	6 / 8	2 / 8	0 / 8	0 / 8	1 / 74	1 / 45	1 / 27
si-p38α + EV + SB203580	8 / 8	5 / 8	2 / 8	1 / 8	0 / 8	1 / 85	1 / 51	1 / 31
si-p38α + p38β + DMSO	8 / 8	8 / 8	5 / 8	3 / 8	1 / 8	1 / 33	1 / 20	1 / 12
si-p38α + p38β + SB203580	8 / 8	7 / 8	2 / 8	0 / 8	0 / 8	1 / 72	1 / 44	1 / 27

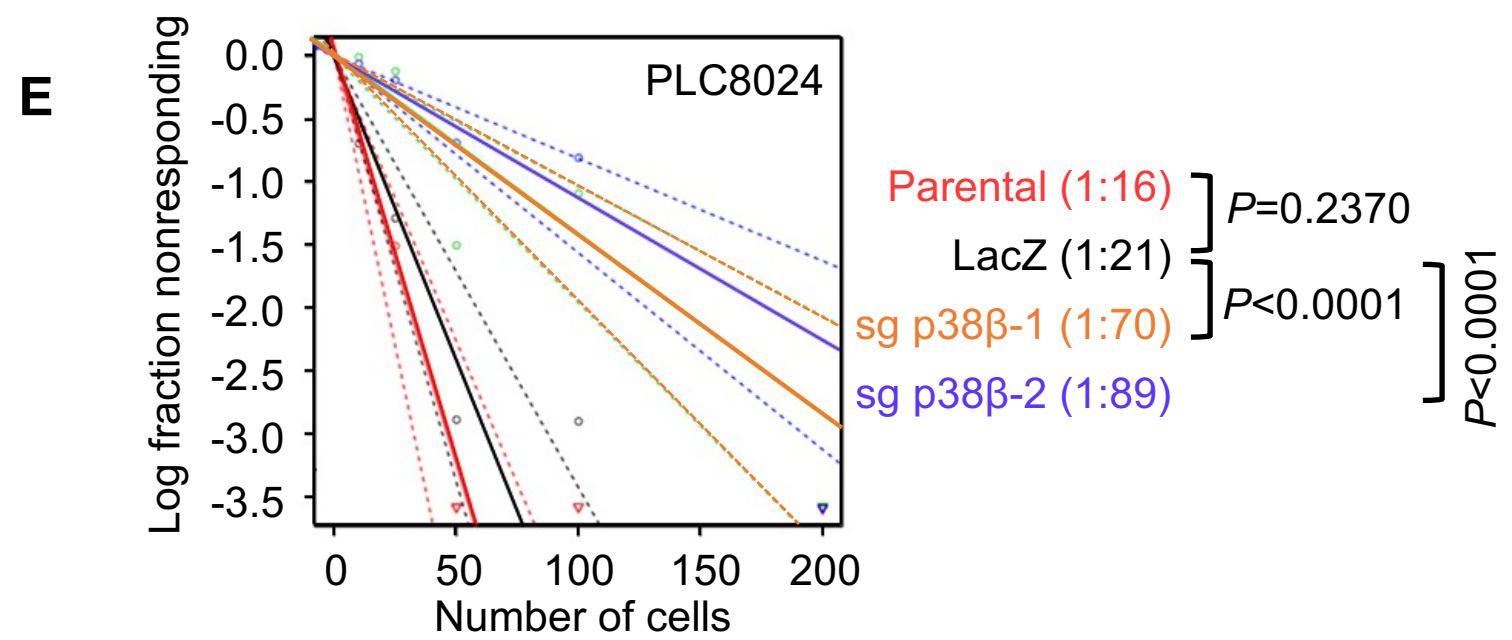

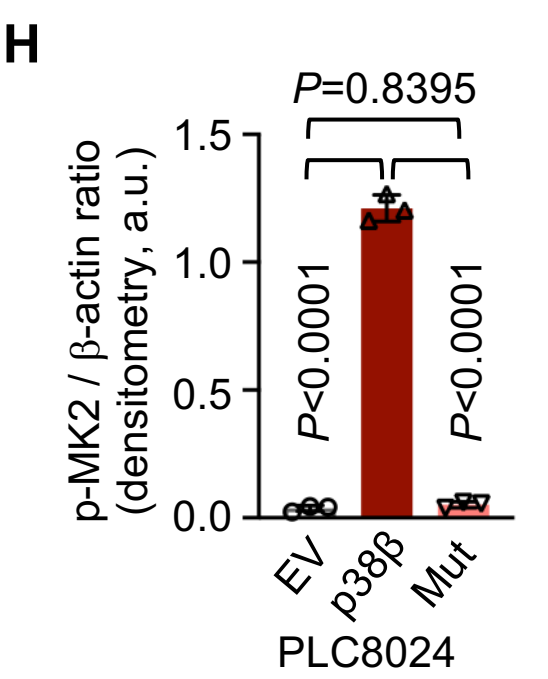

Cell number	Tumorsphere incidence					Confidence intervals			Likelihood-ratio test P-values
	200	100	50	25	10	Lower	Estimate	Upper	
Parental	18 / 18	18 / 18	18 / 18	14 / 18	8 / 18	1 / 22	1 / 16	1 / 11	
LacZ	18 / 18	17 / 18	17 / 18	13 / 18	9 / 18	1 / 29	1 / 21	1 / 15	<0.001
sg p38β-1	18 / 18	12 / 18	14 / 18	2 / 18	0 / 18	1 / 97	1 / 70	1 / 51	
sg p38β-2	18 / 18	10 / 18	9 / 18	3 / 18	1 / 18	1 / 123	1 / 89	1 / 64	

Supplementary Figure 2. Suppression of p38β reduces self-renewal and tumor initiation in HCC cells in a kinase activity-dependent manner. A-C Immunoblot analysis of p38α and p38β expression in MHCC97L cells overexpressing p38β and subjected to p38α knockdown (**A**), with densitometric quantification of p38α (**B**) and p38β (**C**) normalized to β-actin. The samples derive from the same experiment, but separate gels for p38α, p38β and β-actin were processed in parallel. **D** Limiting dilution tumorsphere formation assays of MHCC97L cells overexpressing p38β and subjected to p38α knockdown, treated with DMSO or SB203580 (50nM, 24 h). Bottom, ELDA of tumorsphere formation showing estimated stem-cell frequency (1/x) with 95% confidence intervals and goodness-of-fit statistics. (n = 3 independent experiments). **E** Limiting dilution tumorsphere formation assays of PLC8024 parental, LacZ control and p38β-knockout cells. Bottom, ELDA of tumorsphere formation showing estimated stem-cell frequency (1/x) with 95% confidence intervals and goodness-of-fit statistics. (n = 3 independent experiments). **F-H** Densitometric quantification of the western blot bands shown in Figure 2F, p38β (**F**), p-p38 (**G**) and p-MK2 (**H**) levels were normalized to β-actin.

Data represent three independent experiments (**A**). Values are mean ± SD (**B, C, F-H**). One-way ANOVA followed by Tukey's multiple comparison (**B, C, F-H**). Two-sided ELDA likelihood-ratio test (**D, E**).

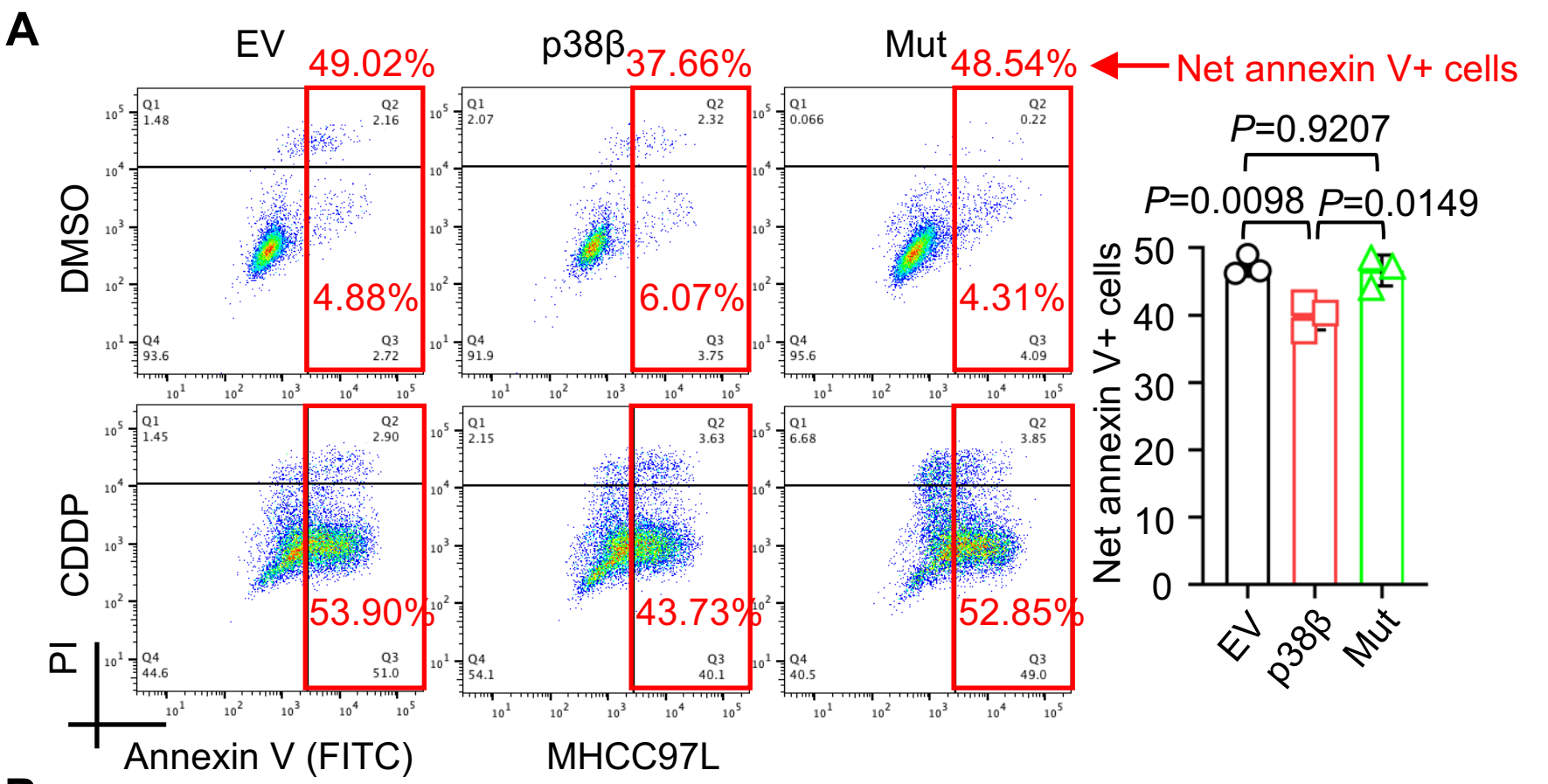

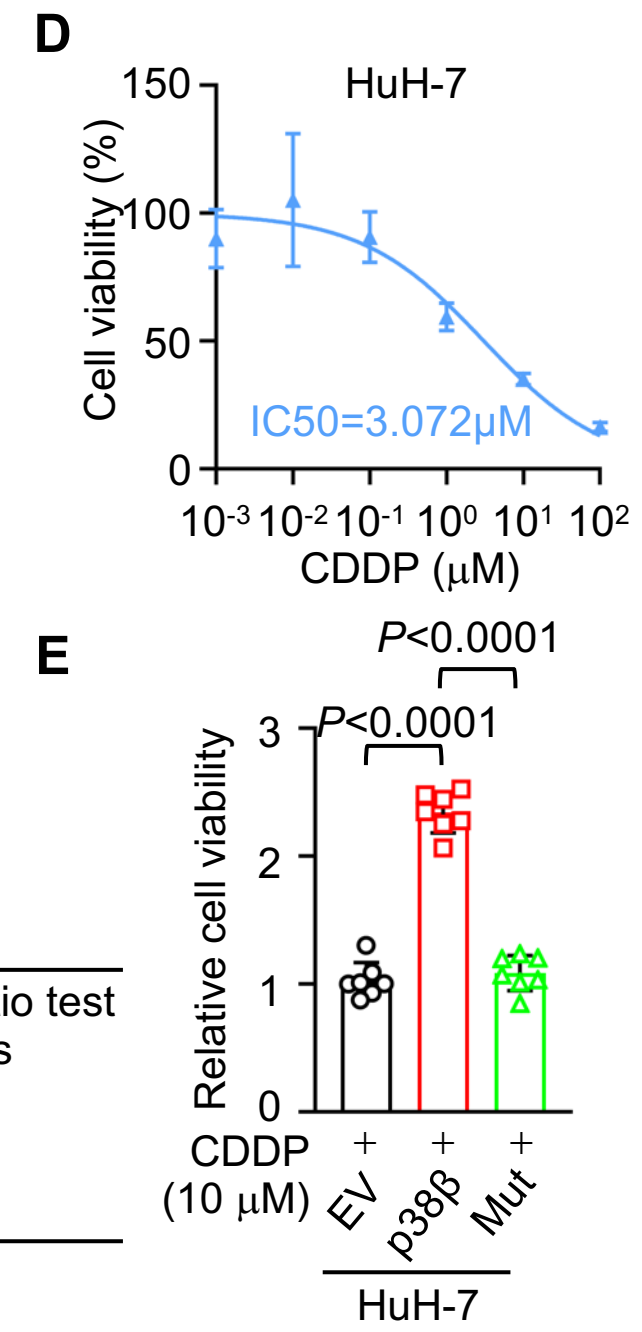

B

Cell number	Tumorsphere incidence 100	50	25	10	5	Confidence intervals Lower	Estimate	Upper	Likelihood-ratio test P-values
EV	18 / 18	16 / 18	9 / 18	3 / 18	0 / 18	1 / 45	1 / 33	1 / 24	
p38β	18 / 18	18 / 18	17 / 18	13 / 18	8 / 18	1 / 12	1 / 9	1 / 6	0.0126
Mut	18 / 18	14 / 18	12 / 18	4 / 18	1 / 18	1 / 41	1 / 30	1 / 21	

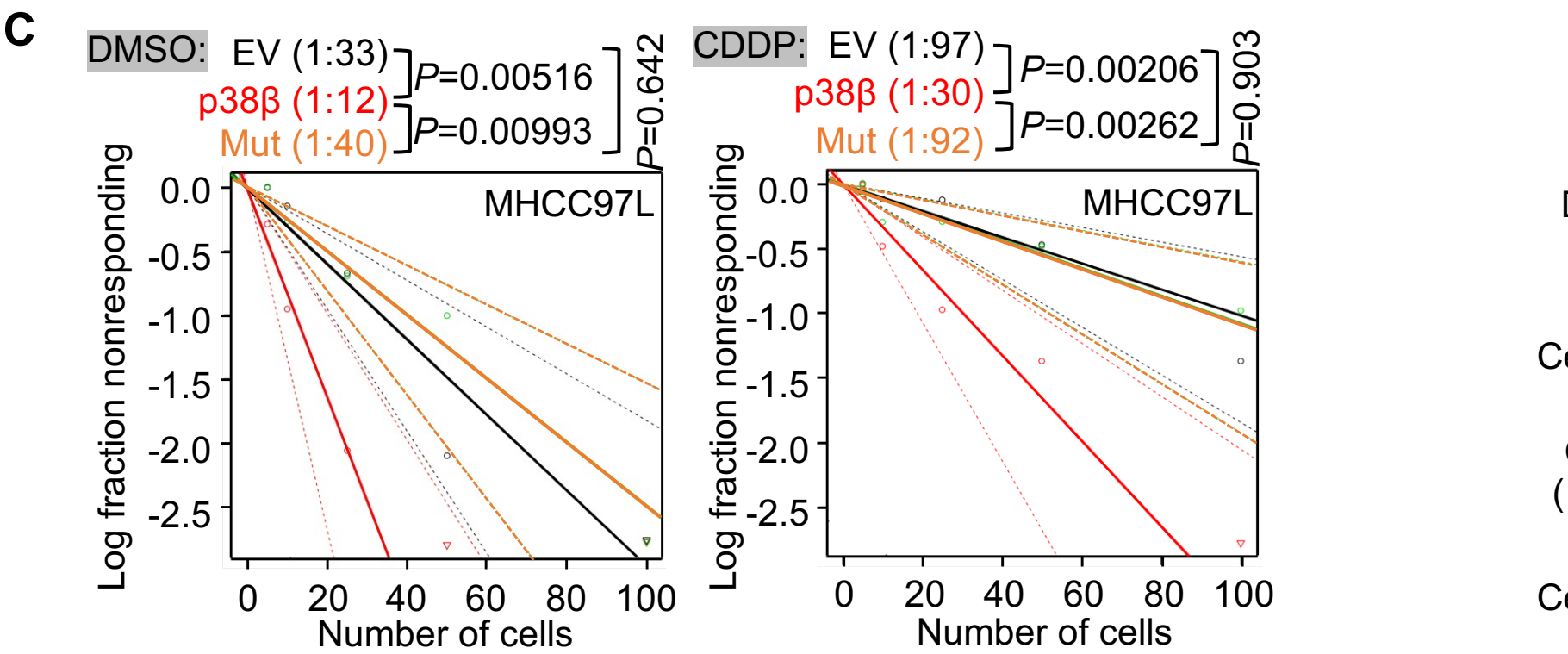

	Cell number	Tumorsphere Incidence 100	50	25	10	5	Confidence intervals Lower	Estimate	Upper	Likelihood-ratio test P-values
DMSO	EV	8 / 8	7 / 8	4 / 8	1 / 8	0 / 8	1 / 54	1 / 33	1 / 20	
	p38β	8 / 8	8 / 8	7 / 8	5 / 8	2 / 8	1 / 20	1 / 12	1 / 7	0.00243
	Mut	8 / 8	5 / 8	4 / 8	2 / 8	0 / 8	1 / 65	1 / 40	1 / 24	
CDDP	EV	6 / 8	3 / 8	1 / 8	1 / 8	0 / 8	1 / 176	1 / 97	1 / 54	
	p38β	8 / 8	6 / 8	5 / 8	3 / 8	0 / 8	1 / 44	1 / 30	1 / 18	0.00242
	Mut	5 / 8	3 / 8	2 / 8	2 / 8	0 / 8	1 / 166	1 / 92	1 / 51	

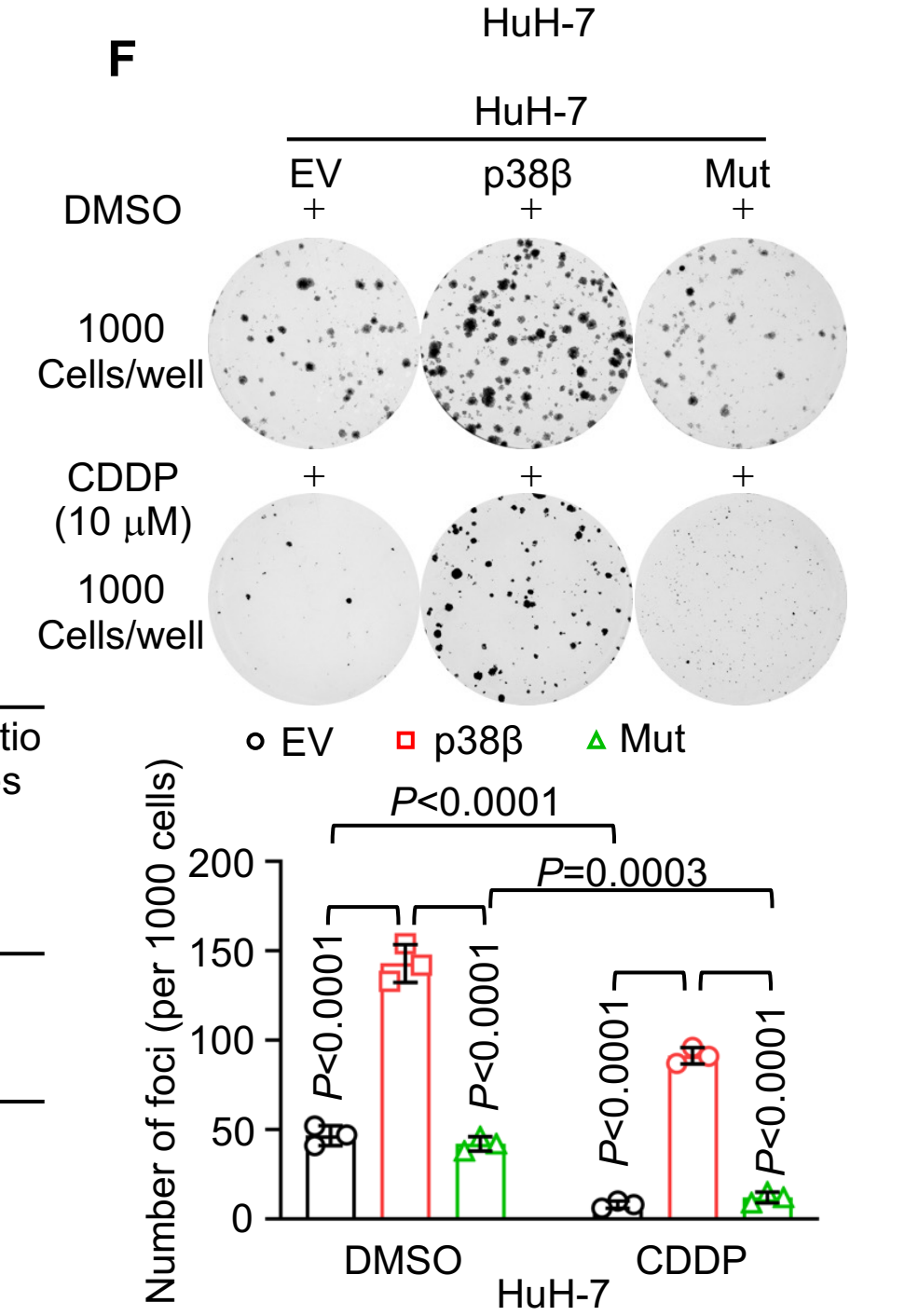

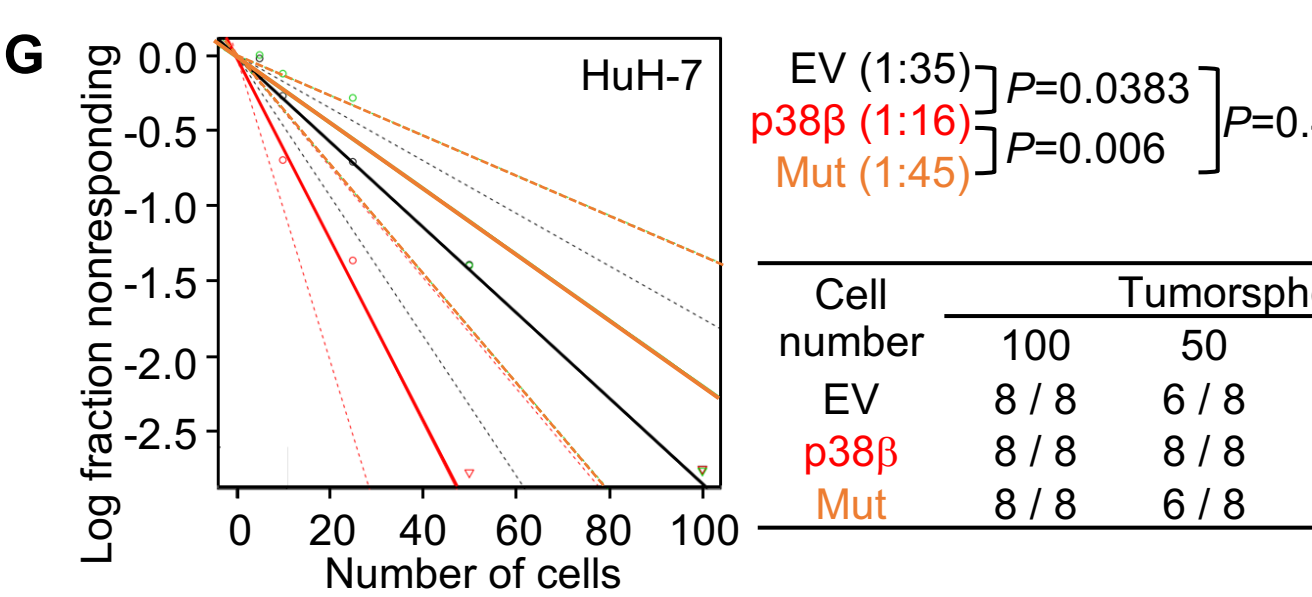

Cell number	Tumorsphere Incidence 100	50	25	10	5	Confidence intervals Lower	Estimate	Upper	Likelihood-ratio test P-values
EV	8 / 8	6 / 8	4 / 8	2 / 8	0 / 8	1 / 57	1 / 35	1 / 21	
p38β	8 / 8	8 / 8	6 / 8	4 / 8	1 / 8	1 / 27	1 / 16	1 / 9	0.0185
Mut	8 / 8	6 / 8	2 / 8	1 / 8	0 / 8	1 / 75	1 / 45	1 / 27	

Supplementary Figure 3. Wild-type p38β promotes self-renewal and tumor initiation in HCC cells. **A** Flow cytometry analysis showed a smaller proportion of Annexin V positive cells in p38β WT PLC8024 cells compared with EV and Mut after CDDP treatment (10 μM, 24 h). (n = 3 independent experiments). **B** ELDA of tumorsphere formation in MHCC97L cells overexpressing EV, p38β WT or p38β Mut showing estimated stem-cell frequency (1/x) with 95% confidence intervals and goodness-of-fit statistics. (n = 3 independent experiments). **C** ELDA comparing sphere-forming frequency in MHCC97L cells expressing EV, p38β WT or Mut treated with DMSO or CDDP (10 μM) as indicated. Top, ELDA plot with fitted lines. Bottom, estimated stem-cell frequency (1/x) with 95% confidence intervals and goodness-of-fit statistics. (n = 3 independent experiments). **D** Dose-response curves of Huh-7 cells treated with increasing concentrations of cisplatin for 72 hours, with cell viability measured by XTT assay. IC_{50} values are indicated. (n = 3 independent experiments). **E** Cell viability was measured by XTT assay in Huh-7 cells expressing EV, p38β WT or Mut following CDDP (10 μM) treatment. (n = 3 independent experiments). **F** Foci formation assays in Huh-7 cells expressing EV, p38β WT or Mut treated with DMSO or CDDP (10 μM). Bottom, quantification of foci numbers. Scale bar, 1 cm; n = 3 independent experiments. **G** ELDA comparing sphere-forming frequency in Huh-7 cells expressing EV, p38β WT or Mut. Left, ELDA plot with fitted lines. Right, estimated stem-cell frequency (1/x) with 95% confidence intervals and goodness-of-fit statistics. (n = 3 independent experiments).

Data are presented as mean ± SD (**A, E, F**). Two-sided ELDA likelihood-ratio test (**B, C, G**). One-way ANOVA followed by Tukey's multiple comparison (**A, E, F**).

A

Cell number	Tumour Latency (Days) 10000	5000	2000	1000	Tumour Incidence 10000	5000	2000	1000	Confidence intervals Lower	Estimate	Upper	Pairwise tests P-values		Likelihood-ratio test P-values
**Primary implantation**														
EV	12	27	36	64	2 / 8	2 / 8	1 / 8	1 / 8	1 / 47773	1 / 21083	1 / 9304	0.0191	0.769	0.0361
p38β	8	18	27	43	5 / 8	4 / 8	3 / 8	2 / 8	1 / 12281	1 / 6992	1 / 3980			
Mut	12	27	36	36	2 / 8	2 / 8	2 / 8	1 / 8	1 / 38492	1 / 17900	1 / 8324	0.0386		
**Secondary transplantation**														
EV	7	18	25	28	6 / 6	5 / 6	3 / 6	2 / 6	1 / 4729	1 / 2597	1 / 1426	0.0012	1.000	0.00191
p38β	4	7	18	21	6 / 6	6 / 6	6 / 6	5 / 6	1 / 1145	1 /499	1 / 218			
Mut	7	18	21	25	6 / 6	5 / 6	3 / 6	2 / 6	1 / 4729	1 / 2597	1 / 1426	0.0012		

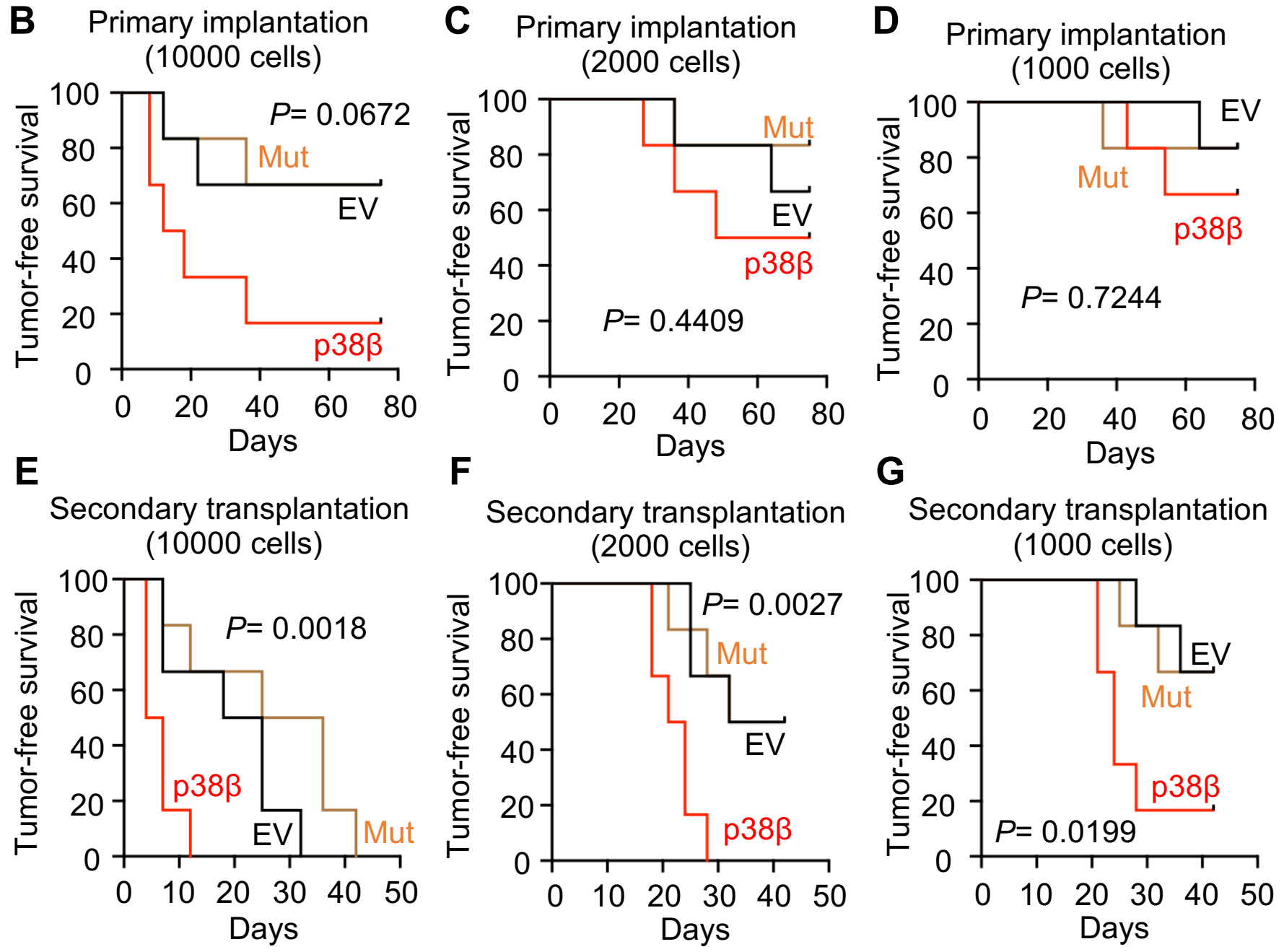

H

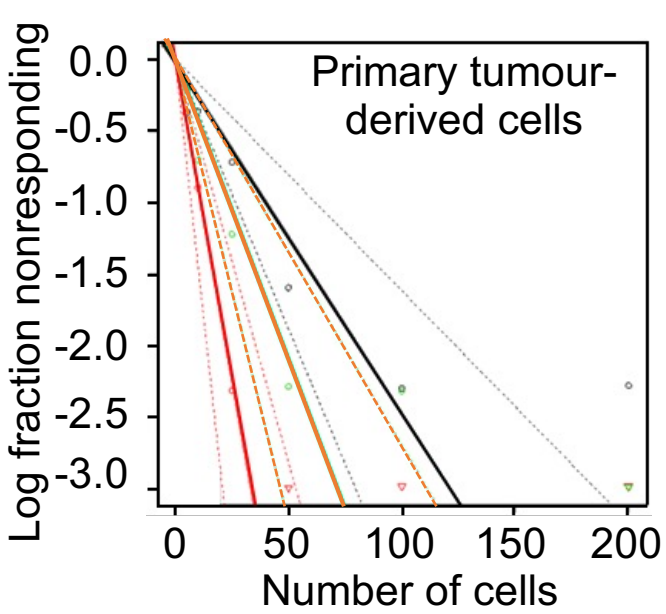

Cell number	Tumorsphere Incidence 200	100	50	25	10	5	Confidence intervals Lower	Estimate	Upper	Pairwise tests P-values		Likelihood-ratio test P-values
EV	9 / 10	9 / 10	8 / 10	5 / 10	3 / 10	3 / 10	1 / 62	1 / 41	1 / 27	0.0012	0.0724	0.000205
p38β	10 / 10	10 / 10	10 / 10	9 / 10	6 / 10	3 / 10	1 / 18	1 /11	1 / 7			
Mut	10 / 10	9 / 10	9 / 10	7 / 10	5 / 10	2 / 10	1 / 37	1 / 24	1 / 16	0.0167		

I

Cell number	Tumorsphere Incidence 1000	500	250	125	62.5	Confidence intervals Lower	Estimate	Upper	Likelihood-ratio test P-values
EV	12 / 12	10 / 12	9 / 12	6 / 12	2 / 12	1 / 326	1 / 220	1 / 149	
p38β	12 / 12	12 / 12	11 / 8	9 / 12	8 / 12	1 / 125	1 / 81	1 / 52	0.0003
Mut	12 / 12	12 / 12	7 / 8	7 / 12	2 / 12	1 / 352	1 / 238	1 / 161	

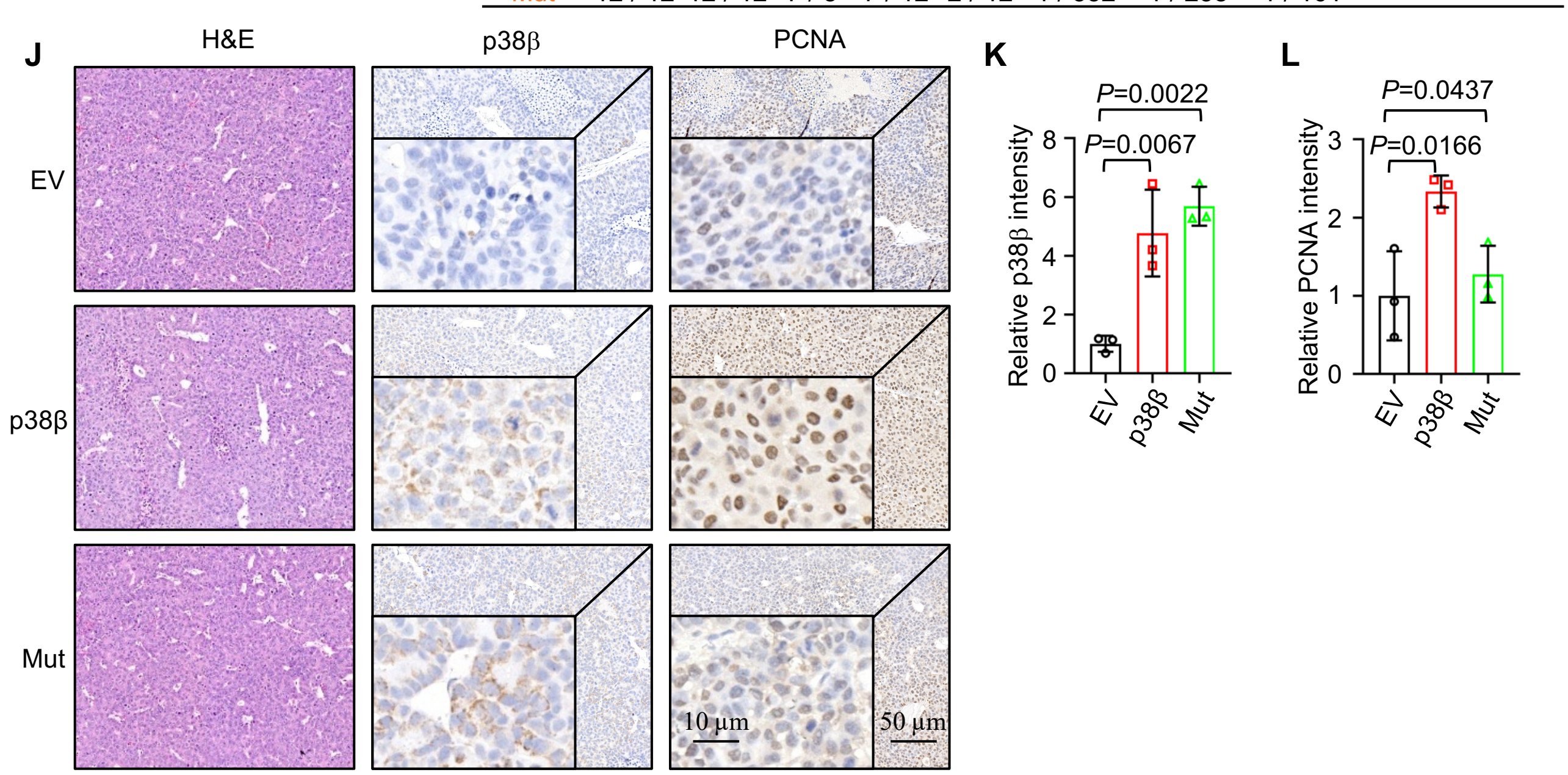

Supplementary Figure 4. AAV8-mediated p38β overexpression promotes tumorigenesis in HCC PDX. A Summary table of tumour latency/incidence for primary implantation and secondary transplantation, with ELDA of tumorsphere formation showing estimated stem-cell frequency (1/x) with 95% confidence intervals and goodness-of-fit statistics. **B-D** Tumour-free survival (Kaplan-Meier) in NOD/SCID mice following primary implantation of MHCC97L cells expressing EV, p38β WT or Mut at the indicated inoculation doses (10,000 cells (**B**), 2,000 cells (**C**) and 1,000 cells (**D**)). n = 8 mice per group. **E-G** Tumour-free survival (Kaplan-Meier) in NOD/SCID mice following secondary transplantation using tumour cells isolated from primary implantation generated xenograft tumours at the indicated doses (10,000 cells (**E**), 2,000 cells (**F**), and 1,000 cells (**G**)). n = 6 mice per group. **H** *Ex vivo* limiting dilution spheroid formation assay using dissociated tumor cells from secondary limiting dilution xenografts. Left, ELDA plot with fitted lines. Right, estimated stem-cell frequency (1/x) with 95% confidence intervals and goodness-of-fit statistics. (n = 3 independent experiments). **I** ELDA of *ex vivo* tumorsphere formation in HCC PDX overexpressing EV, p38β WT or p38β Mut showing estimated stem-cell frequency (1/x) with 95% confidence intervals and goodness-of-fit statistics. (n = 3 independent experiments). **J-L** IHC staining (**J**) and quantification of p38β (**K**) and PCNA (**L**) in established HCC PDX tumours following intratumoral AAV8-mediated overexpression of EV, p38β WT or Mut. Representative IHC images are shown in (**J**). Scale bar, 50 μm in the main images and 10 μm in the enlarged insets. Data represent three random fields and are presented as mean ± SD (**J-L**), one-way ANOVA followed by Tukey's multiple comparison (**K**, **L**).

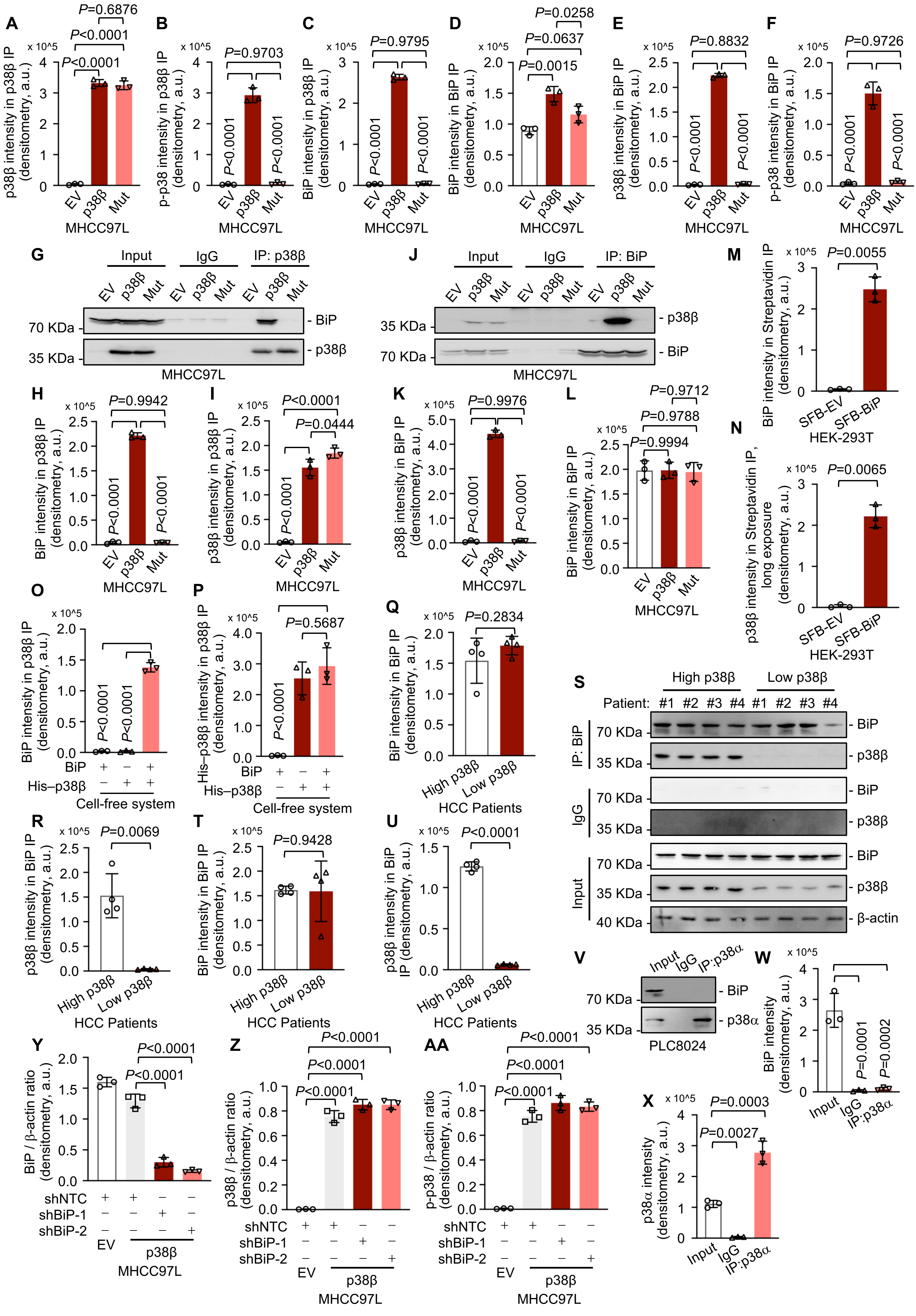
A
x 10^5
P=0.6876
P<0.0001
P<0.0001
p38β intensity in p38β IP (densitometry, a.u.)
EV p38β Mut
MHCC97L
B
x 10^5
P=0.9703
P<0.0001
P<0.0001
p-p38 intensity in p38β IP (densitometry, a.u.)
EV p38β Mut
MHCC97L
C
x 10^5
P=0.9795
P<0.0001
P<0.0001
BiP intensity in p38β IP (densitometry, a.u.)
EV p38β Mut
MHCC97L
D
x 10^5
P=0.0258
P=0.0637
P=0.0015
BiP intensity in BiP IP (densitometry, a.u.)
EV p38β Mut
MHCC97L
E
x 10^5
P=0.8832
P<0.0001
P<0.0001
p38β intensity in BiP IP (densitometry, a.u.)
EV p38β Mut
MHCC97L
F
x 10^5
P=0.9726
P<0.0001
P<0.0001
p-p38 intensity in BiP IP (densitometry, a.u.)
EV p38β Mut
MHCC97L
G
Input IgG IP: p38β
EV p38β Mut EV p38β Mut EV p38β Mut
70 KDa - BiP
35 KDa - p38β
MHCC97L
J
Input IgG IP: BiP
EV p38β Mut EV p38β Mut EV p38β Mut
35 KDa - p38β
70 KDa - BiP
MHCC97L
M
x 10^5
P=0.0055
BiP intensity in Streptavidin IP (densitometry, a.u.)
SFB-EV SFB-BiP
HEK-293T
H
x 10^5
P=0.9942
P<0.0001
P<0.0001
BiP intensity in p38β IP (densitometry, a.u.)
EV p38β Mut
MHCC97L
I
x 10^5
P<0.0001
P=0.0444
P<0.0001
p38β intensity in p38β IP (densitometry, a.u.)
EV p38β Mut
MHCC97L
K
x 10^5
P=0.9976
P<0.0001
P<0.0001
p38β intensity in BiP IP (densitometry, a.u.)
EV p38β Mut
MHCC97L
L
x 10^5
P=0.9712
P=0.9788
P=0.9994
BiP intensity in BiP IP (densitometry, a.u.)
EV p38β Mut
MHCC97L
N
x 10^5
P=0.0065
p38β intensity in Streptavidin IP, long exposure (densitometry, a.u.)
SFB-EV SFB-BiP
HEK-293T
O
x 10^5
P<0.0001
P<0.0001
BiP intensity in p38β IP (densitometry, a.u.)
BiP + − +
His–p38β − + +
Cell-free system
P
x 10^5
P=0.5687
P<0.0001
His–p38β intensity in p38β IP (densitometry, a.u.)
BiP + − +
His–p38β − + +
Cell-free system
Q
x 10^5
P=0.2834
BiP intensity in BiP IP (densitometry, a.u.)
High p38β Low p38β
HCC Patients
S
High p38β Low p38β
Patient: #1 #2 #3 #4 #1 #2 #3 #4
IP: BiP
70 KDa - BiP
35 KDa - p38β
IgG
70 KDa - BiP
35 KDa - p38β
Input
70 KDa - BiP
35 KDa - p38β
40 KDa - β-actin
R
x 10^5
P=0.0069
p38β intensity in BiP IP (densitometry, a.u.)
High p38β Low p38β
HCC Patients
T
x 10^5
P=0.9428
BiP intensity in BiP IP (densitometry, a.u.)
High p38β Low p38β
HCC Patients
U
x 10^5
P<0.0001
p38β intensity in BiP IP (densitometry, a.u.)
High p38β Low p38β
HCC Patients
V
Input IgG IP:p38α
70 KDa - BiP
35 KDa - p38α
PLC8024
W
x 10^5
P=0.0001
P=0.0002
BiP intensity (densitometry, a.u.)
Input IgG IP:p38α
X
x 10^5
P=0.0003
P=0.0027
p38α intensity (densitometry, a.u.)
Input IgG IP:p38α
Y
P<0.0001
P<0.0001
BiP / β-actin ratio (densitometry, a.u.)
shNTC + + − −
shBiP-1 − − + −
shBiP-2 − − − +
EV p38β
MHCC97L
Z
P<0.0001
P<0.0001
P<0.0001
p38β / β-actin ratio (densitometry, a.u.)
shNTC + + − −
shBiP-1 − − + −
shBiP-2 − − − +
EV p38β
MHCC97L
AA
P<0.0001
P<0.0001
P<0.0001
p-p38 / β-actin ratio (densitometry, a.u.)
shNTC + + − −
shBiP-1 − − + −
shBiP-2 − − − +
EV p38β
MHCC97L

Supplementary Figure 5. p38β specifically interacts with BiP in HCC. A-F Densitometric quantification of the co-IP assays shown in Figure 3B (**A-C**) and Figure 3C (**D-F**). **G-L** Co-IP assays using antibodies against p38β (**G**) or BiP (**J**) in MHCC97L cells expressing EV, p38β WT or Mut, with corresponding densitometric quantification (**H, I, K, L**). **M-R** Densitometric quantification of the pull-down and immunoprecipitation assays shown in Figure 3D (**M, N**), Figure 3E (**O, P**), and Figure 3F (**Q, R**). **S-U** Endogenous p38β-BiP interaction in patient-derived HCC tissues with high or low p38β expression was assessed by co-IP (**S**), with corresponding densitometric quantification (**T, U**). **V-X** Endogenous p38α immunoprecipitation in PLC8024 cells showed no detectable co-precipitation of BiP (**V**), with corresponding densitometric quantification (**W, X**). For panels G, J, S and V, samples within each experiment derived from the same experiment, but separate gels for the indicated proteins and controls were processed in parallel. **Y-AA** Densitometric quantification of the western blot bands shown in Figure 3H, BiP (**Y**), p38β (**Z**), and p-p38 (**AA**) levels were normalized to β-actin.

Data represent three independent experiments (**G, J, S, V**). Values are mean ± SD (**A-F, H, I, K-R, T, U, W-AA**). One-way ANOVA followed by Tukey's multiple comparison (**A-F, H, I, K, L, O, P, W-AA**). Two-tailed unpaired Student's t-test (**M, N, Q, R, T, U**).

A

Cell number	Tumorsphere Incidence					Confidence intervals			Overall test *P*-values
	100	50	25	10	5	Lower	Estimate	Upper	
EV+shNTC	11 / 12	9 / 12	6 / 12	4 / 12	1 / 12	1 / 55	1 / 37	1 / 25	
p38β+shNTC	12 / 12	12 / 12	12 / 12	7 / 12	3 / 12	1 / 16	1 /11	1 / 7	<0.001
p38β+shBiP-1	10 / 12	5 / 12	2 / 12	1 / 12	0 / 12	1 / 135	1 / 85	1 / 53	
p38β+shBiP-2	11 / 12	6 / 12	4 / 12	2 / 12	1 / 12	1 / 85	1 / 56	1 / 37	

B **C**

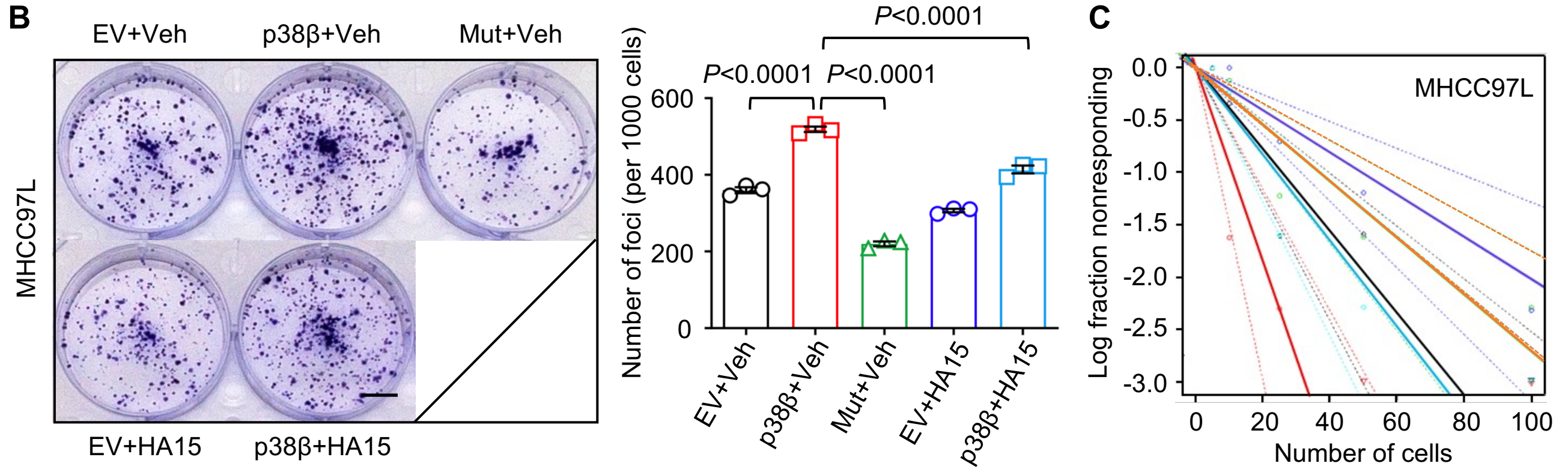

Cell number	Tumorsphere Incidence					Confidence intervals			Pairwise tests P-values		Likelihood-ratio test P-values
	100	50	25	10	5	Lower	Estimate	Upper			
EV+Veh	10 / 10	8 / 10	8 / 10	3 / 10	0 / 10	1 / 40	1 / 26	1 / 17	0.00952		
p38β+Veh	10 / 10	10 / 10	9 / 10	8 / 10	1 / 10	1 / 17	1 /11	1 / 7			
Mut+Veh	9 / 10	8 / 10	7 / 10	1 / 10	0 / 10	1 / 57	1 / 37	1 / 24	0.00202	0.0169	0.000137
EV+HA15	9 / 10	7 / 10	5 / 10	0 / 10	0 / 10	1 / 78	1 / 49	1 / 31			
p38β+HA15	10 / 10	9 / 10	8 / 10	2 / 10	0 / 10	1 / 37	1 / 24	1 / 16			

D

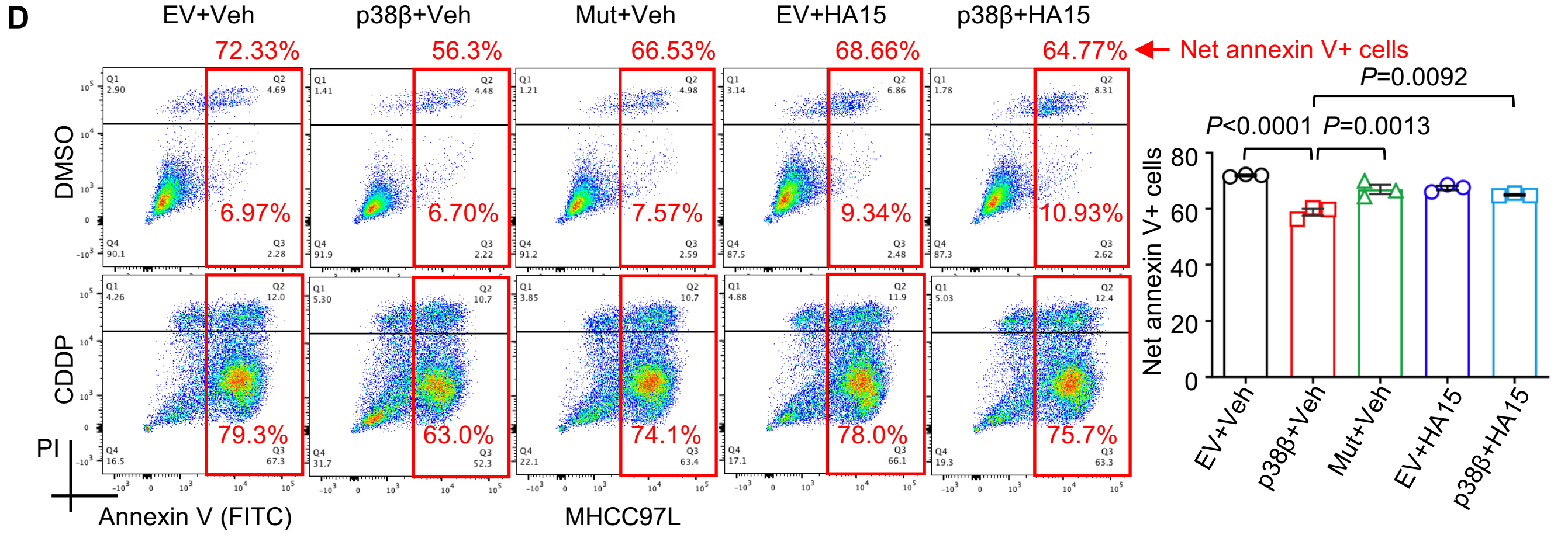

Supplementary Figure 6. BiP mediates p38β-induced stemness and drug resistance in HCC. A ELDA of tumorsphere formation showing estimated stem-cell frequency (1/x) with 95% confidence intervals and goodness-of-fit statistics in MHCC97L cells overexpressing p38β with BiP knockdown. **B** Foci formation assays in MHCC97L cells expressing EV, p38β WT, or Mut, with or without HA15 (5 µM, 24 h) treatment. Right, quantification of foci numbers. Scale bar, 1 cm; n = 3 independent experiments. **C** ELDA comparing sphere-forming frequency in MHCC97L cells expressing EV, p38β WT, or Mut, with or without HA15 (5 µM, 24 h) treatment. Top, ELDA plot with fitted lines. Bottom, estimated stem-cell frequency (1/x) with 95% confidence intervals and goodness-of-fit statistics. (n = 3 independent experiments). **D** Flow cytometry analysis showed that BiP inhibition by HA15 (5 µM, 24 h) restored the decreased proportion of Annexin V positive cells caused by p38β overexpression in MHCC97L cells after CDDP (10 µM, 24 h) treatment, whereas HA15 had no effect in cells expressing EV or p38β Mut. (n = 3 independent experiments).

Data are presented as mean ± SD (**B, D**). Two-sided ELDA likelihood-ratio test (**A, C**). One-way ANOVA followed by Tukey's multiple comparison (**B, D**).

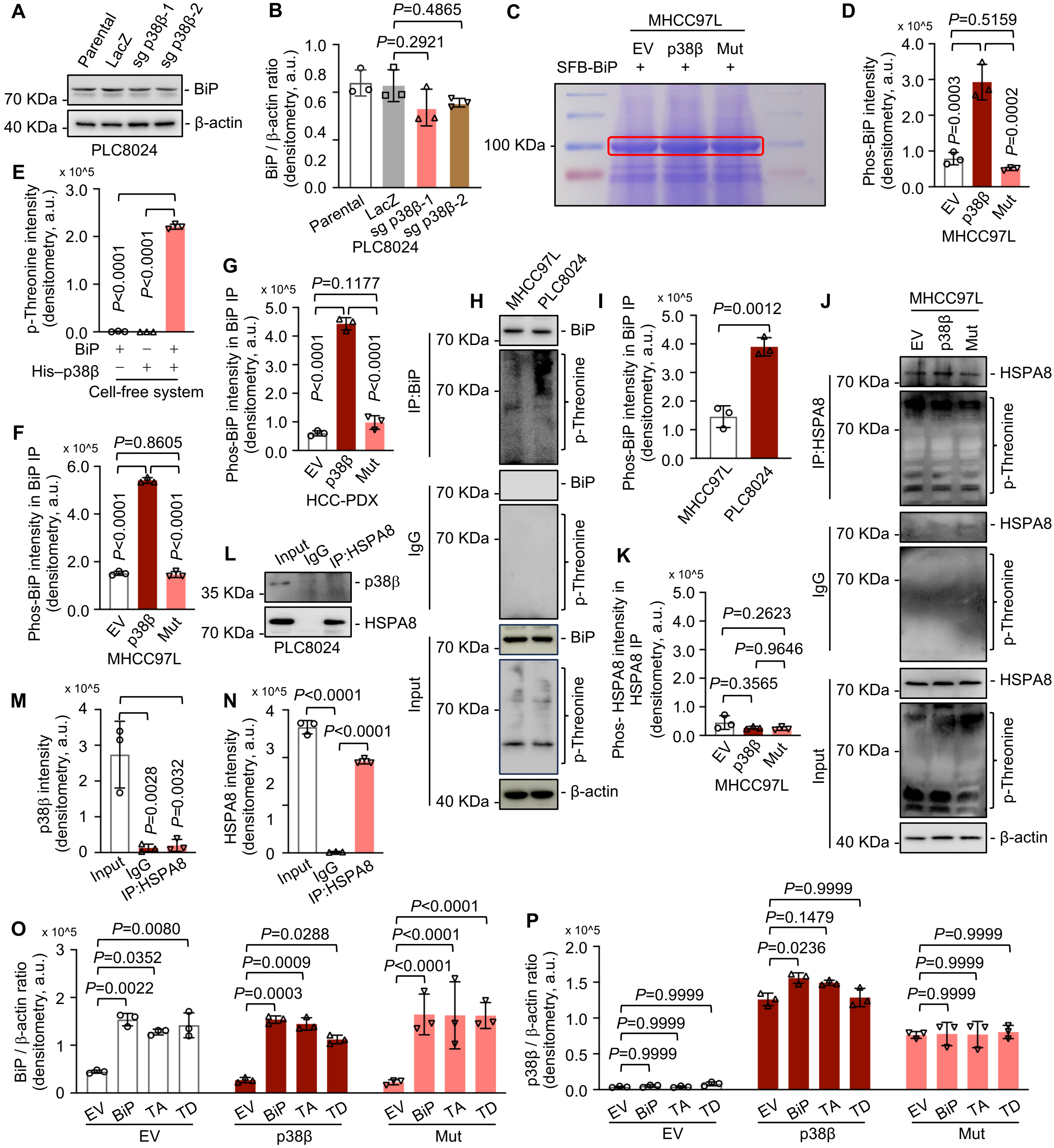
A
Parental
LacZ
sg p38β-1
sg p38β-2
70 KDa
BiP
40 KDa
β-actin
PLC8024
B
P=0.4865
P=0.2921
BiP / β-actin ratio (densitometry, a.u.)
1.0
0.8
0.6
0.4
0.2
0.0
Parental
LacZ
sg p38β-1
sg p38β-2
PLC8024
C
MHCC97L
EV
p38β
Mut
SFB-BiP
+
100 KDa
D
x 10^5
P=0.5159
Phos-BiP intensity (densitometry, a.u.)
4.0
3.0
2.0
1.0
0.0
P=0.0003
P=0.0002
EV
p38β
Mut
MHCC97L
E
x 10^5
p-Threonine intensity (densitometry, a.u.)
3.0
2.0
1.0
0.0
P<0.0001
P<0.0001
BiP
His–p38β
Cell-free system
F
x 10^5
P=0.8605
Phos-BiP intensity in BiP IP (densitometry, a.u.)
6.0
4.0
2.0
0.0
P<0.0001
P<0.0001
EV
p38β
Mut
MHCC97L
G
x 10^5
P=0.1177
Phos-BiP intensity in BiP IP (densitometry, a.u.)
5.0
4.0
3.0
2.0
1.0
0.0
P<0.0001
P<0.0001
EV
p38β
Mut
HCC-PDX
H
MHCC97L
PLC8024
IP:BiP
IgG
Input
70 KDa
40 KDa
BiP
p-Threonine
β-actin
I
x 10^5
P=0.0012
Phos-BiP intensity in BiP IP (densitometry, a.u.)
5.0
4.0
3.0
2.0
1.0
0.0
MHCC97L
PLC8024
J
MHCC97L
EV
p38β
Mut
IP:HSPA8
IgG
Input
70 KDa
40 KDa
HSPA8
p-Threonine
β-actin
K
x 10^5
Phos- HSPA8 intensity in HSPA8 IP (densitometry, a.u.)
5.0
4.0
3.0
2.0
1.0
0.0
P=0.2623
P=0.9646
P=0.3565
EV
p38β
Mut
MHCC97L
L
Input
IgG
IP:HSPA8
35 KDa
70 KDa
p38β
HSPA8
PLC8024
M
x 10^5
p38β intensity (densitometry, a.u.)
4
3
2
1
0
P=0.0028
P=0.0032
Input
IgG
IP:HSPA8
N
x 10^5
P<0.0001
P<0.0001
HSPA8 intensity (densitometry, a.u.)
4
3
2
1
0
Input
IgG
IP:HSPA8
O
x 10^5
BiP / β-actin ratio (densitometry, a.u.)
3
2
1
0
P=0.0080
P=0.0352
P=0.0022
P=0.0288
P=0.0009
P=0.0003
P<0.0001
P<0.0001
P<0.0001
EV
BiP
TA
TD
EV
p38β
Mut
P
x 10^5
p38β / β-actin ratio (densitometry, a.u.)
2.0
1.5
1.0
0.5
0.0
P=0.9999
P=0.9999
P=0.9999
P=0.9999
P=0.1479
P=0.0236
P=0.9999
P=0.9999
P=0.9999
EV
BiP
TA
TD
EV
p38β
Mut

Supplementary Figure 7. BiP phosphorylation is driven by p38β overexpression. A, B Western blot analysis of BiP expression in p38β-knockout PLC8024 cells (**A**), with corresponding densitometric quantification normalized to β-actin (**B**). **C** Coomassie Blue Staining of immunoprecipitated SFB-tagged BiP in MHCC97L cells overexpressing EV, p38β WT and Mut. Gel bands highlighted in red box were sliced and sent for phosphoproteomics profiling to identify BiP phosphorylation residues. **D-G** Densitometric quantification of phosphorylated BiP detected by Phos-tag SDS-PAGE (**D**), *in vitro* kinase assay (**E**), and BiP immunoprecipitation in MHCC97L cells expressing EV, p38β WT or Mut (**F**) and HCC PDX tumours (**G**), corresponding to Figure 4C-F, respectively. **H, I** Immunoblot analysis of BiP phosphorylation in MHCC97L (p38β-low) and PLC8024 (p38β-high) HCC cells, assessed by BiP immunoprecipitation followed by immunoblotting with pan-phospho-threonine and the indicated antibodies (**H**), with corresponding densitometric quantification (**I**). **J, K** HSPA8 phosphorylation in MHCC97L cells expressing EV, p38β WT or Mut was assessed by HSPA8 immunoprecipitation and pan-phospho-threonine immunoblotting (**J**), with corresponding densitometric quantification (**K**). **L-N** Co-immunoprecipitation analysis showed no detectable interaction between endogenous HSPA8 and p38β in PLC8024 cells (**L**), with corresponding densitometric quantification (**M, N**). For panels A, H, J and L, samples within each experiment derived from the same experiment, but separate gels for the indicated proteins and controls were processed in parallel. **O, P** Densitometric quantification of BiP (**O**) and p38β (**P**) protein levels shown in Figure 4K.

Data represent three independent experiments (**A, H, J, L**). Values are mean ± SD (**B, D-G, I, K, M-P**). One-way ANOVA followed by Tukey's multiple comparison (**B, D-G, K, M-P**). Two-tailed unpaired Student's t-test (**I**).

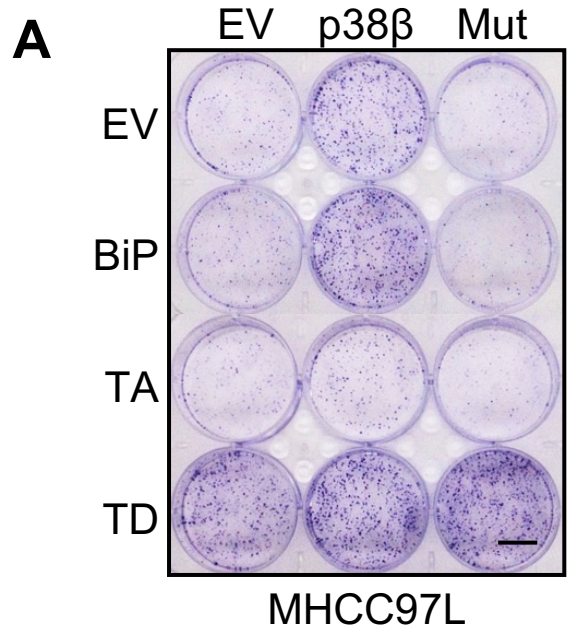

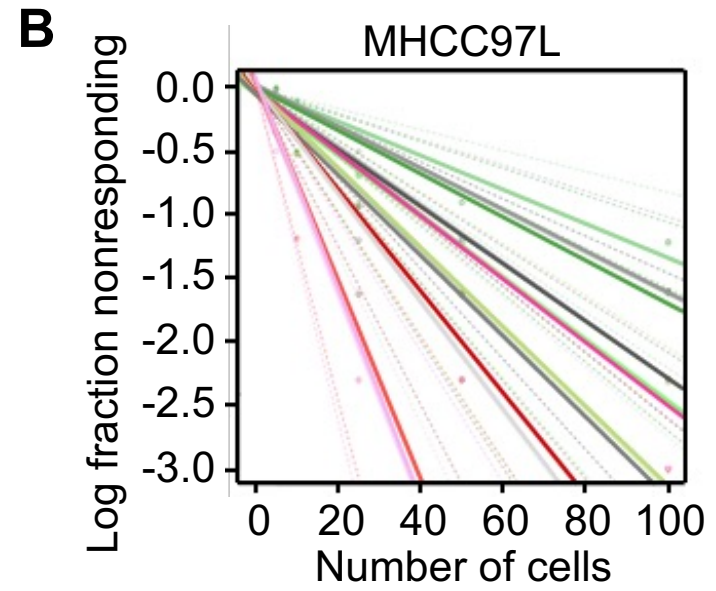

Cell number		Tumorsphere Incidence 100	50	25	10	5	Confidence intervals Lower	Estimate	Upper	Likelihood-ratio test P-values
EV	EV	8 / 10	7 / 10	6 / 10	3 / 10	0 / 10	1 / 69	1 / 44	1 / 28	
	BiP	9 / 10	8 / 10	7 / 10	4 / 10	0 / 10	1 / 48	1 /31	1 / 20	
	TA	8 / 10	6 / 10	4 / 10	1 / 10	0 / 10	1 / 98	1 / 61	1 / 38	
	TD	9 / 10	9 / 10	8 / 10	5 / 10	1 / 10	1 / 37	1 / 24	1 / 15	
p38β	EV	9 / 10	9 / 10	8 / 10	4 / 10	1 / 10	1 / 39	1 / 25	1 / 16	
	BiP	10 / 10	9 / 10	9 / 10	7 / 10	3 / 10	1 / 21	1 / 13	1 / 8	
	TA	8 / 10	8 / 10	6 / 10	3 / 10	0 / 10	1 / 63	1 / 41	1 / 26	<0.001
	TD	10 / 10	9 / 10	9 / 10	7 / 10	4 / 10	1 / 19	1 / 12	1 / 8	
Mut	EV	7 / 10	7 / 10	5 / 10	1 / 10	0 / 10	1 / 95	1 / 59	1 / 37	
	BiP	8 / 10	8 / 10	5 / 10	4 / 10	0 / 10	1 / 64	1 / 41	1 / 26	
	TA	7 / 10	6 / 10	3 / 10	1 / 10	0 / 10	1 / 122	1 / 74	1 / 45	
	TD	9 / 10	8 / 10	6 / 10	4 / 10	1 / 10	1 / 49	1 / 32	1 / 20	

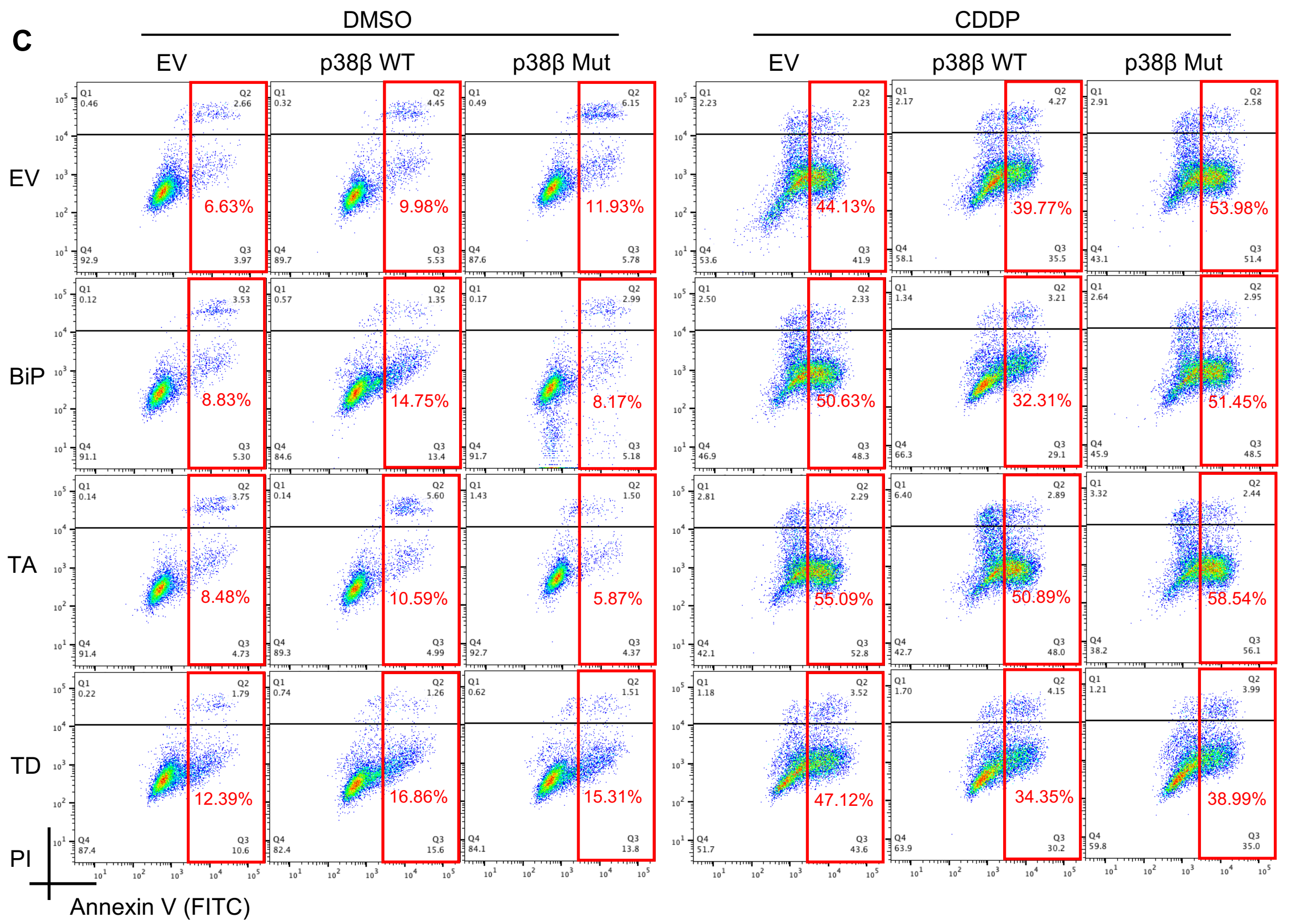

D

Primary Impantation		Tumour Latency (Days) 10000	5000	2000	Tumour Counting 10000	5000	2000	Confidence intervals Lower	Estimate	Upper	Pairwise tests P-values		Likelihood-ratio test P-values
p38β	EV	14	32	/	3 / 6	2 / 6	0 / 6	1 / 38561	1 / 16038	1 / 6671	0.0143	0.187	
	BiP	10	24	36	5 / 6	4 / 6	3 / 6	1 / 8420	1 / 4438	1 / 2339			<0.001
	TA	38	/	/	2 / 6	0 / 6	0 / 6	1 / 180249	1 / 45818	1 / 11647			
	TD	10	15	36	6 / 6	6 / 6	6 / 6	1 / 1876	1 / 1	1 / 1	<0.0001		

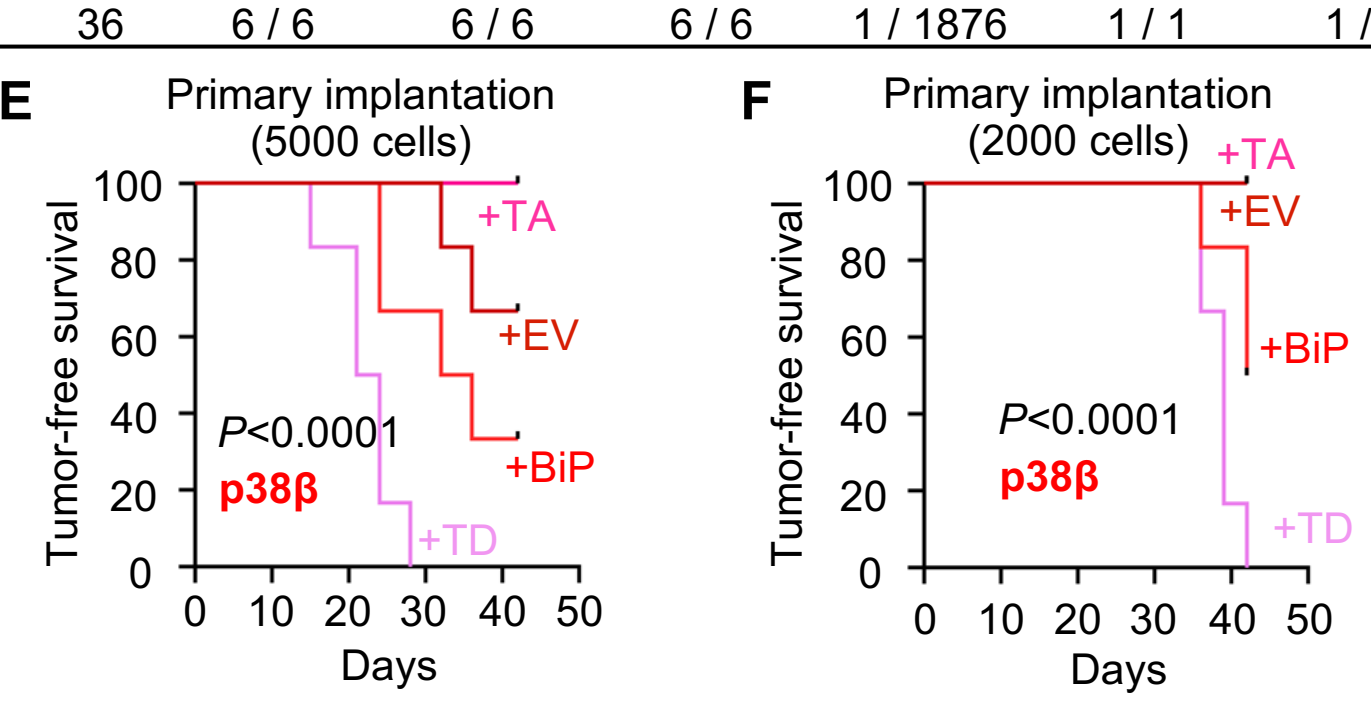

Supplementary Figure 8. BiP Thr648 phosphorylation is essential for p38β-driven stemness and drug resistance in HCC. A Representative images of foci formation assays in MHCC97L cells expressing EV, p38β WT, or Mut, each co-expressing either EV, BiP, TA, or TD. Data represent three independent experiments. **B** ELDA of tumorsphere formation in MHCC97L cells expressing EV, p38β WT, or Mut, each co-expressing either EV, BiP, TA, or TD. Bottom, estimated stem-cell frequency (1/x) with 95% confidence intervals and goodness-of-fit statistics. (n = 3 independent experiments). **C** Flow cytometry analysis of Annexin V-positive cell populations in MHCC97L cells expressing EV, p38β WT, or Mut, each co-expressing either EV, BiP, TA, or TD. Data represent three independent experiments. **D** Summary table of tumour latency and incidence for primary implantation of p38β-overexpressing MHCC97L cells co-expressing EV, BiP, TA, or TD, with ELDA of tumorsphere formation showing estimated stem-cell frequency (1/x) with 95% confidence intervals and goodness-of-fit statistics. (n = 6 mice per group). **E, F** Limiting dilution transplantation assays were performed in NOD/SCID mice using p38β-overexpressing MHCC97L cells co-expressing EV, BiP WT, TA or TD. Tumour-free survival of mice injected with 5,000 (**E**) or 2,000 (**F**) cells was assessed by Kaplan-Meier analysis. (n = 6 mice per group).

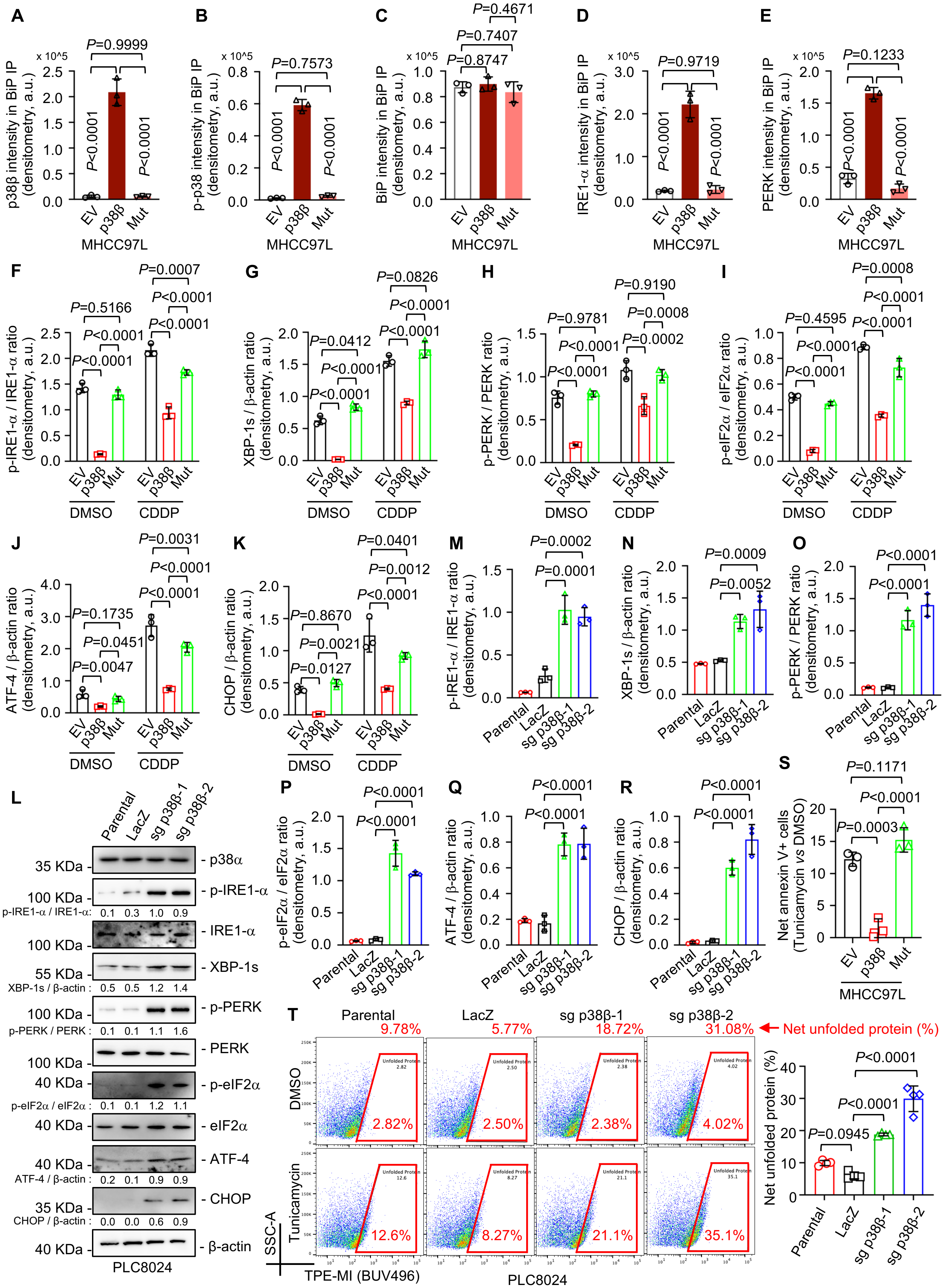
A
x 10^5
P=0.9999
P<0.0001
P<0.0001
p38β intensity in BiP IP (densitometry, a.u.)
EV p38β Mut
MHCC97L
B
x 10^5
P=0.7573
P<0.0001
P<0.0001
p-p38 intensity in BiP IP (densitometry, a.u.)
EV p38β Mut
MHCC97L
C
P=0.4671
P=0.7407
P=0.8747
x 10^5
BiP intensity in BiP IP (densitometry, a.u.)
EV p38β Mut
MHCC97L
D
x 10^5
P=0.9719
P<0.0001
P<0.0001
IRE1-α intensity in BiP IP (densitometry, a.u.)
EV p38β Mut
MHCC97L
E
x 10^5
P=0.1233
P<0.0001
P<0.0001
PERK intensity in BiP IP (densitometry, a.u.)
EV p38β Mut
MHCC97L
F
P=0.0007
P<0.0001
P=0.5166
P<0.0001
P<0.0001
P<0.0001
p-IRE1-α / IRE1-α ratio (densitometry, a.u.)
EV p38β Mut EV p38β Mut
DMSO CDDP
G
P=0.0826
P<0.0001
P=0.0412
P<0.0001
P<0.0001
P<0.0001
XBP-1s / β-actin ratio (densitometry, a.u.)
EV p38β Mut EV p38β Mut
DMSO CDDP
H
P=0.9190
P=0.9781
P=0.0008
P<0.0001
P=0.0002
P<0.0001
p-PERK / PERK ratio (densitometry, a.u.)
EV p38β Mut EV p38β Mut
DMSO CDDP
I
P=0.0008
P<0.0001
P=0.4595
P<0.0001
P<0.0001
P<0.0001
p-eIF2α / eIF2α ratio (densitometry, a.u.)
EV p38β Mut EV p38β Mut
DMSO CDDP
J
P=0.0031
P<0.0001
P<0.0001
P=0.1735
P=0.0451
P=0.0047
ATF-4 / β-actin ratio (densitometry, a.u.)
EV p38β Mut EV p38β Mut
DMSO CDDP
K
P=0.0401
P=0.0012
P<0.0001
P=0.8670
P=0.0021
P=0.0127
CHOP / β-actin ratio (densitometry, a.u.)
EV p38β Mut EV p38β Mut
DMSO CDDP
M
P=0.0002
P=0.0001
p-IRE1-α / IRE1-α ratio (densitometry, a.u.)
Parental LacZ sg p38β-1 sg p38β-2
N
P=0.0009
P=0.0052
XBP-1s / β-actin ratio (densitometry, a.u.)
Parental LacZ sg p38β-1 sg p38β-2
O
P<0.0001
P<0.0001
p-PERK / PERK ratio (densitometry, a.u.)
Parental LacZ sg p38β-1 sg p38β-2
L
Parental LacZ sg p38β-1 sg p38β-2
35 KDa - p38α
100 KDa - p-IRE1-α
p-IRE1-α / IRE1-α: 0.1 0.3 1.0 0.9
100 KDa - IRE1-α
55 KDa - XBP-1s
XBP-1s / β-actin : 0.5 0.5 1.2 1.4
100 KDa - p-PERK
p-PERK / PERK : 0.1 0.1 1.1 1.6
100 KDa - PERK
40 KDa - p-eIF2α
p-eIF2α / eIF2α : 0.1 0.1 1.2 1.1
40 KDa - eIF2α
40 KDa - ATF-4
ATF-4 / β-actin : 0.2 0.1 0.9 0.9
35 KDa - CHOP
CHOP / β-actin : 0.0 0.0 0.6 0.9
40 KDa - β-actin
PLC8024
P
P<0.0001
P<0.0001
p-eIF2α / eIF2α ratio (densitometry, a.u.)
Parental LacZ sg p38β-1 sg p38β-2
Q
P<0.0001
P<0.0001
ATF-4 / β-actin ratio (densitometry, a.u.)
Parental LacZ sg p38β-1 sg p38β-2
R
P<0.0001
P<0.0001
CHOP / β-actin ratio (densitometry, a.u.)
Parental LacZ sg p38β-1 sg p38β-2
S
P=0.1171
P<0.0001
P=0.0003
Net annexin V+ cells (Tunicamycin vs DMSO)
EV p38β Mut
MHCC97L
T
Parental LacZ sg p38β-1 sg p38β-2
9.78% 5.77% 18.72% 31.08%
Net unfolded protein (%)
DMSO
2.82% 2.50% 2.38% 4.02%
Tunicamycin
12.6% 8.27% 21.1% 35.1%
SSC-A
TPE-MI (BUV496)
PLC8024
P<0.0001
P<0.0001
P=0.0945
Net unfolded protein (%)
Parental LacZ sg p38β-1 sg p38β-2

Supplementary Figure 9. p38β suppresses UPR pathway activation and unfolded protein accumulation in HCC cells. A-E Densitometric quantification of the co-IP assays shown in Figure 5A. **F-K** Densitometric quantification of western blot bands shown in Figure 5B, p-IRE1-α was normalized to total IRE1-α (**F**), XBP-1s to β-actin (**G**), p-PERK to total PERK (**H**), p-eIF2α to total eIF2α (**I**), ATF-4 to β-actin (**J**), and CHOP to β-actin (**K**). Values are expressed relative to the control group. **L-R** Western blot analysis of UPR pathway activation in p38β-knockout PLC8024 cells (**L**) and corresponding densitometric quantification. The samples derive from the same experiment, but separate gels for indicated proteins were processed in parallel. p-IRE1-α was normalized to total IRE1-α (**M**), XBP-1s to β-actin (**N**), p-PERK to total PERK (**O**), p-eIF2α to total eIF2α (**P**), ATF-4 to β-actin (**Q**), and CHOP to β-actin (**R**). Values are expressed relative to the control group. **S** Flow cytometry analysis of Annexin V-positive cell populations in MHCC97L cells expressing EV, p38β WT, or Mut, following DMSO or Tunicamycin (0.5μg/mL, 24 h) treatment. **T** Flow cytometry analysis using TPE-MI to assess unfolded protein accumulation in PLC8024 cells with p38β knockout, following DMSO or Tunicamycin (0.5μg/mL, 24 h) treatment. Right, quantification of net unfolded protein (%). (n = 4 independent experiments).

Data represent three independent experiments (**L**). Values are mean ± SD (**A-K, M-T**). One-way ANOVA followed by Tukey's multiple comparison (**A-K, M-T**).

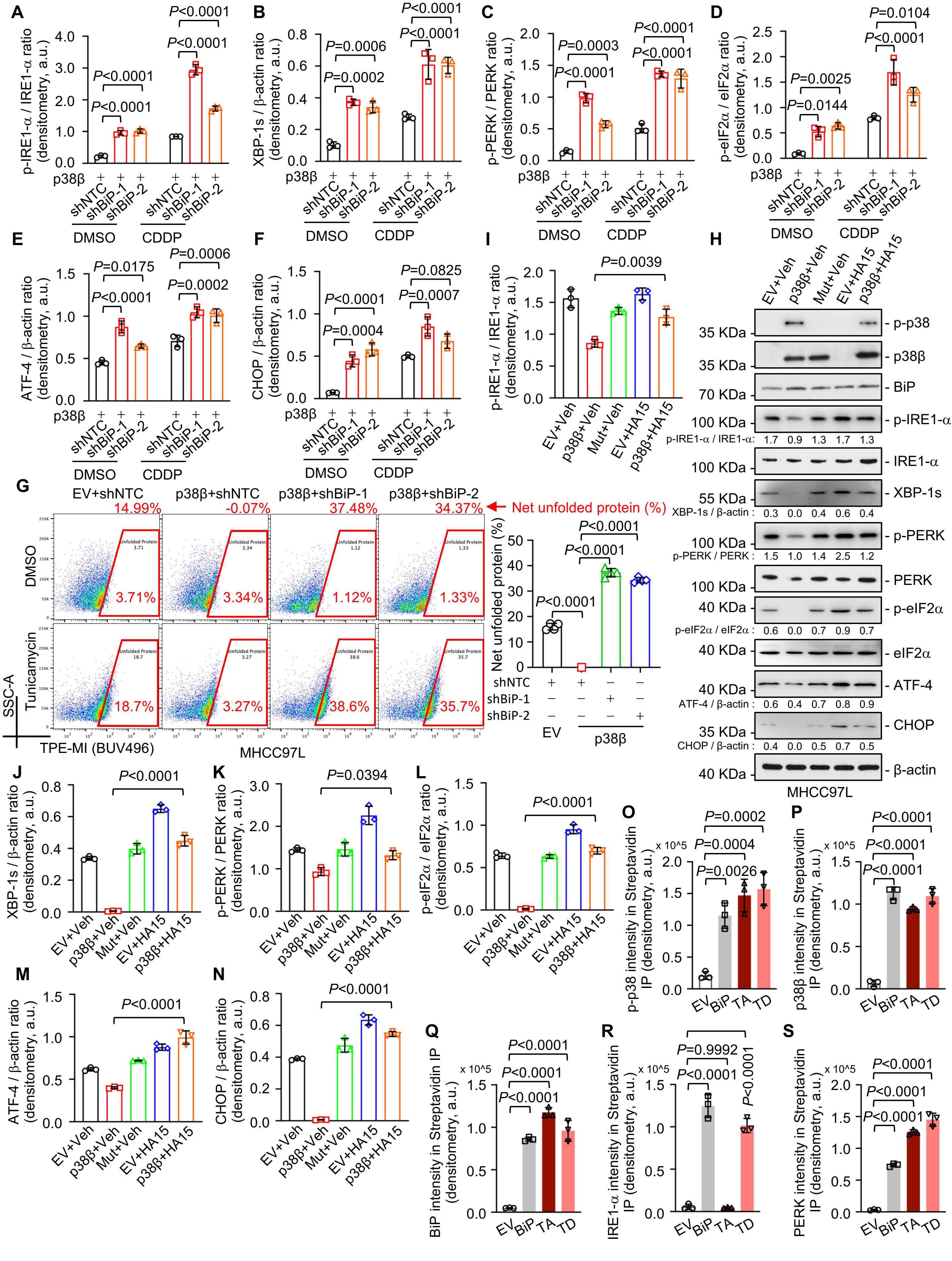

A
p-IRE1-α / IRE1-α ratio (densitometry, a.u.)
P<0.0001
P<0.0001
P<0.0001
P<0.0001
B
XBP-1s / β-actin ratio (densitometry, a.u.)
P=0.0006
P=0.0002
P<0.0001
P<0.0001
C
p-PERK / PERK ratio (densitometry, a.u.)
P=0.0003
P<0.0001
P<0.0001
P<0.0001
D
p-eIF2α / eIF2α ratio (densitometry, a.u.)
P=0.0025
P=0.0144
P=0.0104
P<0.0001
p38β
shNTC
shBiP-1
shBiP-2
DMSO
CDDP
E
ATF-4 / β-actin ratio (densitometry, a.u.)
P=0.0175
P<0.0001
P=0.0006
P=0.0002
F
CHOP / β-actin ratio (densitometry, a.u.)
P<0.0001
P=0.0004
P=0.0825
P=0.0007
I
p-IRE1-α / IRE1-α ratio (densitometry, a.u.)
P=0.0039
EV+Veh
p38β+Veh
Mut+Veh
EV+HA15
p38β+HA15
H
EV+Veh
p38β+Veh
Mut+Veh
EV+HA15
p38β+HA15
35 KDa - p-p38
35 KDa - p38β
70 KDa - BiP
100 KDa - p-IRE1-α
p-IRE1-α / IRE1-α: 1.7 0.9 1.3 1.7 1.3
100 KDa - IRE1-α
55 KDa - XBP-1s
XBP-1s / β-actin : 0.3 0.0 0.4 0.6 0.4
100 KDa - p-PERK
p-PERK / PERK : 1.5 1.0 1.4 2.5 1.2
100 KDa - PERK
40 KDa - p-eIF2α
p-eIF2α / eIF2α : 0.6 0.0 0.7 0.9 0.7
40 KDa - eIF2α
40 KDa - ATF-4
ATF-4 / β-actin : 0.6 0.4 0.7 0.8 0.9
35 KDa - CHOP
CHOP / β-actin : 0.4 0.0 0.5 0.7 0.5
40 KDa - β-actin
MHCC97L
G
EV+shNTC
p38β+shNTC
p38β+shBiP-1
p38β+shBiP-2
14.99%
-0.07%
37.48%
34.37%
Net unfolded protein (%)
DMSO
3.71%
3.34%
1.12%
1.33%
Tunicamycin
18.7%
3.27%
38.6%
35.7%
SSC-A
TPE-MI (BUV496)
MHCC97L
Net unfolded protein (%)
P<0.0001
P<0.0001
P<0.0001
shNTC
shBiP-1
shBiP-2
EV
p38β
J
XBP-1s / β-actin ratio (densitometry, a.u.)
P<0.0001
K
p-PERK / PERK ratio (densitometry, a.u.)
P=0.0394
L
p-eIF2α / eIF2α ratio (densitometry, a.u.)
P<0.0001
O
p-p38 intensity in Streptavidin IP (densitometry, a.u.)
x 10^5
P=0.0002
P=0.0004
P=0.0026
P
p38β intensity in Streptavidin IP (densitometry, a.u.)
x 10^5
P<0.0001
P<0.0001
P<0.0001
M
ATF-4 / β-actin ratio (densitometry, a.u.)
P<0.0001
N
CHOP / β-actin ratio (densitometry, a.u.)
P<0.0001
Q
BiP intensity in Streptavidin IP (densitometry, a.u.)
x 10^5
P<0.0001
P<0.0001
P<0.0001
R
IRE1-α intensity in Streptavidin IP (densitometry, a.u.)
x 10^5
P=0.9992
P<0.0001
P<0.0001
S
PERK intensity in Streptavidin IP (densitometry, a.u.)
x 10^5
P<0.0001
P<0.0001
P<0.0001
EV
BiP
TA
TD

Supplementary Figure 10. p38β-driven stemness and drug resistance via BiP-dependent UPR signaling in HCC. **A-F** Densitometric quantification of western blot bands shown in Figure 5E, p-IRE1-α was normalized to total IRE1-α (**A**), XBP-1s to β-actin (**B**), p-PERK to total PERK (**C**), p-eIF2α to total eIF2α (**D**), ATF-4 to β-actin (**E**), and CHOP to β-actin (**F**). Values are expressed relative to the control group. **G** Flow cytometry analysis using TPE-MI to assess unfolded protein accumulation in p38β-overexpressing MHCC97L cells with or without BiP knockdown, following DMSO or Tunicamycin (0.5μg/mL, 24 h) treatment. Right, quantification of net unfolded protein (%). (n = 4 independent experiments). **H-N** Western blot analysis of UPR pathway activation in p38β-overexpressing MHCC97L cells treated with or without HA15 (5 μM, 24 h) (**H**), together with corresponding densitometric quantification. The samples derive from the same experiment, but separate gels for indicated proteins were processed in parallel. p-IRE1-α was normalized to total IRE1-α (**I**), XBP-1s to β-actin (**J**), p-PERK to total PERK (**K**), p-eIF2α to total eIF2α (**L**), ATF-4 to β-actin (**M**), and CHOP to β-actin (**N**). Values are expressed relative to the control group. **O-S** Densitometric quantification of the streptavidin pull-down assays shown in Figure 5G.

Data represent three independent experiments (**H**). Values are mean ± SD (**A-G, I-S**). One-way ANOVA followed by Tukey's multiple comparison (**A-G, I-S**).

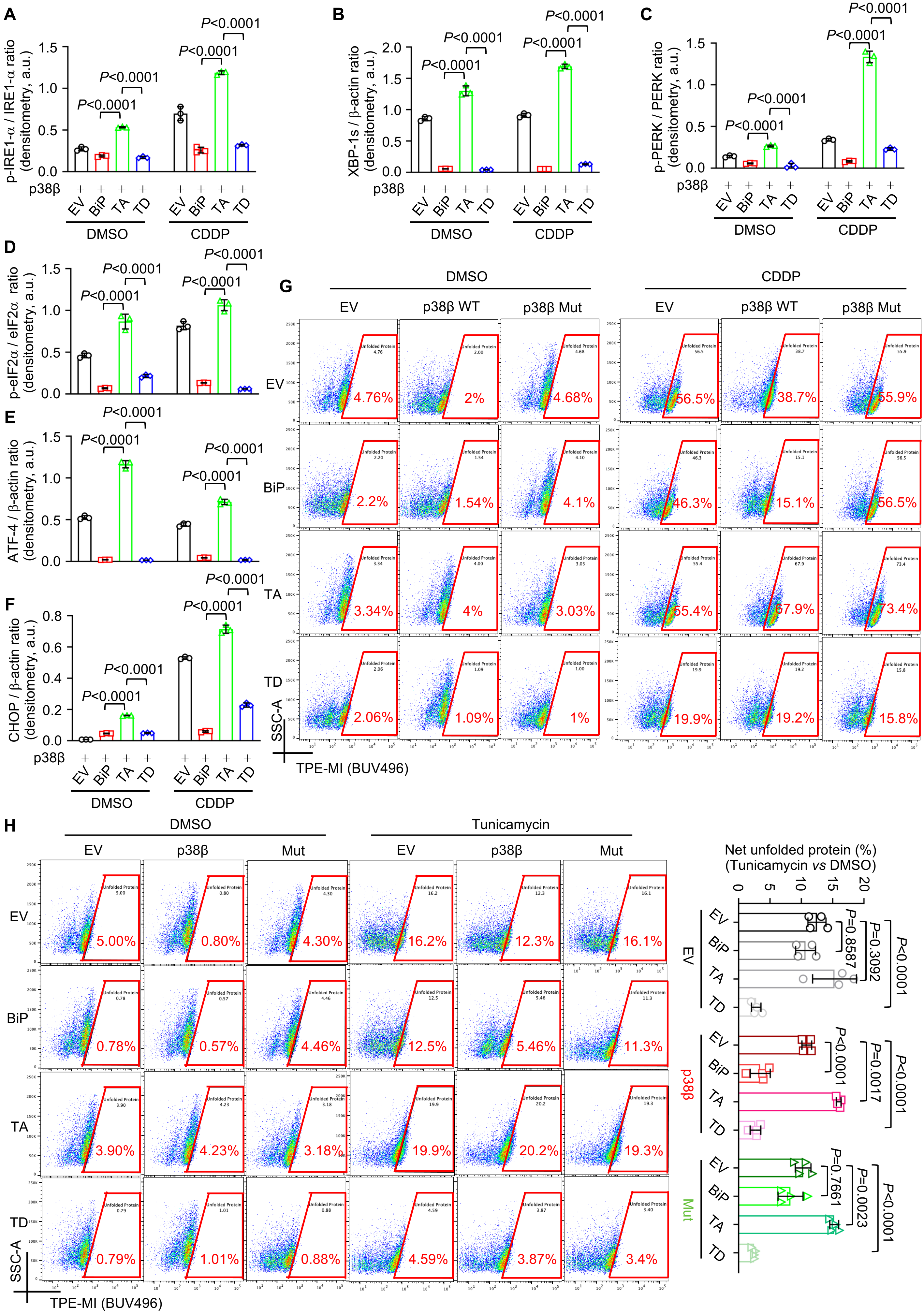

A
p-IRE1-α / IRE1-α ratio (densitometry, a.u.)
B
XBP-1s / β-actin ratio (densitometry, a.u.)
C
p-PERK / PERK ratio (densitometry, a.u.)
D
p-eIF2α / eIF2α ratio (densitometry, a.u.)
E
ATF-4 / β-actin ratio (densitometry, a.u.)
F
CHOP / β-actin ratio (densitometry, a.u.)
P<0.0001
p38β
EV
BiP
TA
TD
DMSO
CDDP
G
DMSO
CDDP
EV
p38β WT
p38β Mut
Unfolded Protein
4.76%
2%
4.68%
56.5%
38.7%
55.9%
2.2%
1.54%
4.1%
46.3%
15.1%
56.5%
3.34%
4%
3.03%
55.4%
67.9%
73.4%
2.06%
1.09%
1%
19.9%
19.2%
15.8%
SSC-A
TPE-MI (BUV496)
H
DMSO
Tunicamycin
EV
p38β
Mut
5.00%
0.80%
4.30%
16.2%
12.3%
16.1%
0.78%
0.57%
4.46%
12.5%
5.46%
11.3%
3.90%
4.23%
3.18%
19.9%
20.2%
19.3%
0.79%
1.01%
0.88%
4.59%
3.87%
3.4%
SSC-A
TPE-MI (BUV496)
Net unfolded protein (%)
(Tunicamycin vs DMSO)
P=0.8587
P=0.3092
P<0.0001
P<0.0001
P=0.0017
P<0.0001
P=0.7661
P=0.0023
P<0.0001

Supplementary Figure 11. BiP Thr648 phosphorylation suppresses UPR activation and unfolded protein accumulation. A-F Densitometric quantification of western blot bands shown in Figure 5H, p-IRE1-α was normalized to total IRE1-α (**A**), XBP-1s to β-actin (**B**), p-PERK to total PERK (**C**), p-eIF2α to total eIF2α (**D**), ATF-4 to β-actin (**E**), and CHOP to β-actin (**F**). Values are expressed relative to the control group. **G, H** Flow cytometry analysis using TPE-MI to assess unfolded protein accumulation in MHCC97L cells expressing EV, p38β WT, or Mut, each co-expressing either EV, BiP, TA, or TD, following treatment with DMSO or CDDP (10 μM, 24 h) (**G**), or with DMSO or tunicamycin (0.5μg/mL, 24 h) (**H**). Right (**H**), quantification of net unfolded protein (%). (n = 4 independent experiments).

Data are presented as mean ± SD (**A-F, H**). One-way ANOVA followed by Tukey's multiple comparison (**A-F, H**).

A

Cell number	Tumorsphere Incidence					Confidence intervals			Likelihood-ratio test P-values
	1000	500	250	125	62.5	Lower	Estimate	Upper	
Veh	10 / 12	11 / 12	7 / 12	6 / 12	1 / 12	1 / 469	1 / 318	1 / 216	
HA15	11 / 12	11 / 12	8 / 12	8 / 12	1 / 12	1 / 353	1 /239	1 / 162	0.0101
CDDP	8 / 12	9 / 12	6 / 12	4 / 12	3 / 12	1 / 705	1 / 471	1 / 315	
HA15+CDDP	4 / 12	2 / 12	1 / 12	3 / 12	0 / 12	1 / 3803	1 / 2025	1 / 1079	

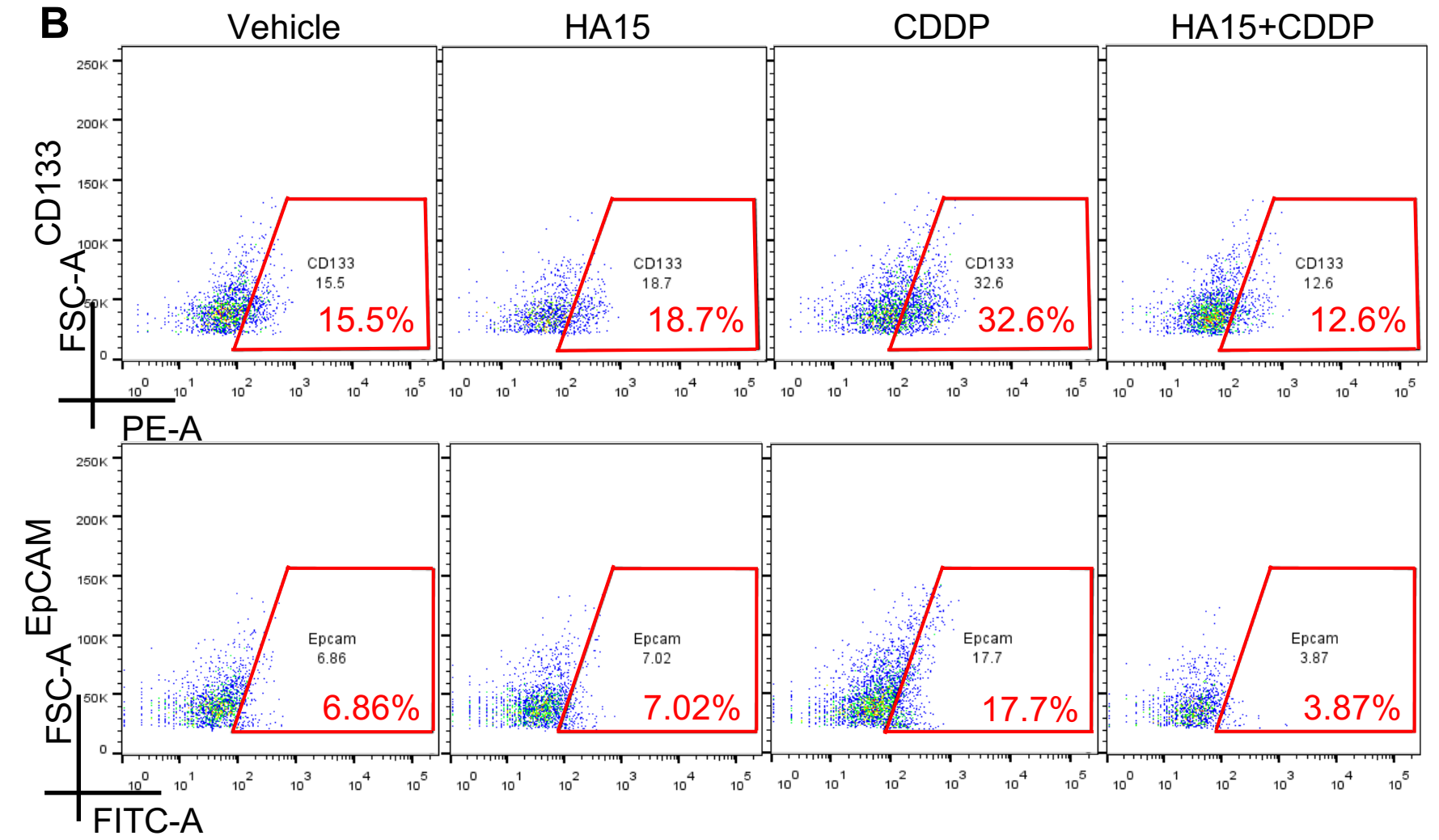

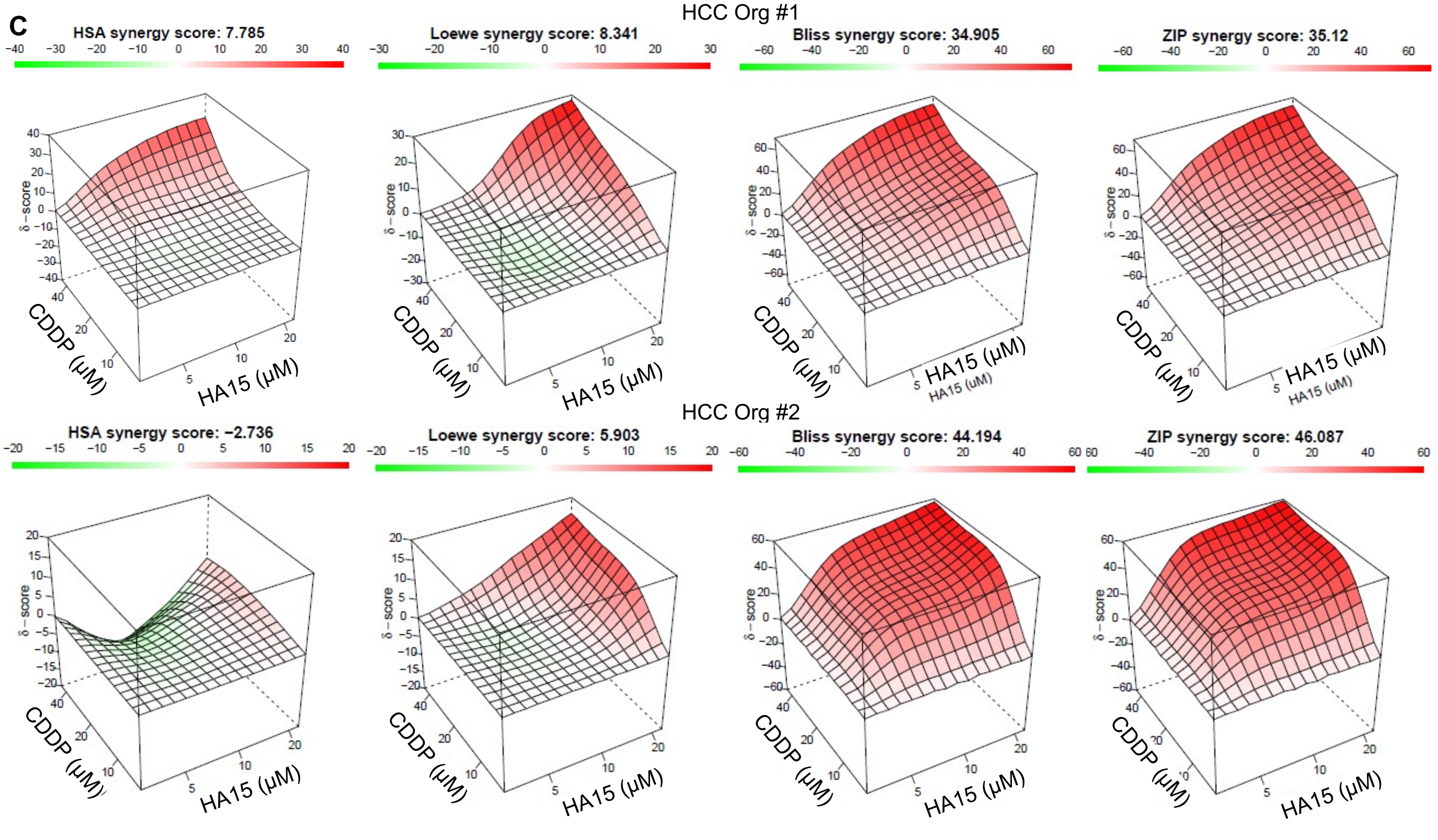

Supplementary Figure 12. HA15 treatment enhances CDDP sensitivity in PDX and PDO models. A ELDA of tumorsphere formation showing estimated stem-cell frequency (1/x) with 95% confidence intervals and goodness-of-fit statistics in each group of PDX tumour-dissociated cells. **B** Flow cytometry analysis of CSC-associated marker (CD133 and EPCAM) in each group of PDX tumour-dissociated cells. **C** 3D synergy surface plots illustrating the combinatorial effects of HA15 and CDDP in PDOs. 3D synergy maps and synergy scores were calculated with SynergyFinder (version 3) platform (https://synergyfinder.fimm.fi).

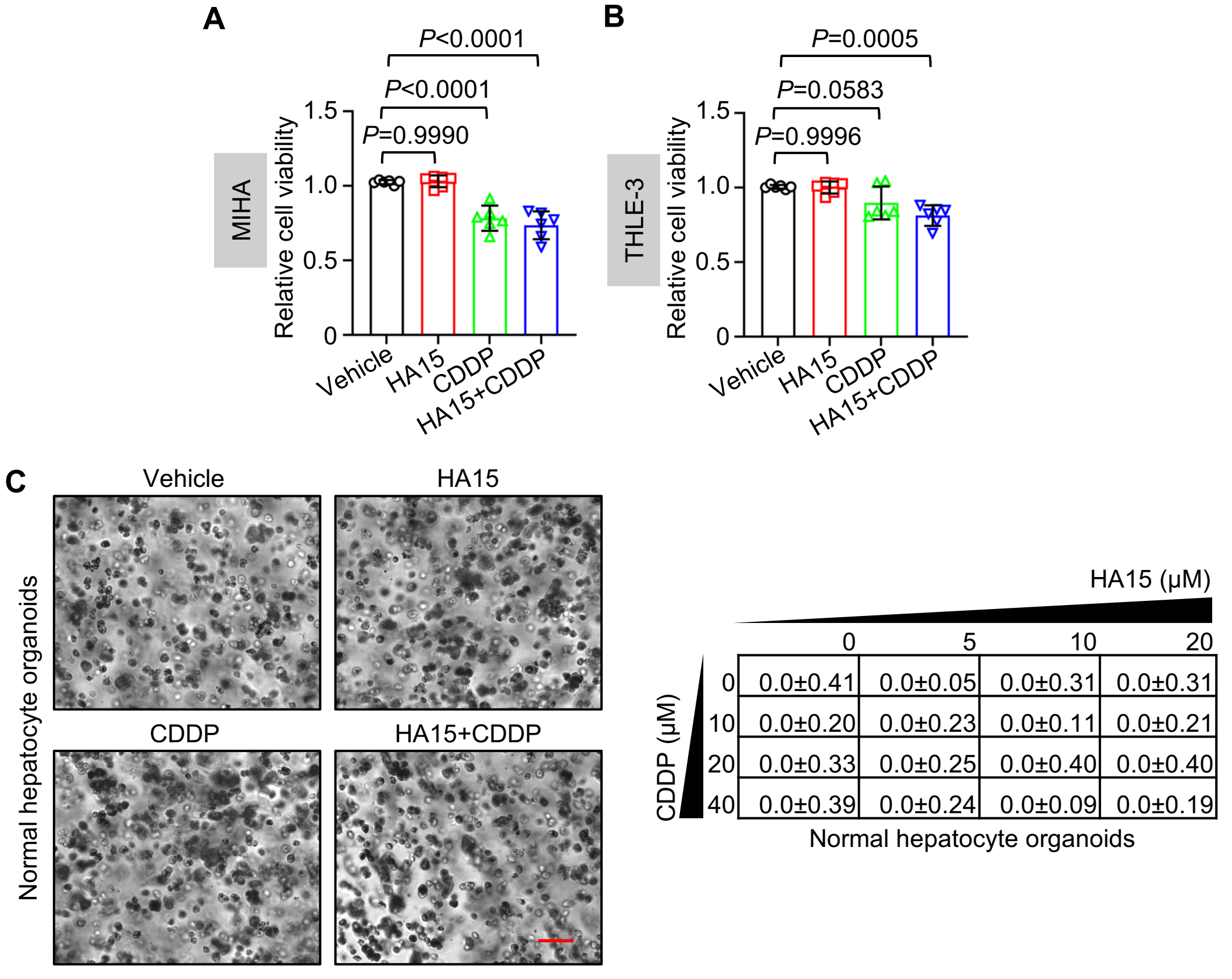

CDDP (μM) \ HA15 (μM)	0	5	10	20
0	0.0±0.41	0.0±0.05	0.0±0.31	0.0±0.31
10	0.0±0.20	0.0±0.23	0.0±0.11	0.0±0.21
20	0.0±0.33	0.0±0.25	0.0±0.40	0.0±0.40
40	0.0±0.39	0.0±0.24	0.0±0.09	0.0±0.19

Supplementary Figure 13. HA15 and CDDP treatment did not cause cytotoxicity to normal heaptocytes. A, B Cell viability was measured by XTT assay in normal liver cell lines MIHA (**A**) and THLE-3 (**B**) following treatment with vehicle, HA15 (5 µM, 24 hours), CDDP (10 µM, 24 hours), or the HA15+CDDP combination. (n = 6 independent experiments) **C** Representative images of normal human hepatocyte-derived organoids treated with vehicle, HA15, CDDP, or the combination of HA15 and CDDP. Left, Representative organoid images. Right, raw dose–response matrix data. Scale bar, 50 µm; n = 6 independent experiments.

Data are presented as mean ± SD (**A**, **B**). One-way ANOVA followed by Tukey's multiple comparison (**A**, **B**).

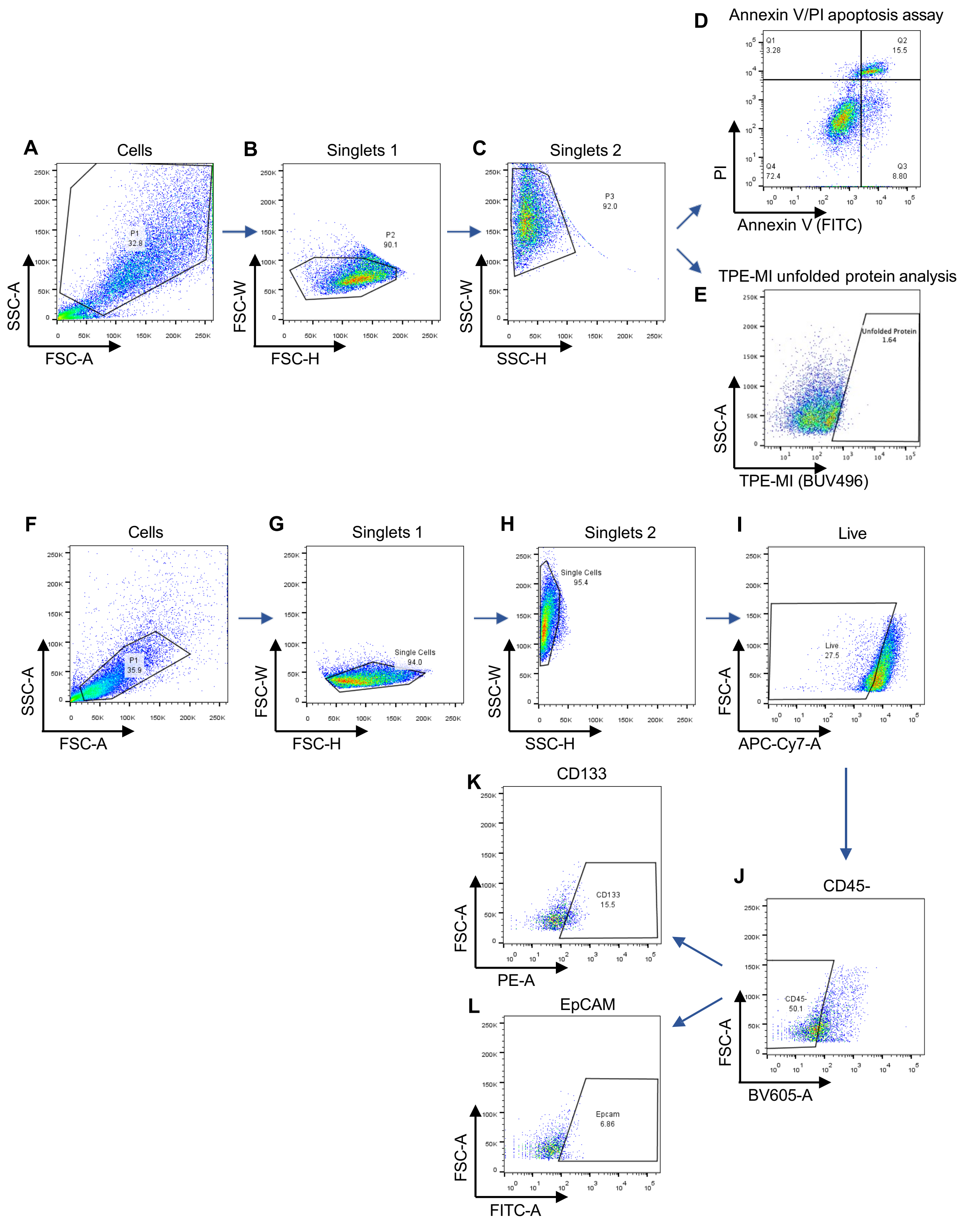

Supplementary Figure 14. Representative FACS gating strategies. A-E Representative gating strategies for the Annexin V/PI apoptosis assay and TPE-MI unfolded protein analysis. Cells were gated by FSC/SSC (**A**), followed by sequential singlet discrimination (**B, C**), and subsequent Annexin V/PI apoptosis analysis (**D**) or TPE-MI unfolded protein analysis (**E**). These gating strategies correspond to the FACS data shown in Figure 1N, 3K, 5C, 5D, 5F and Supplementary Figure 3A, 6D, 8C, 9T, 10G, 11G, 11H. **F-L**, Representative gating strategies for live EpCAM$^+$ and CD133$^+$ cells. Cells were sequentially gated by FSC/SSC (**F**), FSC-A versus FSC-H (**G**), and FSC-H versus FSC-W (**H**). Live cells (LIVE/DEAD$^-$) were selected (**I**), followed by gating of CD45$^-$ cells (**J**) and direct identification of CD133$^+$ (**K**) and EpCAM$^+$ (**L**) populations. These gating strategies correspond to the FACS data shown in Supplementary Figure 12B.
