# Supplementary material for: p38β-mediated BiP phosphorylation drives stemness and chemoresistance by suppressing UPR activation in hepatocellular carcinoma
Source: Nat Commun. 2026 Aug 5;17:9415. doi: 10.1038/s41467-026-76073-7 (PMC13538570; doi:10.1038/s41467-026-76073-7)
Supplement: Supplementary file 2 [file 41467_2026_76073_MOESM2_ESM.pdf]

Description of Additional Supplementary Files

Supplementary Data 1. Correlation of p38β expression with clinicopathological characteristics in a cohort of 136 HCC patients.

Supplementary Data 2. List of p38β-immunoprecipited proteins identified by LC–MS/MS.

Supplementary Data 3. BiP phosphorylation sites identified by LC–MS/MS.

Supplementary Data 4. Clinicopathological characteristics of the HCC PDX donor patients.

Supplementary Data 5. List of reagents and resources.

Supplementary Data 6. List of siRNAs.
