# Supplementary material for: p38β-mediated BiP phosphorylation drives stemness and chemoresistance by suppressing UPR activation in hepatocellular carcinoma
Source: Nat Commun. 2026 Aug 5;17:9415. doi: 10.1038/s41467-026-76073-7 (PMC13538570; doi:10.1038/s41467-026-76073-7)
Supplement: Supplementary file 3 [file 41467_2026_76073_MOESM4_ESM.pdf]

nature portfolio

Corresponding author(s): Man Tong

Last updated by author(s): Jun 17, 2026

Reporting Summary

Nature Portfolio wishes to improve the reproducibility of the work that we publish. This form provides structure for consistency and transparency in reporting. For further information on Nature Portfolio policies, see our Editorial Policies and the Editorial Policy Checklist.

Statistics

For all statistical analyses, confirm that the following items are present in the figure legend, table legend, main text, or Methods section.

n/a	Confirmed	
☐	☒	The exact sample size (*n*) for each experimental group/condition, given as a discrete number and unit of measurement
☐	☒	A statement on whether measurements were taken from distinct samples or whether the same sample was measured repeatedly
☐	☒	The statistical test(s) used AND whether they are one- or two-sided<br>*Only common tests should be described solely by name; describe more complex techniques in the Methods section.*
☒	☐	A description of all covariates tested
☐	☒	A description of any assumptions or corrections, such as tests of normality and adjustment for multiple comparisons
☐	☒	A full description of the statistical parameters including central tendency (e.g. means) or other basic estimates (e.g. regression coefficient) AND variation (e.g. standard deviation) or associated estimates of uncertainty (e.g. confidence intervals)
☐	☒	For null hypothesis testing, the test statistic (e.g. *F*, *t*, *r*) with confidence intervals, effect sizes, degrees of freedom and *P* value noted<br>*Give P values as exact values whenever suitable.*
☒	☐	For Bayesian analysis, information on the choice of priors and Markov chain Monte Carlo settings
☒	☐	For hierarchical and complex designs, identification of the appropriate level for tests and full reporting of outcomes
☐	☒	Estimates of effect sizes (e.g. Cohen's *d*, Pearson's *r*), indicating how they were calculated

Our web collection on statistics for biologists contains articles on many of the points above.

Software and code

Policy information about availability of computer code

Data collection	BD LSRFortessa, BD FACSAria II and FACSDiva software (BD Biosciences) was used to collect flow cytometry data.<br>LightCycler480 software (version 1.5.1.62) was used to collect qPCR data.<br>BioRad ImageLab Touch Software (version 2.4.0.03) was used to collect Western Blot images.<br>ZEISS Axioscan 7 Microscope Slide Scannerwas used to collect immunohistochemistry images.<br>Orbitrap Fusion Tribrid Lumos mass spectrometer (Thermo Fisher Scientific) were used for phosphoproteomic MS analysis.
Data analysis	FlowJo (version 10) was used for analysis of flow cytometry data.<br>LightCycler480 software (version 1.5.1.62) was used for analysis of qPCR data.<br>ImageJ software (version 1.53t) was used to analyze foci and immunohistochemistry images.<br>MaxQuant (version 1.6.0.13) was used for analysis of phosphoproteomic MS data.<br>Stem cell frequency was estimated using ELDA software (https://bioinf.wehi.edu.au/software/elda/).<br>GSEA (version 4.3.2) was used for GSEA experiments.<br>Synergy scores were calculated using SynergyFinder (version 3).<br>GraphPad Prism (version 10.1.2) and SPSS software (version 21.0) were used for statistical analyses.

For manuscripts utilizing custom algorithms or software that are central to the research but not yet described in published literature, software must be made available to editors and reviewers. We strongly encourage code deposition in a community repository (e.g. GitHub). See the Nature Portfolio guidelines for submitting code & software for further information.

Data

Policy information about availability of data

All manuscripts must include a data availability statement. This statement should provide the following information, where applicable:

- Accession codes, unique identifiers, or web links for publicly available datasets
- A description of any restrictions on data availability
- For clinical datasets or third party data, please ensure that the statement adheres to our policy

Data availability
The transcriptomic dataset of chemotherapy-enriched HCC spheroids used in this study is publicly available in the Gene Expression Omnibus (GEO) database under accession code GSE53005 (https://www.ncbi.nlm.nih.gov/search/all/?term=GSE53005). Transcriptomic levels of MAPK11 and stemness marker genes are available in The Cancer Genome Atlas Liver Hepatocellular Carcinoma (TCGA-LIHC). Site-specific BiP phosphorylation levels in HCC patients are available in Clinical Proteomic Tumor Analysis Consortium (CPTAC, PDC000199) (https://proteomic.datacommons.cancer.gov/pdc/study/PDC000199). All mass spectrometry datasets generated in this study, including phosphoproteomic and other proteomic analyses, have been deposited in the PRIDE repository under accession code PXD067039. All other data supporting the findings of this study, including quantitative results from functional assays, are available within the Source Data file. Source data are provided with this paper.

Research involving human participants, their data, or biological material

Policy information about studies with human participants or human data. See also policy information about sex, gender (identity/presentation), and sexual orientation and race, ethnicity and racism.

Reporting on sex and gender	For tissue microarray of human HCC samples, sex and gender were not considered in this study. This study involves 110 males and 26 females.
Reporting on race, ethnicity, or other socially relevant groupings	For tissue microarray of human HCC samples, race and ethnicity were not considered in this study. Such information is not available.
Population characteristics	136 Primary HCC patients underwent surgery with regular follow-up. Other treatment information is not available.
Recruitment	Primary HCC patients were recuited by Sun Yat-sen University Cancer Center in Gua.ngzhou, China. There is no potential bias for recruiting patient cohort. 136 archived paraffin-embedded pathological specimens from primary HCC patients were collected along with complete clinical and pathological data at the Sun Yat-sen University Cancer Center. All samples were anonymous.
Ethics oversight	Tissue microarray (TMA) was obtained from Professor Jing-Ping Yun at the Sun Yat-sen University Cancer Centre in Guangzhou, China, with the approval of the Institutional Review Board for ethical review from the Sun Yat-sen University in Guangzhou, China with written informed consent from all patients and no financial compensation provided.

Note that full information on the approval of the study protocol must also be provided in the manuscript.

Field-specific reporting

Please select the one below that is the best fit for your research. If you are not sure, read the appropriate sections before making your selection.

☒ Life sciences ☐ Behavioural & social sciences ☐ Ecological, evolutionary & environmental sciences

For a reference copy of the document with all sections, see nature.com/documents/nr-reporting-summary-flat.pdf

Life sciences study design

All studies must disclose on these points even when the disclosure is negative.

Sample size	We did not perform sample size calculations and sample sizes were chosen according to accepted standards in the field of study (Naegle K, Gough NR, Yaffe MB. Criteria for biological reproducibility: what does "n" mean? Sci Signal. 2015 Apr 7;8(371):fs7. doi: 10.1126/scisignal.aab1125.). Individual data points from biological replicates were shown in each figure. For animal studies, preliminary experiments were performed to determine the variation in growth rate and response to treatment.
Data exclusions	No data was excluded from this study.
Replication	The number of biological replicates and independent experiments are stated in the figure legend.
Randomization	All mice were randomly assigned to treatment groups.
Blinding	The investigators were not blinded to group allocation, data collection or data analysis because the investigators were responsible for

Blinding	performing the experiment, collecting and labelling the samples, and analysis of data. For analysis of sequencing data, blinding was not necessary. We have planned the analysis pipeline before the acquisition of sequencing data.

Reporting for specific materials, systems and methods

We require information from authors about some types of materials, experimental systems and methods used in many studies. Here, indicate whether each material, system or method listed is relevant to your study. If you are not sure if a list item applies to your research, read the appropriate section before selecting a response.

Materials & experimental systems

n/a	Involved in the study
☐	☒ Antibodies
☐	☒ Eukaryotic cell lines
☒	☐ Palaeontology and archaeology
☐	☒ Animals and other organisms
☐	☒ Clinical data
☒	☐ Dual use research of concern
☒	☐ Plants

Methods

n/a	Involved in the study
☒	☐ ChIP-seq
☐	☒ Flow cytometry
☒	☐ MRI-based neuroimaging

Antibodies

Antibodies used	MAPK11/p38β rabbit pAb, 1:100, Atlas Antibodies, Cat#HPA045069; RRID:AB_2679207<br>MAPK11/p38β rabbit mAb, 1:1000, CST,Cat#2339; RRID:AB_823587<br>Phospho-p38 (Thr180/Try182) rabbit pAb, 1:1000, CST, Cat#9211; RRID:AB_331641<br>GRP78 (BiP) rabbit pAb, 1:1000, Proteintech, Cat#11587-1-AP; AB_2119855<br>IRE1-α rabbit mAb, 1:1000, CST, Cat#3294; RRID:AB_823545<br>PERK rabbit mAb, 1:1000, CST, Cat#5683; RRID:AB_10841299<br>Phospho-IRE1-α (Ser724) rabbit pAb, 1:1000, Sigma-Aldrich, Cat#SAB5700519; RRID:AB_3697855<br>Phospho-PERK (Thr980) rabbit mAb, 1:1000, CST, Cat#3179; RRID:AB_2095853<br>PCNA mouse mAb, 1:100, Abcam, Cat#Ab29; RRID:AB_303394<br>Beta Actin (β-actin) mouse mAb, 1:3000,Sigma-Aldrich, Cat#A5316; RRID:AB_476743<br>Goat anti-mouse IgG pAb, 1:100, HRP, Sigma-Aldrich, Cat#A4416; RRID:AB_258167<br>Donkey anti-rabbit IgG pAb, 1:100, HRP, Cytiva, Cat#NA934; RRID:AB_772206<br>MAPK12 rabbit pAb, 1:1000, Proteintech, Cat#20184-1-AP; RRID:AB_10665949<br>MAPK13 Mouse mAb, 1:1000, Proteintech, Cat#60252-1-Ig; RRID:AB_2881373<br>MAPK14 rabbit pAb, 1:1000, CST, Cat#9218; RRID:AB_10694846<br>HSPA8/Hsc70 rabbit mAb, 1:1000, Abcam, Cat#Ab51052; RRID: AB_880538<br>ATF-4 Rabbit mAb, 1:1000, CST, Cat#11815; RRID:AB_2616025<br>Phospho-Threonine (42H4) Mouse mAb, 1:1000, CST, Cat#9386; RRID:AB_331239<br>Phospho-eIF2 alpha rabbit mAb, 1:1000, CST, Cat#3398; RRID:AB_2096481<br>eIF2 alpha rabbit mAb, 1:1000, CST, Cat#5324; RRID:AB_10692650<br>CHOP Mouse mAb,1:500, CST, Cat#2895; RRID:AB_2089254<br>GRP78 (BiP) Mouse mAb, 1:1000, Proteintech, Cat#66574-1-Ig; RRID: AB_2881934<br>Mouse Control IgG, 1:10000, ABclonal, Cat#AC011; RRID:AB_2770414<br>Rabbit Control IgG, 1:5000, ABclonal, Cat#AC005; RRID:AB_2771930
Validation	All antibodies are validated by the manufacturer as suitable for the applications. The product size in Western Blot experiments were confirmed by comparison to protein size ladder.

Eukaryotic cell lines

Policy information about cell lines and Sex and Gender in Research

Cell line source(s)	Human cell lines HepG2 (HB-8065), Hep3B (HB-8064) and HEK293T (CRL-3216) were obtained from the American Type Culture Collection (ATCC). HCC cell lines HuH-1 and Huh-7 were obtained from Japanese Cancer Research Resources Bank (JCRB). HCC cell lines MHCC97L and MHCC97H, as well as the immortalized normal liver cell line MIHA, were generously provided by the Liver Cancer Institute of Fudan University. Normal liver cell line THLE-3 was purchased from ATCC. HCC cell line PLC8024 was obtained from the Institute of Virology, Chinese Academy of Medical Sciences (Beijing, China).
Authentication	All cell lines were authenticated by morphological observation and STR profiling.
Mycoplasma contamination	All cell lines were negatively for mycoplasma contamination and routinely tested for microplasma using PCR assay.
Commonly misidentified lines<br>(See ICLAC register)	No commonly misidentified cell lines were used.

Animals and other research organisms

Policy information about studies involving animals; ARRIVE guidelines recommended for reporting animal research, and Sex and Gender in Research

Laboratory animals	Mouse (Mus musculus), NOD/SCID, 4 to 6 weeks.
Wild animals	No wild animals were used in the study.
Reporting on sex	Male Mouse(Mus musculus), NOD/SCID, 4 to 6 weeks.
Field-collected samples	No field collected samples were used in the study.
Ethics oversight	All animal experiments were approved by and conducted in accordance with the Hong Kong Animals (Control of Experiments) Ordinance, the Committee on the Use of Live Animals in Teaching and Research at The University of Hong Kong (Approval No. 4012-16), and the Animal Experimentation Ethics Committee of The Chinese University of Hong Kong (Approval No. 22-195-GRF).

Note that full information on the approval of the study protocol must also be provided in the manuscript.

Clinical data

Policy information about clinical studies

All manuscripts should comply with the ICMJE guidelines for publication of clinical research and a completed CONSORT checklist must be included with all submissions.

Clinical trial registration	N.A.
Study protocol	N.A.
Data collection	N.A.
Outcomes	N.A.

Plants

Seed stocks	N.A.
Novel plant genotypes	N.A.
Authentication	N.A.

Flow Cytometry

Plots

Confirm that:

☒ The axis labels state the marker and fluorochrome used (e.g. CD4-FITC).

☒ The axis scales are clearly visible. Include numbers along axes only for bottom left plot of group (a 'group' is an analysis of identical markers).

☒ All plots are contour plots with outliers or pseudocolor plots.

☒ A numerical value for number of cells or percentage (with statistics) is provided.

Methodology

Sample preparation	HCC cells were harvested by trypsin and stained with Annexin V-FITC and propidium iodide, or TPE-MI.
Instrument	BD LSRFortessa and BD FACSAria II.
Software	FlowJo (version 10)

Cell population abundance	The final target population consists of at least 10,000 cells.
Gating strategy	The total population was visualized using FSC/SSC graph, and the main population was gated. Subsequently, singlets were gated using FSC-H/FSC-A graph. The singlets were then used for analysis.

☐ Tick this box to confirm that a figure exemplifying the gating strategy is provided in the Supplementary Information.
