# Supplementary material for: p38β-mediated BiP phosphorylation drives stemness and chemoresistance by suppressing UPR activation in hepatocellular carcinoma
Source: Nat Commun. 2026 Aug 5;17:9415. doi: 10.1038/s41467-026-76073-7 (PMC13538570; doi:10.1038/s41467-026-76073-7)
Supplement: Supplementary file 4 [file 41467_2026_76073_MOESM5_ESM.pdf]

nature portfolio

Peer Review File

p38β-mediated BiP phosphorylation drives stemness and chemoresistance by suppressing UPR activation in hepatocellular carcinoma

Corresponding Author: Dr Man Tong

This file contains all reviewer reports in order by version, followed by all author rebuttals in order by version.

Version 0:

Reviewer comments:

Reviewer #1

(Remarks to the Author)
This paper reports that expression of the stress kinase p38β is increased in hepatocellular carcinoma (HCC) spheroids cultured with cisplatin and doxorubicin. Moreover, high p38β expression correlates with poor prognosis and aggressive clinical features in HCC patients. The downregulation of p38β in a HCC cell line that expresses high levels of this kinase reduces its foci forming ability and increases cisplatin-induced cell death. Conversely, overexpression of p38β WT, but not of a non-phosphorylatable mutant, in a HCC cell line that expresses low levels of the kinase improves foci formation and the ability to form tumors in mice. The ER chaperone BiP is found to interact with p38β, and shRNA-mediated BiP downregulation reverts the reduced death observed in cisplatin-treated cells upon p38β overexpression. Phosphoproteomic analyses detect an enhanced phosphorylation of BiP on Thr648 in p38β overexpressing cells. The overexpression of p38β facilitates the co-immunoprecipitation of BiP with the UPR sensors/activators IRE-1a and PERK. Overexpression of BiP mutants shows that mutation of Thr648 to Ala impairs the interaction with IRE-1a. In tunicamycin-treated cells, overexpression of p38β or a BiP mutant with Thr648 mutated to Glu, but not to Ala, reduces unfolded protein accumulation, whereas downregulation of p38β or BiP increases unfolded protein accumulation. Finally, pharmacological inhibition of BiP using HA15 potentiates the cisplatin activity in suppressing the growth of a patient-derived xenograft and two organoid models, which correlates with increased detection of phospho-IRE-1a signal in tumors.

The manuscript contains a substantial amount of data, the narrative is appealing and some observations are consistent with the conclusions but several key claims are not really demonstrated. The proposal that p38β contributes to stemness and chemoresistance to cisplatin in HCC is a new and interesting finding, which could potentially have therapeutic implications. Evidence is presented that this function of p38β is somewhat mediated by BiP. The results support the idea that BiP phosphorylation on Thr648 regulates the UPR, probably by facilitating its interaction with some UPR sensors/activators, and contributes to cancer cell stemness or survival, but the link with p38β is very weak. The fact that a BiP protein with Thr648 mutated to Ala interferes with some effects of p38β overexpression does not necessarily mean that p38β regulates Thr648 phosphorylation.

It is also difficult to know what was actually done in several experiments due to the scarcity of information provided. This includes the initial analysis that led to the identification of p38β as a potentially regulator of HCC, the proteomic and phospho-proteomic experiments to identify p38β-BiP interaction and BiP phosphorylation, and the generation of the PDX and PDO models used. Important controls are missing in some experiments. Overall, the results are not as conclusive as the authors seem to believe, and without further experimental support it is unclear whether the authors might be correct in their conclusions.

Below are specific comments and points that should be addressed to provide support for the authors' conclusions in a more convincing manner.

1. The experimental pipeline that led to the identification of p38β is not properly described. Fig. 1A indicates the use of cDNA microarrays, but there is no description of the HCC spheroids used and how they were treated, the type and processing of the microarrays, the samples that were compared or how the analysis was performed to select 31 candidates. Results of the microarray should be presented as supplementary material, and raw data deposited in a database. The

analysis of the DEMETER2 dataset that led to the identification of 37 candidates should be explained. The criteria to rank p38β as #1 should be also described. As it stands, it is not clear why p38β was selected for further study.
In this line, the references to “kinome-wide screen” and “kinase dependency” in Abstract and Discussion are misleading as no functional assays were apparently done for the selection of p38β.

2. The experiments that investigate the effect of p38β downregulation are important and it is critical to show in all these experiments a WB of p38a to confirm the specificity of the reagents used. This includes Fig. 1H and Suppl. 4A.

3. Fig. 1K. The raw data in the image (left) do not correlate with values in the histogram (right). In the raw data LacZ is higher than Parental and about the same as sg p38β-2. These results indicate that only sg p38β-1 but not sg p38β-2 has an effect.

4. The cell lines MHCC97L, which expresses low levels of p38β, and PLC8024, which expresses high levels of p38β, are used to investigate the effects of p38β overexpression and downregulation, respectively. Since only one cell line is used in each case, it would be important to show that the distinctive levels of p38β expression in MHCC97L and PLC8024 cells correlate with different stemness properties and cisplatin sensitivity in the two cell lines.

5. Fig. 2H. The phospho antibody used recognizes the two residues that are mutated in p38β, and the lack of signal in Mut is therefore expected. A WB with an antibody that recognizes a downstream substrate of p38β (i.e., MK2) would be more relevant to confirm the pathway activity status in cells that overexpress WT or mutant p38β.

6. Immunoprecipitation experiments should always include an IgG control to demonstrate the specificity of the interactions, but this is not used in any of the experiments presented. This control is especially critical in the samples that show the interaction of overexpressed p38β with other proteins, i.e., p38β-overexpressing cells in Fig 3B, 3C and 5A, as well as the patient samples of Fig. 3E.

7. There is no description of the phosphoproteomic experiment that led to the identification of increased BiP phosphorylation on Thr648 in p38β overexpressing cells. Methods describe in a very generic way how the samples were processed but the results are not presented anywhere. How many samples were analyzed, how much was the increase and compare with what? Results should be presented as supplementary material, and raw data deposited in a database.

8. The link between p38β and BiP phosphorylation on Thr648 is not convincing. There is no evidence that p38β can phosphorylates BiP, which would require performing in vitro kinase assays. Likewise, no experiment is shown to support a link between p38β expression or activity and BiP-Thr648 phosphorylation in cells. For example, is this phosphorylation reduced in cells upon p38β downregulation or increased when cells overexpress p38β? Are the BiP-Thr648 phosphorylation levels different between MHCC97L and PLC8024 cells? A phospho-Thr648 antibody would be perfect for these experiments. Alternatively, BiP immunoprecipitations followed by WB with a generic phospho-Thr antibody could be tried, or phosphoproteomic analysis.

9. If the link between BiP-Thr648 phosphorylation and p38β is confirmed, it would be important to explain how an ER-luminal protein like BiP can be phosphorylated by p38β, whose substrates are typically cytosolic or nuclear.

10. The in vivo experiment (Fig. 6) shows a moderate increase in the cisplatin effect upon combination with HA15. This effect is expected based on previous literature showing that BiP inhibition interferes with tumor growth, but the data provides support neither for the importance of BiP phosphorylation nor for the implication of p38β upstream of BiP

11. How was the PDX used in Fig. 6A-6C obtained and what are its characteristics? Same for the two PDOs used in Fig. 6I-6J.

12. Related to Fig 5C, does p38β overexpression reduce Tunicamycin-induced cell death?

13. Two different p38β antibodies are mentioned in the Reagents section, and figure legends should indicate which antibody was used in each case.

Reviewer #2

(Remarks to the Author)
Summary:
This manuscript explores how the stress-activated kinase p38β (MAPK11) may promote tumor-initiating cell (TIC) properties and chemoresistance in hepatocellular carcinoma (HCC) by modulating the unfolded protein response (UPR) through the ER chaperone BiP. A kinome-wide shRNA screen in chemotherapy-resistant spheroids identified p38β as a candidate factor, and high expression was associated with poorer survival and adverse clinicopathological features. Functional studies suggest that loss of p38β reduces colony formation, self-renewal, and cisplatin resistance, while overexpression increases TIC frequency in vitro and in xenograft models. Mechanistically, p38β appears to phosphorylate BiP at threonine 648, which may enhance BiP binding to IRE1α and PERK, thereby attenuating UPR activation and limiting stress-induced apoptosis. Mutational analysis of BiP at Thr648 analyses the functional relevance of this modification, and suggests that pharmacological inhibition of BiP synergizes with cisplatin in patient-derived xenografts and organoids.
Overall, this work reveals a novel p38β–BiP signaling axis linking ER stress adaptation to cancer stemness and chemoresistance in HCC. The experiments address an important question and are generally well-designed, and the findings

are of high interest for the HCC and cancer stem cell fields. However, there are several substantive issues regarding the strength of the evidence and the clarity of data presentation that should be addressed to solidify the conclusions. The suggested revisions are intended to improve the rigor and transparency of the study, thereby ensuring that the conclusions are fully supported by the data.

Major Comments:

1. Requirement for p38β kinase activity and on-target specificity: The conclusion that p38β's kinase activity is essential for TIC maintenance and drug resistance should be reinforced with additional controls and broader validation. The current evidence relies on overexpression of WT vs. a T180A/Y182F "kinase-dead" mutant (p38β Mut) in MHCC97L, plus one knockout model. These experiments do suggest that kinase activity is necessary – for example, Figures 2I–J show that overexpressing p38β WT significantly increases colony formation and tumorsphere frequency compared to empty vector, whereas the kinase-dead mutant fails to do so (indeed performing similarly to the control). Likewise, p38β knockout dramatically impairs oncogenic growth (Fig. 1I) and increases apoptosis under cisplatin (Fig. 1K), indicating that p38β is required for these phenotypes. While these results are compelling, it is critical to confirm that the effects are truly due to p38β's catalytic function and not off-target consequences of overexpression or genetic manipulation.

First, please ensure that the kinase-dead mutant is expressed at a comparable level to WT p38β and is indeed catalytically inactive in cells. It is implied that expression was matched (Fig. 4G confirms similar protein levels for WT and mutant), but demonstrating loss of kinase activity would strengthen confidence. A straightforward way is to examine the phosphorylation of known p38 downstream targets (e.g., MK2 or HSP27) in the WT vs. Mut overexpressors. Providing a Western blot for phospho-MK2 or phospho-HSP27 in the empty vector, WT, and Mut backgrounds would be a strong validation that the mutant truly lacks kinase function in the cellular context.

Second, the study would be strengthened by pharmacological inhibition experiments to ensure that blocking p38β activity phenocopies the genetic loss-of-function. Currently, no small-molecule p38 inhibitor data are presented. Treating p38β-overexpressing cells with a known p38α/β inhibitor (such as SB203580, or a more potent agent like BIRB796 or Ralimetinib) and showing a reversal of the TIC phenotypes would provide crucial on-target evidence. One approach is to combine inhibitor treatment with selective knockdown of p38α (MAPK14). If, in p38β-overexpressing cells, the inhibitor still reduces sphere formation or enhances cisplatin-induced cell death even after p38α is silenced, this would indicate the drug effect is likely acting through p38β. In any case, a pharmacological validation of p38β's role in maintaining the TIC phenotype would significantly bolster the conclusions.

Additionally, extending the core functional assays to a second HCC cell line would improve generalizability. The current in vitro data are largely from MHCC97L (a line with relatively low endogenous p38β). It would be informative to test p38β loss-of-function in an HCC line that naturally has high p38β expression (to see if they depend on it), or conversely to overexpress WT vs. Mut in another low-p38β line (such as Huh7 or Hep3B, if they have lower MAPK11 levels) and assess colony formation, sphere formation, and drug response.

2. Mechanistic link between p38β and BiP – biochemical evidence and specificity: The identification of BiP as a p38β-interacting protein and substrate is central to the paper's mechanism. While the data presented are intriguing, some aspects need more rigorous support to establish this interaction and modification as specific and functionally relevant.

In Figure 3A, a proteomics approach pulled down p38β and identified interactors, highlighting BiP as a top hit. However, it's not clear if this experiment was performed in replicates or how significance was determined. To bolster confidence in BiP as a bona fide interactor, the proteomic results should be quantified and presented with appropriate statistics (i.e. volcano plot) - using the intensity rather than spectral counts (#pep). BiP should clearly be labeled among the significantly enriched proteins, as well as the well-known MAPK11 partners such as MAP2K3, MKNK1 and DUSP9 (already displayed in the table, Fig 3A).

The co-immunoprecipitation experiments in Figure 3B and 3D validate that p38β and BiP form a complex in cells. One concern is whether this interaction is specific to the p38β isoform. Since the phospho-p38 antibody used recognizes both p38α and p38β when they are activated, it remains possible that p38α could also bind BiP during stress. To address this, please clarify whether p38α interacts with BiP under the same conditions. The authors could perform a parallel co-IP using p38α (MAPK14) as the target: immunoprecipitate p38α from HCC cell lysates (perhaps under stress conditions similar to those used for p38β IP) and probe for BiP. Alternatively, showing that the level of BiP co-immunoprecipitated with phospho-p38 in Figure 3D drops substantially when p38β is knocked down (leaving p38α as the main source of p-p38) would also indicate specificity. Establishing isoform specificity is important, because it underpins the idea that p38β has a unique role (as opposed to the well-studied p38α which might otherwise be assumed to perform similar functions).

Another mechanistic point is whether the p38β–BiP binding is direct or mediated by other proteins. The text implies a direct interaction, and the co-IP supports a physical association. If feasible, an in vitro binding assay (using purified p38β and BiP proteins) could be a definitive way to show direct binding. This is especially interesting because p38β is a cytosolic kinase, whereas BiP is primarily in the ER lumen; thus one might speculate that either a subset of BiP is present in the cytosol or that p38β transiently associates with the cytosolic side of the ER where it could access BiP's ATPase domain (which contains Thr648). A brief discussion of this will preempt reader curiosity on how a cytosolic kinase phosphorylates an ER luminal chaperone.

In Figures 4C and 4D, the authors present phosphoproteomic data showing that BiP is phosphorylated at Thr648 in p38β-

overexpressing cells. Figure 4C shows that the normalized abundance of the BiP pT648 peptide is markedly higher in p38β-WT cells compared to empty vector, but the overall phosphosite intensity (10E4) is very low as well as a poor MS2 spectra (Figure 4D). These data point to BiP as a substrate of p38β, but more detail on this phosphosite identification should be provided. Was Thr648 one of the top upregulated phosphosites in the entire dataset, or at least among BiP peptides? Also, were any other BiP phosphorylation sites detected, and if so, did those change with p38β overexpression? Were downstream BiP phosphorylation events also effected by Thr648? BiP has multiple known phosphosites, validation of that Thr648 was uniquely or most strongly regulated by p38β would emphasize its likely importance.

3. UPR suppression and chemoresistance phenotypes - quantification and additional readouts: A major claim of the paper is that p38β's phosphorylation of BiP dampens UPR signaling, thereby reducing ER stress levels and preventing cell death under chemotherapy. The experiments in Figure and Supplementary Fig. address this by measuring UPR sensor activation (IRE1α and PERK phosphorylation) and the accumulation of misfolded proteins (using the TPE-MI fluorescent dye) in various conditions. The trends observed are consistent with the model, but some of these effects should be quantified more rigorously and supported by additional readouts to fully convince the reader.

The most rigorous option would be to use targeted quantitative phosphoproteomics (PRM) to directly measure key UPR phosphopeptides (e.g., activation-loop sites in IRE1α and PERK). PRM would provide sensitive and precise quantification across conditions (EV/WT/Mut/KO, ± cisplatin or tunicamycin), yielding fold-changes with clear statistics. If PRM is not feasible, densitometric analysis of the existing Western blots across ≥3 independent experiments (normalising p-IRE1α and p-PERK to totals) should be performed to report mean fold-change ± SD/SEM and significance.

In addition, at least one downstream UPR effector readout (e.g., CHOP protein induction or XBP1 mRNA splicing) under stress would strengthen the mechanistic link. Reduced CHOP and less XBP1 splicing in p38β-overexpressing cells, and the opposite in p38β-KO or BiP-T648A contexts, would confirm that the biochemical suppression of UPR sensors translates into differences in pro-apoptotic UPR outputs.
On the chemoresistance phenotype, apoptosis is assessed at a single cisplatin dose (Fig. 4B; Suppl. Fig. 3B,C), showing ~8–10% fewer apoptotic cells with p38β overexpression, an effect reversed by BiP knockdown or the T648A mutant. While significant, this protection is modest. To strengthen the claim, a dose–response viability assay (to calculate IC50) or a clonogenic survival assay would be more informative.

4. Tumor-initiating cell assays and in vivo experiments – statistical rigor and clarity: The in vivo limiting-dilution assays (Figure 2K–O and Supplementary Fig. 1) are a crucial part of the paper, demonstrating that p38β enhances tumor-initiating capacity in immunodeficient mice. To ensure these findings are robust, the experimental design and statistical analysis need to be clearly and rigorously presented.

In the primary transplantation (Fig. 2K–M), MHCC97L cells with p38β-WT, Mut, or control were injected at varying cell numbers (e.g., 10^6, 10^5, 10^4, etc.) to assess tumor formation rates. Please clarify the number of mice injected per group at each cell dose. The text suggests that some groups had as few as two mice, while others had up to eight (e.g., at 10,000 cells, 8/8 tumours in p38β-WT vs. 2/8 in controls). Such unequal n can bias extreme limiting dilution analysis (ELDA) and complicate interpretation. Report the TIC frequencies with 95% confidence intervals and likelihood-ratio test P-values from ELDA. If certain groups were underpowered, note this as a limitation and emphasise the secondary transplantation (Fig. 2N–O), where the effects appear more consistent and robust.

One suggestion/optional: if not already done, the authors could mention whether they attempted any in vivo "rescue" reintroducing p38β-WT or Mut into KO cells before injection, to see if tumour formation is restored in a kinase-dependent manner. If such experiments were not performed, a brief acknowledgment would suffice, noting why (e.g., KO cells difficult to expand) and suggesting it as a potential follow-up.

For the ex vivo limiting-dilution spheroid assay from PDX tumors (Fig 6D), please make explicit in the legend/text that ELDA was used (the plot already shows ELDA-style "log fraction non-responding" lines and frequency estimates). Reporting the ELDA-derived TIC frequencies with 95% confidence intervals and the likelihood-ratio test p-values would let readers gauge effect size and uncertainty directly. Also, briefly clarify dissociation and plating at equal starting cell numbers, the number of wells per dilution, and how spheres were scored (and whether scoring was blinded).

The IHC quantifications of p-IRE1α, PCNA, and TUNEL (Fig. 6F-H) are valuable for showing mechanism. The legend notes n=8 mice per group and one-way ANOVA; please clarify whether statistics were performed on per-animal means (i.e., multiple fields averaged per tumor) and whether field selection/scoring were blinded. If feasible, provide exact p-values (or maintain asterisks) and confirm that the same analysis pipeline was applied across markers.

For the PDO experiments, the legend indicates two independent HCC organoid lines with a raw dose–response matrix (J). Please state explicitly that two PDOs were tested and report the synergy metric and score (e.g., Bliss or Loewe; values are shown in Supplementary synergy surfaces). Also indicate the number of biological replicates for the CellTiter-Glo viability assay and whether effects were consistent across both PDOs.

5. Interpretation of correlative clinical data (expression and patient outcomes): The manuscript integrates some clinical data (from patient cohorts and cell line databases) to support the experimental findings. These analyses are generally supportive

but should be interpreted and described with care to avoid overstatement and to provide clarity on how they were done.
In Figure 1D, the authors show that HCC patients with high p38β (MAPK11) mRNA expression have significantly worse overall survival than those with low expression. This is an important correlation suggesting p38β is a negative prognostic factor. However, it's essential to specify how the "high" and "low" groups were defined. The caption or methods should state whether this was a median split, upper quartile vs lower quartiles, or an "optimal" cutoff determined by something like maximally selected rank statistics. The note that the high vs low groups were of unequal size (e.g., 41 high vs 60 low for p-BiP in Fig. 4E) suggests the authors may have chosen a cutoff that wasn't the median (possibly upper third). It would add rigor to report the hazard ratio (HR) and 95% confidence interval for high vs low p38β from a Cox proportional hazards model, along with the p-value.

Figure 4E presents a survival analysis based on BiP Thr648 phosphorylation, a very interesting analysis that links the mechanism directly to clinical outcome. To strengthen this, please clarify how p-T648 was measured (e.g., immunohistochemistry on a tissue microarray) and how "high" expression was defined (H-score threshold, percentage positivity, etc.). The text notes 41 vs 60 patients, suggesting a non-median cut-off (perhaps top 40%); please confirm. Reporting hazard ratios with 95% confidence intervals would also add rigour. In Figure 1J, the correlation between MAPK11 expression and cisplatin IC50 is statistically significant but modest ($r \approx -0.33$) and should be described as a moderate association, not a strong one. Likewise, in Figure 2C, the higher MAPK11 expression in AFP-high tumours ($p = 0.042$) represents only a mild association, given the small effect size and the limitations of AFP as a stemness marker. Together, these findings are consistent with the overall model but should be presented with cautious wording to avoid overstating their strength.

Minor Comments:
• Figure labelling & self-containment: Add/confirm axis titles, units, n's, and definitions of asterisks throughout. Fig. 2A: include a colour scale (log2 fold-change or z-score) and label a few sentinel genes (e.g., AFP, EPCAM, ALB). Fig. 2G: mark the kinase domain and TGY activation loop (T180/Y182). Fig. 4C–D: state clearly these panels derive from phosphoproteomics and clarify the plotted intensity units.
• Survival plots (Figs 1D, 4E): In legends, include group sizes (n), data source (e.g., TCGA, IHC cohort), statistical test (log-rank), and—ideally—hazard ratio with 95% CI. Briefly state how "high/low" was defined (median, quantile, or optimised cut-off).
• Terminology consistency: Use "tumour-initiating capacity (TIC)" for function and "TICs" for the cells; prefer "self-renewal capacity" over broad "stemness" when referring to assays. Fix minor typos (e.g., "Tumour-initiating"). Define all abbreviations at first use (TIC, UPR, PDX, PDO, etc.).
• Tone/claims: Avoid superlatives unless effect sizes are large. Rephrase "strong" or "marked" effects as "moderate" or "significant" where appropriate (e.g., apoptosis decrease ~8–10%; p-PERK changes modest vs p-IRE1α).
• Proteomics display (Fig. 3A): Replace or complement the hit list with a small volcano or enrichment bar plot highlighting BiP and known p38β partners (e.g., MAP2K3, MKNK1, DUSP9), and indicate replicate statistics/enrichment over control IP. In addition, all raw proteomics files (.raw) should be deposited in PRIDE (ProteomeXchange) and made publicly available with the corresponding accession number.
• Graphical model (optional): A compact schematic of the p38β→BiP(T648)→IRE1α/PERK→UPR→TIC/chemoresistance axis would help readers and could double as a graphical abstract.

Reviewer #3

(Remarks to the Author)
In this manuscript, Huang et al. reported the identification of the stress kinase p38b as a putative regulator o tumour-initiating cells and drug resistance in HCC. They proposed that p38b promotes BiP phosphorylation, increasing its association with the unfolded protein response sensors PERK and IRE1-a. Such modification suppresses unfolded protein response activation and reduces unfolded protein accumulation. Potential therapeutic relevance of these findings is proposed using PDX and tumoroid models. The manuscript is well structured and written, fluent in the way to document outcomes and strategies undertaken to address specific points. There are several concerns that should be addressed.

Specifically:
1) Introduction: I strongly recommend to provide a better-deeper introduction about ER stress and UPR, rather than a simplistic overview.
2) Western blot results should be supported by quantifications and statistical analyses performed using independent experiments (and graphs should be shown in Supplementary Figures).
3) The authors should corroborate their study by analysing p38b levels (and relevant signals invoveld in the circuit they identified) using available proteomic data from a HCC patient cohort.
4) In the first section of results, using HCC spheroid cultures under repeated cisplatin and doxorubicin treatment, the authors must clarify which cells were used, how HCC spheroid cultures were established, and provide representative pictures in the Supplementary Figures, specifying which doses of cisplatin and doxorubicin were used. Moreover, they should show comparative drug dose responses of parental versus TIC-like cells, and western blots analysis (with quantifications) to confirm p38b enrichment in TIC-like versus parental cells.
5) line 102-104: The authors stated that "p38β protein levels were markedly increased in multiple HCC cell lines relative to the normal liver cell line MIHA". However, the results reported in Fig. S1A do not support this statement, as p38b is overexpressed only in 2 cell lines, whereas slightly upregulated in 3/5 lines. Moreover, in relation to this and the pertinence

of the cellular model used for studies, the authors performed most experiments using MHCC97L cells, with barely detectable levels of p38b. Findings should be supported using other cell lines, including intermediate (HepG2 and Huh7) and very high (PLC8024 and Hep3B) p38b levels. I also recommend to indicate in Figure panels which cell lines were used for different experiments, notably as there is no homogeneity on the cellular system used through studies.
6) Figure 1H-K: A large set of studies to assess the functional requirement of p38b has been essentially performed in PLC8024 HCC cells, in which p38b is overexpressed, thus possibly representing a specific signalling context. Moreover, cells have not been treated with chemotherapeutic, therefore the chosen model exemplify the requirement of p38b in HCC and not in relation to acquired chemo-resistance following treatments. Studies should be complemented with cellular settings modelling resistance acquisition following drug-treatment.
7) In Figures reporting patient data (e.g. Fig 1B-G, 2C-E, ...), I recommend to indicate the cohort used for analysis in panels (or in Figure legends).
8) The graph in Figure 4B reports a reduced CDDP sensitivity of about 5%, thus documenting a marginal rather than a "markedly reduced» sensitivity. Additional analyses are needed to strengthen the conclusion. As above, findings should be supported using other HCC cellular models.
9) line 289: The authors claimed that they used PDX and PDO from advanced HCC specimens exhibiting high p38b expression. Western blot analyses should document this statement.
10) line 292-294: The authors reported that “As expected, CDDP monotherapy moderately inhibited tumor growth, whereas the combination treatment led to a significantly greater reduction in both tumor volume and weight compared to CDDP alone”. However, results reported in Fig. 6A-C show no significant difference between CDDP alone versus CDDP+HA15. Considering the different capability of cells shown in ex vivo limiting dilution spheroid formation assay (Figure 6D), it is possible that the addition of HA15 to CDDP essentially depletes self-renewal capacity of HCC cells, without impacting tumour growth. Additional experiments should strengthen this hypothesis, for example: a) assessing tumour evolution over time after stopping the treatment in mice; b) using cells from treated tumours not only for ex vivo limiting spheroid formation assays, but as well for new in vivo xenograft studies. Moreover, cancer stem cell like markers from these tumours should be reported.
11) Based on tumoroid studies, the authors claimed that there is “a strong synergistic interaction” between CDDP and HA15, although synergy is essentially observed at high doses. Findings should be corroborated using additional tumoroids, and importantly using as well liver organoids to assess toxicity effects.
12) In consideration to the current HCC treatment essentially based on ICI and RTK inhibitors, the authors should discuss the relevance of their findings in the context of treatments other than with CDDP, and in relation to the still putative relevance of CDDP for liver cancer therapy (citing recent and pertinent references, e.g. PMID: 37446035).
13) Some material and method parts must be implemented. For example, concerning "in vitro limiting dilution tumorsphere assay": which medium was used to perform the assays? After how many days samples were processed and quantified? Moreover, drug doses used in all experiments must be reported in figure legends or panels, notably as apparently different according to experiments performed.

Reviewer #4

(Remarks to the Author)
The fundamental relevant observation of this work is that Bip is phosphorylated at threonine 648 (Thr648) (Figures 4C,D), near the C-terminus of the protein, a modification not previously linked to p38β signaling. This phosphorylation site is novel and given the large number of known BiP interactors this point itself is important and at the same time – surprising. The current work links it by several experiments to the oncogenic phenotypes, showing that expression of TA648 mutated BiP increased significantly the tumor-initiating frequency and tumor burden (Fig. panels). Collectively, the authors suggest based on their findings that BiP Thr648 phosphorylation is required for the observed p38β-driven stemness and drug resistance. The manuscript claims that the authors have previously demonstrated that the novel phosphorylation is due to the p38β kinase, and that the kinase is stress-induced, though this important and novel point is not elaborated and data supporting these claims are thin in the current data set. These data include exclusive reliance on western blots; more direct gels that report on phosphorylation (e.g. Phostag gels) are absent. There is also only cursory characterization of the antibodies used; This is pertinent as multiple Isoforms of various HSP70 isoforms exist and the current work does not show the BiP is specifically the recipient of the phosphorylation. A second and third major issues are that most, if not all the evidence is derived from a) the use of cell lines (e.g. MHCC97L cells) and b) is based on over- and under-expression in them. Even the in vivo models involve animals in which the in vitro-altered cells affect tumor cell properties after implantation. There is no verification of the interventions used, for example via endogenous specific gene disruption. Thus, the experimental system is inherently complex the biochemical characterization is vague and is not backed up by in vivo experiments that cleanly show the novel phosphorylation of BiP (rather than a related HSP70) by p38β.
The bulk of other data is concerned with the effects of the phosphorylation event on down stream UPR sensors and on consequent signaling and tumor biology. These data are extensive but, in my opinion, the central conclusion that ”BiP phosphorylation status critically shapes UPR signaling outcomes” is not sufficiently supported by the data for the reasons detailed above.

Reviewer #5

(Remarks to the Author)
General assessment
This manuscript identifies p38β (MAPK11) as a driver of chemoresistance and tumor-initiating capacity in hepatocellular

carcinoma (HCC) via phosphorylation of BiP at Thr648, suppression of the unfolded protein response (UPR), and maintenance of stemness. The authors provide a comprehensive dataset integrating screening, phosphoproteomics, functional assays, and validation in PDX/PDO models. The translational relevance is highlighted by pharmacological targeting with HA15.
The work is innovative and of potential impact. However, several critical gaps remain that need to be addressed to consolidate the mechanistic claims and ensure reproducibility.

Major comments
1. Isoform specificity of p38β
Current experiments focus solely on p38β. Although p38β ranked highest in the DEMETER2 RNAi screen, other isoforms (p38α/γ/δ) were not examined. No data compare whether the observed effects are unique to p38β. This leaves open the possibility of redundancy. Additional knockdown/overexpression or pharmacological inhibition experiments are needed to establish isoform specificity.
2. Direct substrate validation of BiP Thr648
The authors detected BiP Thr648 phosphorylation in phosphoproteomics and confirmed the site by MS/MS. Functional rescue with T648A and T648D mutants further supports its role. However, the causal link between p38β activity and BiP Thr648 phosphorylation remains indirect. Stronger evidence could be provided by demonstrating loss of Thr648 phosphorylation following genetic or pharmacological inhibition of p38β, or by generating endogenous knock-in BiP mutants (T648A/D) to validate physiological relevance.
3. Exclusion of other candidate genes
The initial screen identified four additional candidates (TYK2, FGFR4, PRKD2, SRPK3). These are listed but not functionally tested. Without even minimal validation, it remains uncertain whether their contribution to chemoresistance and TIC maintenance has been excluded. Comparative knockdown assays (±cisplatin, sphere formation, UPR readouts) would help rule out alternative explanations and reinforce the uniqueness of p38β.
4. Comprehensive UPR readouts
Mechanistic experiments mainly assess p-PERK, p-IRE1α, and unfolded protein accumulation by TPE-MI. ATF6 was reported as undetectable, but other canonical UPR markers (ATF4, CHOP, phospho-eIF2α, spliced XBP1) were not measured. Including these readouts would strengthen the claim that p38β–BiP signaling broadly suppresses the UPR.
5. Clinical data analysis
Patient cohort data are analyzed by correlation tables and Kaplan–Meier survival curves. However, no multivariate Cox regression was presented to confirm whether p38β (or p-BiP T648) is an independent prognostic factor after adjustment for TNM stage, vascular invasion, AFP, etc. Such analysis is required to establish prognostic independence.

Minor comments
1. Provide more detail on phosphoproteomic data analysis (localization probability thresholds, FDR, multiple testing correction) and make raw spectra available as supplementary data.
2. Include baseline clinical and demographic characteristics of the PDX donor patients, including age, sex, disease stage, treatment history, and race/ethnicity, so that readers can better appreciate the representativeness of the models used.
3. Ensure transparent reporting of sample sizes, replicates, effect sizes, and exact p-values in figure legends, specifying whether randomization and blinding were applied in PDX studies.
4. Expand the discussion to differentiate p38β-specific biology from the better-studied p38α isoform, citing prior literature.

Summary recommendation
The manuscript is promising but requires major revision. Specifically, demonstration of p38β isoform specificity, stronger evidence linking BiP Thr648 phosphorylation to p38β activity, minimal functional testing of other candidate genes, expansion of UPR readouts, and multivariate survival analysis are necessary. Addressing these points will significantly strengthen the mechanistic depth, originality, and translational relevance of the study.

Version 1:

Reviewer comments:

Reviewer #1

(Remarks to the Author)
The revised manuscript satisfactorily addresses all of my previous concerns, primarily through the inclusion of additional data that strengthen the authors' conclusions. I therefore recommend publication.

Reviewer #2

(Remarks to the Author)
I thank the authors for their careful and substantial revision of the manuscript. The revised version is significantly improved, and I find that the authors have addressed the major scientific concerns raised in the previous round of review in a satisfactory manner. In particular, the additional mechanistic experiments, the strengthened validation of the p38β–BiP axis, and the expanded in vitro and in vivo analyses have considerably reinforced the study and improved the overall rigor of the work.

In my view, the manuscript is now acceptable in principle. I have only one remaining point that I would ask the authors to address before final acceptance.

Because the proteomics/interactome dataset is used to support a central mechanistic conclusion, namely the prioritization of BiP as a key p38β interactor, I think this part of the manuscript should be presented in a more statistically rigorous manner. Specifically, I would request that the authors provide a proper volcano plot for the TAP-MS/interactome experiment, based on replicate-level quantitative protein intensity values and accompanied by the appropriate statistical analysis. This would allow the reader to directly evaluate both enrichment and statistical significance relative to the control condition and would substantially strengthen the presentation of the proteomics evidence.
Aside from this point, I consider the revision satisfactory and believe that the manuscript is suitable for publication after this minor final improvement.

Reviewer #3

(Remarks to the Author)
The revised version of this manuscript addresses most of my main concerns. However, some points still need to be addressed. Specifically:
- I requested that the western blot results be supported by quantifications and statistical analyses performed using independent experiments, with graphs shown in the Supplementary Figures. The authors replied: “We have now included quantitative analyses for all Western blot data, with densitometric intensities normalized to their respective loading controls”. Unless I am mistaken, I am unable to find these quantifications and statistical analyses in the Supplementary Figures of the revised manuscript.
- The authors now clarify the use of their previously published cDNA microarray profiling of chemoresistant hepatic tumourspheres generated by repeated cisplatin/doxorubicin selection. They specified that tumour-initiating cells were enriched from the HCC PLC/PRF/5 cell line by serial passages in serum-free spheroid culture supplemented with doxorubicin (2 mg/mL) and cisplatin (1 mg/mL), selecting for a self-renewing, chemoresistant population. In my opinion, this is a major concern for the study, as these doses correspond to about 3 mM, whereas these drugs are generally used at concentrations between 0.3 and 10 µM (thus 300 to 10,000 times higher than those generally used). For example, in a recent paper by Wang et al. (Cell Death Discovery 2024), the authors reported a dose response using doxorubicin within a range of 100 ng/mL up to µM. The use of cells and data obtained at these extremely high doses, which are far from being physiologically and clinically relevant, might affect the pertinence of the outcomes and bias their relevance. The authors must clearly state this experimental context at the beginning of the results section where the data are mentioned, with a paragraph such as: “Briefly, tumour-initiating cells were enriched from the HCC cell line PLC/PRF/5 by serial passages in serum-free spheroid culture supplemented with doxorubicin (2 mg/mL) and cisplatin (1 mg/mL), selecting for a self-renewing, chemoresistant population. The chemoresistant tumourspheres and their differentiated counterparts were subjected to cDNA microarray profiling. We acknowledge that this transcriptomic profiling of chemoresistant hepatic tumourspheres was obtained using drug doses 300–10,000 times higher than those generally employed in experimental and clinical settings”.

Reviewer #5

(Remarks to the Author)
The revised manuscript has been substantially improved. The authors have addressed many of the previous concerns, particularly regarding kinase activity validation, mechanistic linkage between p38β and BiP, and expansion of UPR-related readouts. The additional biochemical and in vivo data have strengthened the central conclusions, and overall the study is now more rigorous and convincing.

However, one important issue remains insufficiently resolved.

Major comment

Prioritization of p38β among the six candidate kinases remains insufficiently supported

The revised manuscript now provides a clearer description of the screening pipeline and explains how six overlapping kinase candidates were identified through integrated transcriptomic and dependency analyses. The authors further justify the selection of p38β based on its expression fold-change and the novelty of its link to BiP–UPR signaling.

While this rationale for prioritization is reasonable, it does not experimentally address the key concern: whether p38β is uniquely responsible for the observed phenotypes, or whether other shortlisted kinases (e.g., TYK2, FGFR4, PRKD2, SRPK3) may also contribute to chemoresistance and tumor-initiating capacity in HCC.

At present, no functional validation of these additional candidates has been provided. The response therefore represents a justification for focus, rather than a resolution of the concern. Given that several of these kinases are already implicated in cancer progression or drug resistance in other contexts, it remains difficult to exclude alternative or parallel drivers within the same system.

To strengthen the manuscript, the authors should consider:

Performing limited functional validation of one or two additional top candidates (e.g., knockdown ± cisplatin, sphere formation assays, or UPR-related readouts) to demonstrate that they do not recapitulate the p38β-dependent phenotypes; or

Explicitly tempering the claims throughout the manuscript to indicate that p38β is a prioritized candidate from the shortlist, rather than the uniquely dominant regulator identified by the screen.

Addressing this point would significantly enhance the rigor of candidate selection and strengthen the overall conclusions.

Reviewer #6

(Remarks to the Author)
The authors have adequately addressed the concerns raised in the previous review of rewiever and have provided well-argued responses supported by additional experimental data. In particular, the inclusion of Phos-tag SDS-PAGE, in vitro kinase assays, phospho-threonine detection on immunoprecipitated BiP, and additional controls addressing HSP70 family specificity substantially strengthens the evidence that BiP is phosphorylated by p38β at Thr648.
The authors have also responded appropriately to the concerns regarding the reliance on cell-line-based systems by adding in vivo data using AAV8-mediated p38β expression in HCC PDX models. These experiments provide further support for the specificity and functional relevance of the p38β–BiP phosphorylation axis in tumor growth and stemness-related phenotypes.
In addition, the authors appear to have carefully addressed the comments raised by the other reviewers, and the revised manuscript is overall clearer, better supported, and more balanced. The additional data and clarifications provided in the rebuttal adequately strengthen the main conclusions of the study.
Overall, I consider that the authors have responded appropriately and convincingly to the reviewers' concerns. In my opinion, the manuscript can now be accepted in its present form for publication in Nature Communications, without the need for further major revisions.

Reviewer #7

(Remarks to the Author)
I co-reviewed this manuscript with one of the reviewers who provided the listed reports. This is part of the Nature Communications initiative to facilitate training in peer review and to provide appropriate recognition for Early Career Researchers who co-review manuscripts.

Version 2:

Reviewer comments:

Reviewer #2

(Remarks to the Author)
The authors have satisfactorily addressed the substantive points raised in Reviewer 2. Although the final request regarding the statistical presentation of the proteomics data was not implemented exactly in the form suggested, the authors provided a reasonable explanation for this limitation and improved the presentation accordingly. Overall, I consider the revision satisfactory.

Reviewer #3

(Remarks to the Author)
The authors have addressed the points raised adequately and provided quantification and statistical analysis for some, although not all, western blot analyses.

Open Access This Peer Review File is licensed under a Creative Commons Attribution 4.0 International License, which permits use, sharing, adaptation, distribution and reproduction in any medium or format, as long as you give appropriate credit to the original author(s) and the source, provide a link to the Creative Commons license, and indicate if changes were made.
In cases where reviewers are anonymous, credit should be given to 'Anonymous Referee' and the source.
The images or other third party material in this Peer Review File are included in the article's Creative Commons license, unless indicated otherwise in a credit line to the material. If material is not included in the article's Creative Commons license and your intended use is not permitted by statutory regulation or exceeds the permitted use, you will need to obtain permission directly from the copyright holder.
To view a copy of this license, visit https://creativecommons.org/licenses/by/4.0/

Reviewer #1

This paper reports that expression of the stress kinase p38β is increased in hepatocellular carcinoma (HCC) spheroids cultured with cisplatin and doxorubicin. Moreover, high p38β expression correlates with poor prognosis and aggressive clinical features in HCC patients. The downregulation of p38β in a HCC cell line that expresses high levels of this kinase reduces its foci forming ability and increases cisplatin-induced cell death. Conversely, overexpression of p38β WT, but not of a non-phosphorylatable mutant, in a HCC cell line that expresses low levels of the kinase improves foci formation and the ability to form tumors in mice. The ER chaperone BiP is found to interact with p38β, and shRNA-mediated BiP downregulation reverts the reduced death observed in cisplatin-treated cells upon p38β overexpression. Phosphoproteomic analyses detect an enhanced phosphorylation of BiP on Thr648 in p38β overexpressing cells. The overexpression of p38β facilitates the co-immunoprecipitation of BiP with the UPR sensors/activators IRE-1a and PERK. Overexpression of BiP mutants shows that mutation of Thr648 to Ala impairs the interaction with IRE-1a. In tunicamycin-treated cells, overexpression of p38β or a BiP mutant with Thr648 mutated to Glu, but not to Ala, reduces unfolded protein accumulation, whereas downregulation of p38β or BiP increases unfolded protein accumulation. Finally, pharmacological inhibition of BiP using HA15 potentiates the cisplatin activity in suppressing the growth of a patient-derived xenograft and two organoid models, which correlates with increased detection of phospho-IRE-1a signal in tumors.

The manuscript contains a substantial amount of data, the narrative is appealing and some observations are consistent with the conclusions but several key claims are not really demonstrated. The proposal that p38β contributes to stemness and chemoresistance to cisplatin in HCC is a new and interesting finding, which could potentially have therapeutic implications. Evidence is presented that this function of p38β is somewhat mediated by BiP. The results support the idea that BiP phosphorylation on Thr648 regulates the UPR, probably by facilitating its interaction with some UPR sensors/activators, and contributes to cancer cell stemness or survival, but the link with p38β is very weak. The fact that a BiP protein with Thr648 mutated to Ala interferes with some effects of p38β overexpression does not necessarily mean that p38β regulates Thr648 phosphorylation.

It is also difficult to know what was actually done in several experiments due to the scarcity of information provided. This includes the initial analysis that led to the identification of p38β as a potentially regulator of HCC, the proteomic and phospho-proteomic experiments to identify p38β-BiP interaction and BiP phosphorylation, and the generation of the PDX and PDO models used. Important controls are missing in some experiments. Overall, the results are not as conclusive as the authors seem to believe, and without further experimental support it is unclear whether the authors might be correct in their conclusions.

Below are specific comments and points that should be addressed to provide support for the authors' conclusions in a more convincing manner.

We thank the reviewer for the constructive comments. Please refer to our response to each specific comment below.

1. The experimental pipeline that led to the identification of p38β is not properly described. Fig. 1A indicates the use of cDNA microarrays, but there is no description of the HCC spheroids used and how they were treated, the type and processing of the microarrays, the samples that were compared or how the analysis was performed to select 31 candidates. Results of the microarray should be presented as supplementary material, and raw data deposited in a database. The analysis of the DEMETER2 dataset that led to the identification of 37 candidates

should be explained. The criteria to rank p38β as #1 should be also described. As it stands, it is not clear why p38β was selected for further study.

Reply: We thank the reviewer for this comment and agree that more detailed description should be provided. In this study, we used our previously published cDNA microarray profiling of chemoresistant hepatic tumorsphere (TIC-enriched) model generated by repeated cisplatin/doxorubicin selection (GSE53005) (Lee et al., Hepatology, 2014; PMID: 24523067) and identified differentially expressed kinase genes between chemoresistant hepatic tumorspheres and their differentiated counterpart. Briefly, tumour-initiating cells were enriched from HCC cell line PLC/PRF/5 cells by serial passages in serum-free spheroid culture supplemented with doxorubicin (2 mg/mL) and cisplatin (1 mg/mL), selecting for a self-renewing, chemoresistant population. The chemoresistant tumorsphere and their differentiated counterpart were subjected to cDNA microarray profiling. From this published dataset, we selected only the kinase genes and identified 37 differentially expressed kinase genes with $\log_2$ fold-change>0.5. To prioritize kinases that are important for HCC cell survival, genetic dependency scores of HCC cell lines obtained from the DepMap DEMETER2 RNAi dataset (https://depmap.org/R2-D2/) were compared. Kinase genes were ranked by their average dependency across HCC lines (0 indicates no dependency, and more negative values indicate stronger dependency), and we applied a heuristic cutoff (gene effect ≤ -0.3) to define a candidate set, yielding 38 kinases. These two gene lists were integrated and revealed 6 overlapping kinases required for both chemoresistance and cell survival, with p38β (MAPK11) emerging as a top candidate with the largest expression fold-change between HCC tumor and non-tumor samples from TCGA -LIHC database (Fig. 1A).

Accordingly, more detailed description of the analysis has been included in Results (lines 111-124, page 6).

In this line, the references to "kinome-wide screen" and "kinase dependency" in Abstract and Discussion are misleading as no functional assays were apparently done for the selection of p38β.

Reply: We have revised "kinome-wide screen/kinase dependency screen" in Abstract and Discussion to avoid implying a high-throughput functional screen (lines 41-43, page 3; lines 439-440, page 18).

2. The experiments that investigate the effect of p38β downregulation are important and it is critical to show in all these experiments a WB of p38a to confirm the specificity of the reagents used. This includes Fig. 1H and Suppl. 4A.

Reply: Thank you for the suggestion. We have performed additional Western blots to evaluate p38α (MAPK14) levels in the experiments with p38β depletion (Fig. 1L; Supplementary Fig. 8A). Results show that p38β knockout did not alter p38α expression, confirming the specificity of p38β sgRNA.

Descriptions and antibody information have been added to Results and Methods – Reagent or resource (lines 149-151, page 7; lines 563, page 23).

3. Fig. 1K. The raw data in the image (left) do not correlate with values in the histogram (right). In the raw data LacZ is higher than Parental and about the same as sg p38β-2. These results indicate that only sg p38β-1 but not sg p38β-2 has an effect.

Reply: Thank you for pointing out the mismatch between the representative FACS plots and the bar chart in LacZ group. We crosschecked the raw data and observed a mistake in data input leading to a miscalculation of the net annexin V+ cells in the LacZ group in one set of the

independent experiments. We apologize for the confusion. We have repeated three independent experiments and consistently confirms our original conclusion that both sg p38β-1 and sg p38β-2 significantly increased the proportion of Annexin V+ cells after CDDP treatment relative to the parental and LacZ controls.

Fig. 1N has been updated with the representative FACS plots and the corresponding bar chart with statistical analysis. Raw data of the independent experiments are included in the Source Data file.

4. The cell lines MHCC97L, which expresses low levels of p38β, and PLC8024, which expresses high levels of p38β, are used to investigate the effects of p38β overexpression and downregulation, respectively. Since only one cell line is used in each case, it would be important to show that the distinctive levels of p38β expression in MHCC97L and PLC8024 cells correlate with different stemness properties and cisplatin sensitivity in the two cell lines.

Reply: Thank you for the suggestion. We conducted a sphere-formation assay and measured the cisplatin IC_{50} using the cell viability assay to evaluate stemness properties and cisplatin sensitivity in MHCC97L (p38β-low) and PLC8024 (p38β-high) HCC cell lines. Compared with MHCC97L, PLC8024 exhibited stronger self-renewal capacity (Supplementary Fig. 1B) and lower cisplatin sensitivity (Supplementary Fig. 1C). These results imply that p38β expression is positively associated with stemness and inversely correlated with cisplatin sensitivity in HCC cell lines.

Corresponding updates have been made to Results (lines 140-144, page 7).

5. Fig. 2H. The phospho antibody used recognizes the two residues that are mutated in p38β, and the lack of signal in Mut is therefore expected. A WB with an antibody that recognizes a downstream substrate of p38β (i.e., MK2) would be more relevant to confirm the pathway activity status in cells that overexpress WT or mutant p38β.

Reply: Thank you for the suggestion. We examined the phosphorylation of MAPKAPK2 (MK2), which is the direct phosphorylation substrate of p38β, in MHCC97L cells overexpressing either p38β WT or Mut. The result show that p38β WT significantly increased MK2 phosphorylation, whereas p38β Mut shows similar level as in empty-vector (EV) control (Fig. 2F). This confirms that p38β WT is functionally active and capable of activating its downstream substrate MK2.

Corresponding update has been made to Results (lines 188-194, page 8).

6. Immunoprecipitation experiments should always include an IgG control to demonstrate the specificity of the interactions, but this is not used in any of the experiments presented. This control is especially critical in the samples that show the interaction of overexpressed p38β with other proteins, i.e., p38β-overexpressing cells in Fig 3B, 3C and 5A, as well as the patient samples of Fig. 3E.

Reply: We apologize for missing the IgG control. We repeated the immunoprecipitation (IP) experiments in Fig. 3B, 3C and 3E (now Fig. 3F), including an IgG control alongside the indicated antibodies (p38β or BiP). In all cases, no or minimal signal was detected in the IgG lanes, confirming the specificity of the observed interactions (Supplementary Fig. 5A-C). The experiment shown in Fig. 5A was performed with the same experimental groups, antibody, and IP conditions as in Fig. 3C, for which the IgG control has now been rigorously validated. Given that the identical experimental system already demonstrated high specificity in Supplementary Fig. 5B, we referenced those validated IgG control data.

7. There is no description of the phosphoproteomic experiment that led to the identification of increased BiP phosphorylation on Thr648 in p38β overexpressing cells. Methods describe in a very generic way how the samples were processed but the results are not presented anywhere. How many samples were analyzed, how much was the increase and compare with what? Results should be presented as supplementary material, and raw data deposited in a database.

Reply: We agree that a more detailed description of the phosphoproteomic experiment is important.

Regarding the co-immunoprecipitation procedures, MHCC97L cells co-expressing SFB-tagged BiP with p38β WT or kinase-dead p38β Mut, or EV control, were immunoprecipitated with streptavidin beads for SFB-tagged BiP pull-down. SFB-tagged BiP immunoprecipitates were subjected to SDS-PAGE and Coomassie Blue staining, using a single biological sample per condition (Supplementary Fig. 6B). The gel lanes were separately cut into slices and subjected to in-gel digestion. The tryptic peptides were subjected to phosphoproteomic profiling by LC-MS/MS. BiP Thr648 phosphorylation was quantified relative to EV and p38β-Mut and was significantly elevated in the p38β WT group (Fig. 4A, B).

Regarding the processing of mass spectrometry result, raw mass spectrometry data were processed using MaxQuant 1.6.0.13. Raw data was searched against Human UniProt FASTA database (April 2020) containing 31, 165 entries, and also targeted HSPA5/BiP targeted full length protein sequence, using settings as below: oxidized methionine (M), acetylation (Protein N-term) were selected as dynamic modifications, and Phospho STY, carbamidomethyl (C) as fixed modifications with minimum peptide length of 7 amino acids was enabled. Confident proteins were identified using a target-decoy approach with a reversed database, strict false-discovery rate 1% at peptide and peptide spectrum matches (PSMs) level; minimum ≥2 unique peptides, ≥2 peptide spectral matches (PSMs), localization probability score > 0.75, Delta score >80. All identified BiP phosphorylation sites and associated quantitative values are provided in Supplementary Table S5, and the raw MS/MS data have already been deposited in the PRIDE repository (accession PXD067039).

More detailed description of the experimental procedures has been added to Methods - Phosphoproteomic analysis by LC–MS/MS (lines 654-675, pages 29-30).

8. The link between p38β and BiP phosphorylation on Thr648 is not convincing. There is no evidence that p38β can phosphorylates BiP, which would require performing in vitro kinase assays. Likewise, no experiment is shown to support a link between p38β expression or activity and BiP-Thr648 phosphorylation in cells. For example, is this phosphorylation reduced in cells upon p38β downregulation or increased when cells overexpress p38β? Are the BiP-Thr648 phosphorylation levels different between MHCC97L and PLC8024 cells? A phospho-Thr648 antibody would be perfect for these experiments. Alternatively, BiP immunoprecipitations followed by WB with a generic phospho-Thr antibody could be tried, or phosphoproteomic analysis.

Reply: Thank you for the suggestions. We performed 1) an *in vitro* kinase assay using a recombinant activated His-tagged p38β protein (ThermoFisher, PV3679) and a recombinant BiP protein to demonstrate Thr phosphorylation of BiP; 2) BiP immunoprecipitation and phospho-Thr detection in p38β WT and Mut overexpressing cells; and 3) BiP immunoprecipitation and phospho-Thr detection in MHCC97L and PLC8024 cells. In the *in vitro* kinase assay, we showed that BiP is phosphorylated by the recombinant p38β protein at Thr residue (Fig. 4D). Stronger Thr-phosphorylation on BiP was observed in MHCC97L cells overexpressing p38β WT, but not in EV or p38β Mut (Fig. 4E). PLC8024 cells with higher p38β expression showed stronger Thr-phosphorylation on BiP when compared with MHC97L

cells with lower p38β expression (Supplementary Fig. 6C). Together with the identification of phosphorylation of Thr648 residue on BiP from the original phosphoproteomic MS result, these new results collectively demonstrate that Thr648 phosphorylation of BiP is dependent on the active p38β.

Corresponding updates have been made in Results and Methods - *In vitro* kinase assay and *In vitro* binding assay (lines 278-284, 286-289, pages 11-12; lines 625-643, pages 28-29).

9. If the link between BiP-Thr648 phosphorylation and p38β is confirmed, it would be important to explain how an ER-luminal protein like BiP can be phosphorylated by p38β, whose substrates are typically cytosolic or nuclear.

Reply: This is an important point to consider. Increasing evidence suggests that BiP is not exclusively confined to the ER lumen; under stress conditions and during malignant progression, a fraction of BiP has been detected outside the ER, including in cytoplasmic and cell-surface-associated pools (**1**, **2**). Conversely, although p38β is predominantly cytosolic, it can be activated at ER-associated sites, including the cytosolic face of the ER membrane and ER–mitochondria contact regions (**3**, **4**). Consistent with this spatial concordance, our confocal imaging revealed partial overlap between p38β and BiP within the cytoplasmic region, together with detectable proximity of p38β to ER structures (Fig. 3G). Collectively, these observations support the existence of a cytosolic or ER-associated pool of BiP that is spatially accessible to p38β for phosphorylation.

Updates have been made to Results and Discussion (lines 241-244, page 10; lines 471-481, page 19).

1. Lee, A.S. Stress-induced translocation of the endoplasmic reticulum chaperone GRP78/BiP and its impact on human disease and therapy. *Proc Natl Acad Sci U S A* **122**, e2412246122 (2025).

2. Sicari, D. *et al.* Reflux of Endoplasmic Reticulum proteins to the cytosol inactivates tumor suppressors. *EMBO Rep* **22**, e51412 (2021).

3. Li, L. *et al.* p38 MAP kinase-dependent phosphorylation of the Gp78 E3 ubiquitin ligase controls ER-mitochondria association and mitochondria motility. *Mol Biol Cell* **26**, 3828–3840 (2015).

4. Li, W. *et al.* Phosphorylation of LAMP2A by p38 MAPK couples ER stress to chaperone-mediated autophagy. *Nat Commun* **8**, 1763 (2017).

10. The in vivo experiment (Fig. 6) shows a moderate increase in the cisplatin effect upon combination with HA15. This effect is expected based on previous literature showing that BiP inhibition interferes with tumor growth, but the data provides support neither for the importance of BiP phosphorylation nor for the implication of p38β upstream of BiP

Reply: In this experiment, we aim to assess the therapeutic relevance of targeting BiP in combination with cisplatin for suppressing HCC stemness and tumorigenesis. The molecular mechanism between p38β and BiP phosphorylation have been established through biochemical, genetic, and mutant analyses in Fig. 3-5 using the *in vitro* system. To strengthen the mechanistic link *in vivo*, we established AAV8-mediated overexpression of p38β WT, Mut and EV control in human HCC PDX. Consistent with the cell line model, PDX with p38β overexpression showed stronger tumorigenicity and self-renewal properties (Fig. 2O-Q; Supplementary Fig. 4I-L). BiP phosphorylation was detectable in p38β WT group and was barely detectable in p38β

Mut and EV groups (Fig. 4F), demonstrating the dependence of BiP Thr-phosphorylation with p38β kinase activity.
Corresponding updates have been added to Results and Methods – Pharmacological treatment and intratumoral AAV8 delivery in HCC PDX (lines 210-217, page 9; lines 284-286, page 12; lines 695-704, page 31).

11. How was the PDX used in Fig. 6A-6C obtained and what are its characteristics? Same for the two PDOs used in Fig. 6I-6J.
Reply: The establishment of this HCC PDX model in Fig. 6A-6C is described in our previous publication (**1**). The PDX model was established from a resected primary HCC specimen obtained with informed patient consent and approval from the Institutional Review Board of the University of Hong Kong/Hospital Authority Hong Kong West Cluster. Clinicopathological characteristics of this PDX#1 donor patient are summarized in Supplementary Table S2. The two HCC PDOs used in this study are kind gifts from Dr. Meritxell Huch (Max Planck Institute) (**2**).
This information has been added to Methods - Cell lines, PDOs and PDX (lines 555-556, 559-562, pages 22-23).
1. Tong, M. *et al*. Efficacy of annexin A3 blockade in sensitizing hepatocellular carcinoma to sorafenib and regorafenib. *J Hepatol* **69**, 826-839 (2018).
2. Huch, M. *et al.* Long-term culture of genome-stable bipotent stem cells from adult human liver. *Cell* **160**, 299-312 (2015).

12. Related to Fig 5C, does p38β overexpression reduce Tunicamycin-induced cell death?
Reply: MHCC97L cells with p38β WT overexpression showed reduced net percentage of apoptotic cells compared with Mut and EV groups upon Tunicamycin treatment. (Supplementary Figure 8B).
Update has been made to Results (lines 346-347, page 14).

13. Two different p38β antibodies are mentioned in the Reagents section, and figure legends should indicate which antibody was used in each case.
Reply: The MAPK11/p38β rabbit polyclonal antibody (Cat# HPA045069) was used for immunohistochemistry of human clinical specimens and xenografts. Western blots and immunoprecipitation were performed using the MAPK11/p38β rabbit monoclonal antibody (Cat#2339).
The corresponding information has been supplemented in Methods - Reagent or resource (line 563, page 23).

Reviewer #2 (Remarks to the Author)
Summary:
This manuscript explores how the stress-activated kinase p38β (MAPK11) may promote tumor-initiating cell (TIC) properties and chemoresistance in hepatocellular carcinoma (HCC) by modulating the unfolded protein response (UPR) through the ER chaperone BiP. A kinome-wide shRNA screen in chemotherapy-resistant spheroids identified p38β as a candidate factor, and high expression was associated with poorer survival and adverse clinicopathological features. Functional studies suggest that loss of p38β reduces colony formation, self-renewal, and cisplatin resistance, while overexpression increases TIC frequency in vitro and in xenograft models. Mechanistically, p38β appears to phosphorylate BiP at threonine 648, which may enhance BiP binding to IRE1α and PERK, thereby attenuating UPR activation and limiting stress-induced apoptosis. Mutational analysis of BiP at Thr648 analyses the functional relevance of this modification, and suggests that pharmacological inhibition of BiP synergizes with cisplatin in patient-derived xenografts and organoids.
Overall, this work reveals a novel p38β–BiP signaling axis linking ER stress adaptation to cancer stemness and chemoresistance in HCC. The experiments address an important question and are generally well-designed, and the findings are of high interest for the HCC and cancer stem cell fields. However, there are several substantive issues regarding the strength of the evidence and the clarity of data presentation that should be addressed to solidify the conclusions. The suggested revisions are intended to improve the rigor and transparency of the study, thereby ensuring that the conclusions are fully supported by the data.

We thank the reviewer for the constructive comments. Please refer to our response to each specific comment.

Major Comments:
1. Requirement for p38β kinase activity and on-target specificity: The conclusion that p38β's kinase activity is essential for TIC maintenance and drug resistance should be reinforced with additional controls and broader validation. The current evidence relies on overexpression of WT vs. a T180A/Y182F "kinase-dead" mutant (p38β Mut) in MHCC97L, plus one knockout model. These experiments do suggest that kinase activity is necessary – for example, Figures 2I–J show that overexpressing p38β WT significantly increases colony formation and tumorsphere frequency compared to empty vector, whereas the kinase-dead mutant fails to do so (indeed performing similarly to the control). Likewise, p38β knockout dramatically impairs oncogenic growth (Fig. 1I) and increases apoptosis under cisplatin (Fig. 1K), indicating that p38β is required for these phenotypes. While these results are compelling, it is critical to confirm that the effects are truly due to p38β's catalytic function and not off-target consequences of overexpression or genetic manipulation.
First, please ensure that the kinase-dead mutant is expressed at a comparable level to WT p38β and is indeed catalytically inactive in cells. It is implied that expression was matched (Fig. 4G confirms similar protein levels for WT and mutant), but demonstrating loss of kinase activity would strengthen confidence. A straightforward way is to examine the phosphorylation of known p38 downstream targets (e.g., MK2 or HSP27) in the WT vs. Mut overexpressors. Providing a Western blot for phospho-MK2 or phospho-HSP27 in the empty vector, WT, and Mut backgrounds would be a strong validation that the mutant truly lacks kinase function in the cellular context.
Reply: Thank you for the comment and suggestion. We examined the phosphorylation of MAPKAPK2 (MK2) in MHCC97L cells overexpressing either p38β WT or Mut. The result

show that p38β WT significantly increased MK2 phosphorylation, whereas p38β Mut shows similar level as in empty-vector (EV) control (Fig. 2F). This confirms that p38β WT is functionally active and capable of activating its downstream substrate MK2, whereas Mut lacks kinase function.
Corresponding update has been made to Results (lines 188-194, page 8).

Second, the study would be strengthened by pharmacological inhibition experiments to ensure that blocking p38β activity phenocopies the genetic loss-of-function. Currently, no small-molecule p38 inhibitor data are presented. Treating p38β-overexpressing cells with a known p38α/β inhibitor (such as SB203580, or a more potent agent like BIRB796 or Ralimetinib) and showing a reversal of the TIC phenotypes would provide crucial on-target evidence. One approach is to combine inhibitor treatment with selective knockdown of p38α (MAPK14). If, in p38β-overexpressing cells, the inhibitor still reduces sphere formation or enhances cisplatin-induced cell death even after p38α is silenced, this would indicate the drug effect is likely acting through p38β. In any case, a pharmacological validation of p38β's role in maintaining the TIC phenotype would significantly bolster the conclusions.
Reply: Thank you for the suggestion. We established siRNA-mediated p38α silencing in both MHCC97L cells with p38β wild-type (WT) overexpression and EV control (Supplementary Fig. 2A). Pharmacological inhibition using the dual p38α/β inhibitor SB203580 suppressed tumorsphere formation in si-p38α + p38β WT cells, whereas such inhibition did not affect si-p38α + EV cells (Supplementary Fig. 2B), strengthening p38β's role in maintaining the TIC phenotype with the absence of p38α.
Corresponding updates have been made to Results (lines 173-179, page 8).

Additionally, extending the core functional assays to a second HCC cell line would improve generalizability. The current in vitro data are largely from MHCC97L (a line with relatively low endogenous p38β). It would be informative to test p38β loss-of-function in an HCC line that naturally has high p38β expression (to see if they depend on it), or conversely to overexpress WT vs. Mut in another low-p38β line (such as Huh7 or Hep3B, if they have lower MAPK11 levels) and assess colony formation, sphere formation, and drug response.
Reply: We have performed loss-of-function experiments by CRISPR-Cas9 mediated knockout of p38β in PLC8024 cells with high p38β expression (Supplementary Fig. 1A). Knockout of p38β resulted in reduced colony formation, increased cisplatin sensitivity, and suppressed tumorsphere formation (Fig. 1M, 1N, Supplementary Fig. 2C). In addition to the original results using PLC8024 and MHCC97L cells, we extended our core functional assays to an additional HCC cell line, Huh7, which exhibits relatively low endogenous p38β expression (Supplementary Fig. 1A). Consistent with the results observed in MHCC97L cells, overexpression of p38β WT, but not p38β Mut in Huh7 cells, significantly promoted colony formation, sphere formation, and resistance to cisplatin (Supplementary Fig. 3D-G). These results confirm that the oncogenic effects of p38β are reproducible across multiple HCC cell lines.
These new data using Huh7 cells are included in Supplementary Fig. 3D-G and Results (lines 194-199, pages 8-9).

2. Mechanistic link between p38β and BiP – biochemical evidence and specificity: The identification of BiP as a p38β-interacting protein and substrate is central to the paper's mechanism. While the data presented are intriguing, some aspects need more rigorous support to establish this interaction and modification as specific and functionally relevant.

In Figure 3A, a proteomics approach pulled down p38β and identified interactors, highlighting BiP as a top hit. However, it's not clear if this experiment was performed in replicates or how significance was determined. To bolster confidence in BiP as a bona fide interactor, the proteomic results should be quantified and presented with appropriate statistics (i.e. volcano plot) - using the intensity rather than spectral counts (#pep). BiP should clearly be labeled among the significantly enriched proteins, as well as the well-known MAPK11 partners such as MAP2K3, MKNK1 and DUSP9 (already displayed in the table, Fig 3A).

Reply: Tandem affinity purification coupled with mass spectrometry was performed in duplicate. The list of proteins identified as interacting with p38β was compared against proteins eluted from the empty vector control group. The complete set of unique binding proteins is now provided in Supplementary Table S4. Although only limited data, specifically the numbers of unique peptides and total peptides for each identified protein were available, these metrics were used to generate a scatter plot of mean total peptides versus mean unique peptides (Fig. 3A). Among the top 10 hits, OBSL1, CUL7, and CCDC8 (collectively known as the 3M complex) and HSPA5/BiP have not been previously reported as known binding partners or direct targets of p38MAPK or p38β. Although the 3M complex ranked higher than BiP based on mean total peptide counts, we prioritized BiP for further consideration as a potential therapeutic target due to its reported oncogenic function and the availability of a specific inhibitor. In contrast, the 3M complex has been reported to play a tumor-suppressive role in maintaining microtubule stability and genome integrity, making it less amenable to therapeutic targeting. We acknowledge that the 3M complex may also represent novel and potential downstream effectors of p38β. However, this requires further investigation.

The list of unique p38β-immunoprecipitated proteins identified by LC–MS/MS is provided in Supplementary Table S4. Fig. 3A is replaced with a scatter plot showing the known and unknown partners of p38MAPK or p38β. Corresponding updates have been made to Results and Methods - Tandem affinity purification followed by mass spectrometry (TAP-MS) (lines 223-225, pages 9-10; lines 644-653, page 29).

The co-immunoprecipitation experiments in Figure 3B and 3D validate that p38β and BiP form a complex in cells. One concern is whether this interaction is specific to the p38β isoform. Since the phospho-p38 antibody used recognizes both p38α and p38β when they are activated, it remains possible that p38α could also bind BiP during stress. To address this, please clarify whether p38α interacts with BiP under the same conditions. The authors could perform a parallel co-IP using p38α (MAPK14) as the target: immunoprecipitate p38α from HCC cell lysates (perhaps under stress conditions similar to those used for p38β IP) and probe for BiP. Alternatively, showing that the level of BiP co-immunoprecipitated with phospho-p38 in Figure 3D drops substantially when p38β is knocked down (leaving p38α as the main source of p-p38) would also indicate specificity. Establishing isoform specificity is important, because it underpins the idea that p38β has a unique role (as opposed to the well-studied p38α which might otherwise be assumed to perform similar functions).

Reply: Thank you for the suggestions and we agree that establishing isoform specificity is important. To further determine whether p38β-BiP interaction is isoform-specific, we performed parallel co-IP assay targeting p38α (MAPK14). Under identical experimental condition, p38α failed to pull down BiP in PLC8024 cells (Supplementary Fig. 5D). These findings establish an isoform-specific role for p38β with BiP interaction.

Corresponding update has been made to Results (lines 245-248, page 10).

Another mechanistic point is whether the p38β–BiP binding is direct or mediated by other proteins. The text implies a direct interaction, and the co-IP supports a physical association. If feasible, an in vitro binding assay (using purified p38β and BiP proteins) could be a definitive way to show direct binding. This is especially interesting because p38β is a cytosolic kinase, whereas BiP is primarily in the ER lumen; thus one might speculate that either a subset of BiP is present in the cytosol or that p38β transiently associates with the cytosolic side of the ER where it could access BiP's ATPase domain (which contains Thr648). A brief discussion of this will preempt reader curiosity on how a cytosolic kinase phosphorylates an ER luminal chaperone.

Reply: These are important points to consider. To determine whether p38β and BiP interaction is direct rather than mediated by additional cofactors, we performed a cell-free *in vitro* binding assay using recombinant His-tagged p38β and BiP proteins. Consistent with the cellular Co-IP results, BiP can be pulled down together with p38β *in vitro*, demonstrating a direct physical interaction independent of other cellular components (Fig. 3E).

Increasing evidence suggests that BiP is not exclusively confined to the ER lumen. Under stress conditions and during malignant progression, a fraction of BiP has been detected outside the ER, including in cytoplasmic and cell-surface-associated pools (**1**, **2**). Conversely, although p38β is predominantly cytosolic, it can be activated at ER-associated sites, including the cytosolic face of the ER membrane and ER–mitochondria contact regions (**3**, **4**). Consistent with this spatial concordance, our confocal imaging revealed partial overlap between p38β and BiP within the cytoplasmic region, together with detectable proximity of p38β to ER structures (Fig. 3G). Collectively, these observations support the existence of a cytosolic or ER-associated pool of BiP that is spatially accessible to p38β for phosphorylation.

Result of *in vitro* binding assay has been added to Fig. 3E. Immunofluorescence images have been added to Fig. 3G. Updates have been made to Results and Discussion (lines 234-238, page 10; lines 241-245, page 10; lines 471-481, page 19).

1. Lee, A.S. Stress-induced translocation of the endoplasmic reticulum chaperone GRP78/BiP and its impact on human disease and therapy. *Proc Natl Acad Sci U S A* **122**, e2412246122 (2025).

2. Sicari, D. *et al.* Reflux of Endoplasmic Reticulum proteins to the cytosol inactivates tumor suppressors. *EMBO Rep* **22**, e51412 (2021).

3. Li, L. *et al.* p38 MAP kinase-dependent phosphorylation of the Gp78 E3 ubiquitin ligase controls ER-mitochondria association and mitochondria motility. *Mol Biol Cell* **26**, 3828–3840 (2015).

4. Li, W. *et al.* Phosphorylation of LAMP2A by p38 MAPK couples ER stress to chaperone-mediated autophagy. *Nat Commun* **8**, 1763 (2017).

In Figures 4C and 4D, the authors present phosphoproteomic data showing that BiP is phosphorylated at Thr648 in p38β-overexpressing cells. Figure 4C shows that the normalized abundance of the BiP pT648 peptide is markedly higher in p38β-WT cells compared to empty vector, but the overall phosphosite intensity (10E4) is very low as well as a poor MS2 spectra (Figure 4D). These data point to BiP as a substrate of p38β, but more detail on this phosphosite identification should be provided. Was Thr648 one of the top upregulated phosphosites in the entire dataset, or at least among BiP peptides? Also, were any other BiP phosphorylation sites detected, and if so, did those change with p38β overexpression? Were downstream BiP

phosphorylation events also effected by Thr648? BiP has multiple known phosphosites, validation of that Thr648 was uniquely or most strongly regulated by p38β would emphasize its likely importance.

Reply: This phosphoproteomic analysis was performed using MHCC97L cells co-expressing SFB-tagged BiP with p38β WT, kinase-dead p38β Mut, or empty-vector (EV) controls, followed by immunoprecipition with streptavidin beads for SFB-tagged BiP pull-down. SFB-tagged BiP immunoprecipitates were subjected to SDS-PAGE and Coomassie Blue staining, using a single biological replicate per condition (Supplementary Fig. 6B). The gel lanes were separately cut into slices and subjected to LC-MS/MS. As this workflow analyzes a BiP-enriched immunoprecipitated fraction (rather than a whole-cell phosphoproteome), the overall signal intensity for individual phosphopeptides, including the Thr648-containing peptide, can be relatively low due to limited input. Nevertheless, within this MS results, phosphorylation at Thr648 showed the strongest enrichment in p38β WT compared with p38β Mut and EV. We detected two additional BiP threonine phosphosites (Thr159 and Thr171), but these did not show enrichment comparable to Thr648 upon p38β-WT expression, making Thr648 the most upregulated BiP phosphosite identified in our dataset. All identified BiP phosphorylation sites and their quantitative values are now provided in Supplementary Table S5, and the raw MS/MS data have been deposited in the PRIDE repository (accession PXD067039).

In this revised manuscript, we added multiple validation to confirm p38β-BiP, including an *in vitro* kinase assay with purified recombinant p38β and BiP proteins (Fig. 4D), p38β overexpression followed by BiP immunoprecipitation and anti-pan-phospho-threonine detection (Fig. 4E-F; BiP-pThr648–specific antibody is not available), and Phos-tag SDS-PAGE to visualize phosphorylation-dependent mobility shifts of BiP in p38β-overexpressing cells (Fig. 4C). To further establish the site specificity, we generated phospho-defective BiP-T648A and phosphomimetic BiP-T648D mutants and showed that phosphorylation on this T648 residue is crucial for p38β-dependent tumor stemness phenotypes and the underlying unfolded protein control and UPR regulation (Fig. 4J-Q and Fig. 5). All these results support Thr648 as the key residue regulated by p38β.

Corresponding updates have been made to Results (lines 274-286, pages 11-12). More detailed description of the experimental procedures has been added to Methods - Phosphoproteomic analysis by LC–MS/MS (lines 654-675, pages 29-30).

3. UPR suppression and chemoresistance phenotypes - quantification and additional readouts: A major claim of the paper is that p38β's phosphorylation of BiP dampens UPR signaling, thereby reducing ER stress levels and preventing cell death under chemotherapy. The experiments in Figure 5 and Supplementary Fig. 4 address this by measuring UPR sensor activation (IRE1α and PERK phosphorylation) and the accumulation of misfolded proteins (using the TPE-MI fluorescent dye) in various conditions. The trends observed are consistent with the model, but some of these effects should be quantified more rigorously and supported by additional readouts to fully convince the reader.

The most rigorous option would be to use targeted quantitative phosphoproteomics (PRM) to directly measure key UPR phosphopeptides (e.g., activation-loop sites in IRE1α and PERK). PRM would provide sensitive and precise quantification across conditions (EV/WT/Mut/KO, ± cisplatin or tunicamycin), yielding fold-changes with clear statistics. If PRM is not feasible, densitometric analysis of the existing Western blots across ≥3 independent experiments (normalising p-IRE1α and p-PERK to totals) should be performed to report mean fold-change ± SD/SEM and significance.

Reply: We thank the reviewer for this rigorous methodological recommendation. We agree that targeted quantitative phosphoproteomics represents the most rigorous option for precise and sensitive quantification of key UPR phosphopeptides. However, given current resource and time constraints, we performed densitometric quantification of the Western blot data examining UPR signaling across different experimental conditions (Fig. 5B, E, H; Supplementary Fig. 8A, E). Specifically, p-IRE1-α and p-PERK, p-eIF2α were normalized to their respective total protein levels. UPR effectors XBP-1s, ATF4 and CHOP were normalized to β-actin.

In addition, at least one downstream UPR effector readout (e.g., CHOP protein induction or XBP1 mRNA splicing) under stress would strengthen the mechanistic link. Reduced CHOP and less XBP1 splicing in p38β-overexpressing cells, and the opposite in p38β-KO or BiP-T648A contexts, would confirm that the biochemical suppression of UPR sensors translates into differences in pro-apoptotic UPR outputs.

Reply: Thank you for the suggestion. To strengthen the mechanistic link between p38β-mediated BiP phosphorylation and downstream pro-apoptotic UPR outputs, we have now examined both CHOP protein expression and XBP1 mRNA splicing under ER stress conditions. Specifically, we treated cells with cisplatin to induce ER stress and compared responses across EV, p38β WT, p38β Mut, and p38β WT overexpressing cells with BiP silencing and BiP mutants. The results show that p38β WT overexpression significantly attenuates CHOP induction and XBP1 splicing (Fig. 5B), whereas BiP depletion (Fig. 5E) or overexpression of BiP-T648A (Fig. 5H) in p38β WT overexpressing cells enhances both CHOP and XBP1 splicing, indicating that p38β-mediated phosphorylation of BiP dampens downstream pro-apoptotic UPR signaling.

Corresponding updates made to Results (lines 331-333, page 13; lines 354-358, page 14; lines 374-378, page 15).

On the chemoresistance phenotype, apoptosis is assessed at a single cisplatin dose (Fig. 4B; Suppl. Fig. 3B,C), showing ~8–10% fewer apoptotic cells with p38β overexpression, an effect reversed by BiP knockdown or the T648A mutant. While significant, this protection is modest. To strengthen the claim, a dose–response viability assay (to calculate IC50) or a clonogenic survival assay would be more informative.

Reply: Thank you for the suggestion. We have now performed clonogenic survival assays with the treatment of cisplatin across multiple experimental settings. We have conducted clonogenic assays in PLC8024 cells with p38 knockout (Fig. 1M), in MHCC97L and Huh7 cells with EV, p38β WT and Mut overexpression (Fig. 2G; Supplementary Fig. 3F). The clonogenic survival assay in p38β WT overexpressing cells with BiP mutants have been performed in the original submission (Fig. 4L; Supplementary Fig. 7A). Across all these contexts, the clonogenic survival assays revealed consistent and more pronounced differences than those observed in the apoptosis assays, further supporting that the p38β–BiP axis is important for the chemoresistance phenotype in HCC.

Corresponding updates have been made to Results (lines 151-152, page 7; lines 194-199, pages 8-9).

4. Tumor-initiating cell assays and in vivo experiments – statistical rigor and clarity: The in vivo limiting-dilution assays (Figure 2K–O and Supplementary Fig. 1) are a crucial part of the paper, demonstrating that p38β enhances tumor-initiating capacity in immunodeficient mice. To ensure these findings are robust, the experimental design and statistical analysis need to be clearly and rigorously presented.

In the primary transplantation (Fig. 2K–M), MHCC97L cells with p38β-WT, Mut, or control were injected at varying cell numbers (e.g., 10^6, 10^5, 10^4, etc.) to assess tumor formation rates. Please clarify the number of mice injected per group at each cell dose. The text suggests that some groups had as few as two mice, while others had up to eight (e.g., at 10,000 cells, 8/8 tumours in p38β-WT vs. 2/8 in controls). Such unequal n can bias extreme limiting dilution analysis (ELDA) and complicate interpretation. Report the TIC frequencies with 95% confidence intervals and likelihood-ratio test P-values from ELDA. If certain groups were underpowered, note this as a limitation and emphasise the secondary transplantation (Fig. 2N–O), where the effects appear more consistent and robust.

Reply: We apologize for the lack of clarity in the original submission that led to this misunderstanding. In the primary transplantation experiments (Fig. 2I-K), each experimental group contained eight mice (n = 8 per group). In the secondary transplantation experiments (Fig. 2L-N), six mice were used per experimental condition at each cell dose. The values shown in the table (Supplementary Fig. 4A), such as "8/8 tumors" or "2/6 tumors", indicate tumor incidence, where the first number represents the number of mice that developed tumors and the second number represents the total number of mice injected per group. Therefore, all groups were powered equally, and the observed differences in tumor formation rates reflect genuine, dilution-dependent differences in tumor-initiating cell (TIC) frequency rather than unequal sample sizes.

We have now included the TIC frequencies with 95% confidence intervals and likelihood-ratio test P-values from the extreme limiting dilution analysis (ELDA) in Supplementary Fig. 4A. These quantitative metrics confirm that p38β wild-type overexpression significantly increases TIC frequency compared to EV control and p38β Mut.

We have clarified these experimental details in the revised figure legends of Fig. 2I-N (lines 801-810, page 35).

One suggestion/optional: if not already done, the authors could mention whether they attempted any in vivo "rescue" reintroducing p38β-WT or Mut into KO cells before injection, to see if tumour formation is restored in a kinase-dependent manner. If such experiments were not performed, a brief acknowledgment would suffice, noting why (e.g., KO cells difficult to expand) and suggesting it as a potential follow-up.

Reply: We agree that an *in vivo* "rescue" experiment reintroducing p38β-WT or p38β-Mut into the p38β-KO cells prior to transplantation would provide further confirmation of the kinase-dependent effect on tumour formation. However, this experiment was not feasible in the present study because p38β-KO MHCC97L cells exhibit significantly impaired proliferation and viability, precluding their efficient expansion for stable re-expression and *in vivo* transplantation. We have now acknowledged this limitation in the Discussion as follows:

"Notably, although *in vivo* genetic rescue of p38β would further strengthen this causal link, the significantly reduced proliferative capacity of p38β-KO cells precluded their efficient expansion for stable re-expression and transplantation in the present study." (lines 462-465, page 19)

For the ex vivo limiting-dilution spheroid assay from PDX tumors (Fig 6D), please make explicit in the legend/text that ELDA was used (the plot already shows ELDA-style "log fraction non-responding" lines and frequency estimates). Reporting the ELDA-derived TIC frequencies with 95% confidence intervals and the likelihood-ratio test p-values would let readers gauge effect size and uncertainty directly. Also, briefly clarify dissociation and plating at equal

starting cell numbers, the number of wells per dilution, and how spheres were scored (and whether scoring was blinded).

Reply: We thank the reviewer for the comment. Regarding the use of Extreme Limiting Dilution Analysis (ELDA), we have revised the figure legend for Fig. 6D (now Fig. 6F) to explicitly state that ELDA was applied. The legend now reads: "Extreme Limiting Dilution Analysis (ELDA) of ex vivo limiting-dilution spheroid formation assay of dissociated tumor cells from human PDX models shown in Figure 6A, with plating densities at 1,000, 500, 250, 125, and 62.5 cells per well (n = 12 replicate wells per cell dosage)." (lines 910-913, page 39)

We have now included the TIC frequencies with 95% confidence intervals and likelihood-ratio test P-values from the extreme limiting dilution analysis (ELDA) in Supplementary Fig. 9A.

Sphere formation was monitored for 10–14 days, and wells containing spheres (>50 µm in diameter) were scored as positive. All sphere counting was performed by an investigator blinded to the experimental group assignments. Corresponding updates on plating conditions and scoring method have been included in Methods – *In vitro* limiting dilution tumorsphere assay (lines 590-597, page 27).

The IHC quantifications of p-IRE1α, PCNA, and TUNEL (Fig. 6F-H) are valuable for showing mechanism. The legend notes n=8 mice per group and one-way ANOVA; please clarify whether statistics were performed on per-animal means (i.e., multiple fields averaged per tumor) and whether field selection/scoring were blinded. If feasible, provide exact p-values (or maintain asterisks) and confirm that the same analysis pipeline was applied across markers.

Reply: A total of n = 8 mice per group were included in the *in vivo* experiment. For IHC staining and quantification of p-IRE1α, PCNA, and TUNEL, three mice per group were randomly selected. Field selection and scoring were performed in a blinded manner by two independent investigators to minimize bias. For each tumor sample, multiple representative fields (at least five fields per section) were captured, and the percentage of positive cells or staining intensity was averaged to obtain a per-animal mean. The same image-analysis pipeline and quantification criteria were applied consistently across all markers.

These experimental details have been added in Methods – Immunohistochemistry (IHC) (lines 676-686, pages 30-31). We have also updated the figures to include exact *P* values to enhance statistical transparency.

For the PDO experiments, the legend indicates two independent HCC organoid lines with a raw dose–response matrix (J). Please state explicitly that two PDOs were tested and report the synergy metric and score (e.g., Bliss or Loewe; values are shown in Supplementary synergy surfaces). Also indicate the number of biological replicates for the CellTiter-Glo viability assay and whether effects were consistent across both PDOs.

Reply: We have revised the figure legend for Fig. 6K to explicitly state that two independent PDO lines (labelled as Org#1 and Org#2) were tested (lines 915-918, page 39). The raw dose-response matrix data for the CellTiter-Glo viability assay were presented as a table for both individual HCC PDOs in Fig. 6L. The numbers within the table represent the mean organoid viability compared to DMSO control ± SD (n=3 replicates per condition).

Drug interaction was quantified using a dose-response matrix followed by SynergyFinder analysis, which revealed dose-dependent positive synergy predominantly at intermediate-to-high concentrations. Strong positive Bliss and ZIP scores (~35–46) were observed, with no evidence of antagonism across the tested PDOs (Fig. 6L; Supplementary Fig. 9C). Drug interaction effects were analyzed using the SynergyFinder platform (https://synergyfinder.fimm.fi) (Aleksandr Ianevski, *et al*. Nucleic Acids Res, 2022; PMID:

35580060), which computes synergy scores based on four established models: Highest Single Agent (HSA), Loewe, Bliss, and Zero Interaction Potency (ZIP). Both PDO lines exhibited consistent synergistic reductions in viability with combined HA15 and cisplatin treatment, though the magnitude of synergy varied slightly between organoid lines.

Corresponding updates have been added to Results (lines 417-420, page 16), New reference related to SynergyFinder platform has been cited in Ref. 36 (lines 1006-1008, page 41).

5. Interpretation of correlative clinical data (expression and patient outcomes): The manuscript integrates some clinical data (from patient cohorts and cell line databases) to support the experimental findings. These analyses are generally supportive but should be interpreted and described with care to avoid overstatement and to provide clarity on how they were done.

In Figure 1D, the authors show that HCC patients with high p38β (MAPK11) mRNA expression have significantly worse overall survival than those with low expression. This is an important correlation suggesting p38β is a negative prognostic factor. However, it's essential to specify how the "high" and "low" groups were defined. The caption or methods should state whether this was a median split, upper quartile vs lower quartiles, or an "optimal" cutoff determined by something like maximally selected rank statistics. The note that the high vs low groups were of unequal size (e.g., 41 high vs 60 low for p-BiP in Fig. 4E) suggests the authors may have chosen a cutoff that wasn't the median (possibly upper third). It would add rigor to report the hazard ratio (HR) and 95% confidence interval for high vs low p38β from a Cox proportional hazards model, along with the p-value.

Reply: We have now clarified the stratification methods and extended the statistical analyses as suggested. For the tissue microarray analysis shown in Fig. 1B-J, p38β expression was assessed by immunohistochemistry, and staining intensity was quantified using ImageJ software to generate H-scores. In-house HCC tumor samples were classified according to p38β expression relative to their matched adjacent non-tumor tissue. Cases in which the tumor H-score exceeded that of the corresponding non-tumor tissue were designated as "p38β-high", whereas those with lower tumor H-scores were defined as "p38β-low". Samples with equivalent H-scores between tumor and adjacent tissue were categorized as "no difference". This patient-specific comparative approach enables stratification based on relative tumor overexpression rather than relying on arbitrary percentile-based cutoffs. For the analysis of site-specific BiP Thr648 phosphorylation in the CPTAC HCC phosphoproteomic cohort (PDC000199) shown in Fig. 4G-I, quantification was based on the phosphopeptide LYGSAGPPPTGEEDtAEKDEL (where t denotes phosphothreonine) using log_2-normalized MS1 intensity values. Cases were stratified with values ≥ 0 defined as the high group ($n = 41$) and values < 0 as the low group ($n = 60$). This cutoff was chosen based on the distribution of log_2-transformed intensities, where zero represents the approximate median split.

Furthermore, we have now included Cox proportional hazards analyses to provide hazard ratios with 95% confidence intervals and associated p-values. For p38β expression in the in-house TMA cohort, the hazard ratio for high versus low expression is now reported in Fig. 1I-J. For BiP Thr648 phosphorylation in the CPTAC cohort, the corresponding Cox regression results are shown in Fig. 4H-I.

Corresponding result updates and patient stratification method have been made to Results and Methods – HCC patient stratification for clinicopathogological analyses, respectively (lines 133-136, page 6; lines 297-299, page 12; lines 720-731, page 32).

Figure 4E presents a survival analysis based on BiP Thr648 phosphorylation, a very interesting analysis that links the mechanism directly to clinical outcome. To strengthen this, please clarify

how p-T648 was measured (e.g., immunohistochemistry on a tissue microarray) and how "high" expression was defined (H-score threshold, percentage positivity, etc.). The text notes 41 vs 60 patients, suggesting a non-median cut-off (perhaps top 40%); please confirm. Reporting hazard ratios with 95% confidence intervals would also add rigour. In Figure 1J, the correlation between MAPK11 expression and cisplatin IC50 is statistically significant but modest (r ≈ –0.33) and should be described as a moderate association, not a strong one. Likewise, in Figure 2C, the higher MAPK11 expression in AFP-high tumours (p = 0.042) represents only a mild association, given the small effect size and the limitations of AFP as a stemness marker. Together, these findings are consistent with the overall model but should be presented with cautious wording to avoid overstating their strength.

Reply: As replied in the previous comment, for the analysis of site-specific BiP Thr648 phosphorylation in the CPTAC HCC phosphoproteomic cohort (PDC000199) shown in Fig. 4G-I, quantification was based on the phosphopeptide LYGSAGPPPTGEEDtAEKDEL (where t denotes phosphothreonine) using log_2-normalized MS1 intensity values. Cases were stratified with values ≥ 0 defined as the high group (n = 41) and values < 0 as the low group (n = 60). This cutoff was chosen based on the distribution of log_2-transformed intensities, where zero represents the approximate median split. Furthermore, we have now included Cox proportional hazards analyses to provide hazard ratios with 95% confidence intervals and associated p-values. For BiP Thr648 phosphorylation in the CPTAC cohort, the corresponding Cox regression results are shown in Fig. 4H-I.

We agree with the reviewer's comment that the correlation between MAPK11 expression and cisplatin IC50 (now Fig. 1K) is statistically significant but modest (r ≈ –0.33). We have revised the text to describe this as a moderate rather than strong association (lines 145, page 7). Similarly, the enrichment of MAPK11 in AFP-high tumors (now Fig. 1H, p = 0.042) is now described as a mild association (lines 132-133, page 6), with explicit acknowledgment of the limited effect size and the known constraints of alpha-fetoprotein as a reliable HCC stemness marker.

Minor Comments:

• Figure labelling & self-containment: Add/confirm axis titles, units, n's, and definitions of asterisks throughout. Fig. 2A: include a colour scale (log2 fold-change or z-score) and label a few sentinel genes (e.g., AFP, EPCAM, ALB). Fig. 2G: mark the kinase domain and TGY activation loop (T180/Y182). Fig. 4C–D: state clearly these panels derive from phosphoproteomics and clarify the plotted intensity units.

Reply: We have carefully reviewed all figures and added or confirmed axis titles, units, and revised *ns* to exact P-values throughout the manuscript. Figure legends have been updated accordingly to ensure consistency and clarity.

In Fig. 2A, the existing heatmap color bar has now been labeled to indicate Z-score values. Additionally, key sentinel genes, including AFP, EPCAM and ALB, have been highlighted in red to facilitate interpretation of the expression patterns.

In Fig. 2G (now Fig. 2E), the kinase domain and TGY activation loop (T180/Y182) of p38β have been clearly annotated in the figure (Jonah Beenstock, *et al*. J Biol Chem, 2014; PMID: 25006254). The figure legend has been updated to specify these structural features (lines 790-795, page 35).

In Fig. 4C, D (now Fig. 4A, B), we have clarified in the figure legend that these panels are derived from quantitative phosphoproteomic analysis. The revised legend now reads: "**A** MS/MS analysis of BiP Thr648 phosphorylation derived from phosphoproteomics Representative MS/MS spectrum of the phosphopeptide LYGSAGPPPTGEEDtAEKDEL (t

denotes phosphorylated threonine), confirming site-specific phosphorylation at Thr648. **B** Quantitative phosphoproteomic analysis showing log_2-transformed normalized MS1 intensities of the unique phosphopeptide containing BiP Thr648 across indicated samples." (lines 841-846, page 37).

• Survival plots (Figs 1D, 4E): In legends, include group sizes (n), data source (e.g., TCGA, IHC cohort), statistical test (log-rank), and—ideally—hazard ratio with 95% CI. Briefly state how "high/low" was defined (median, quantile, or optimised cut-off).

Reply: For Fig. 1D and Fig. 4E (now Fig. 4G), the tissue information, including the data source, group sizes, cut-off criteria, and statistical tests, has now been clearly described in the corresponding figure legends and Methods - HCC patient stratification for clinicopathological analyses. The hazard ratio for high versus low p38β expression is now reported in Fig. 1I-J. For BiP Thr648 phosphorylation in the CPTAC cohort, the corresponding Cox regression results are shown in Fig. 4H-I.

• Terminology consistency: Use "tumour-initiating capacity (TIC)" for function and "TICs" for the cells; prefer "self-renewal capacity" over broad "stemness" when referring to assays. Fix minor typos (e.g., "Tumour-initiating"). Define all abbreviations at first use (TIC, UPR, PDX, PDO, etc.).

Reply: We have carefully revised the manuscript to ensure consistent terminology throughout the manuscript. Specifically, we now use "tumour-initiating capacity (TIC)" when describing the functional property and "TICs" when referring to the cells. The term "self-renewal capacity" has been used in place of "stemness" when describing sphere-formation and limiting dilution assays. Minor typographical errors (e.g., "Tumour-initiating") have been corrected. In addition, all abbreviations, including TIC, UPR, PDX, and PDO, are now defined at their first appearance in the text. All corresponding changes have been highlighted in red in the revised manuscript.

• Tone/claims: Avoid superlatives unless effect sizes are large. Rephrase "strong" or "marked" effects as "moderate" or "significant" where appropriate (e.g., apoptosis decrease ~8–10%; p-PERK changes modest vs p-IRE1α).

Reply: We have carefully reviewed the manuscript and replaced the term "markedly" to "significantly" to more accurately reflect the observed effect sizes. All corresponding changes have been highlighted in red in the revised manuscript.

• Proteomics display (Fig. 3A): Replace or complement the hit list with a small volcano or enrichment bar plot highlighting BiP and known p38β partners (e.g., MAP2K3, MKNK1, DUSP9), and indicate replicate statistics/enrichment over control IP. In addition, all raw proteomics files (.raw) should be deposited in PRIDE (ProteomeXchange) and made publicly available with the corresponding accession number.

Reply: Tandem affinity purification coupled with mass spectrometry was performed in duplicate. The list of proteins identified as interacting with p38β was compared against proteins eluted from the empty vector control group. The complete set of unique binding proteins is now provided in Supplementary Table S4. Although only limited data, specifically the numbers of unique peptides and total peptides for each identified protein were available from the MS result, these metrics were used to generate a scatter plot of mean total peptides versus mean unique peptides (Fig. 3A; lines 223-225, pages 9-10). MS data are deposited in PRIDE: PXD067039.

• Graphical model (optional): A compact schematic of the p38β→BiP(T648)→IRE1α/PERK→UPR→TIC/chemoresistance axis would help readers and could double as a graphical abstract.

Reply: Please kindly refer to the following graphical abstract to illustrate the major findings in this study.

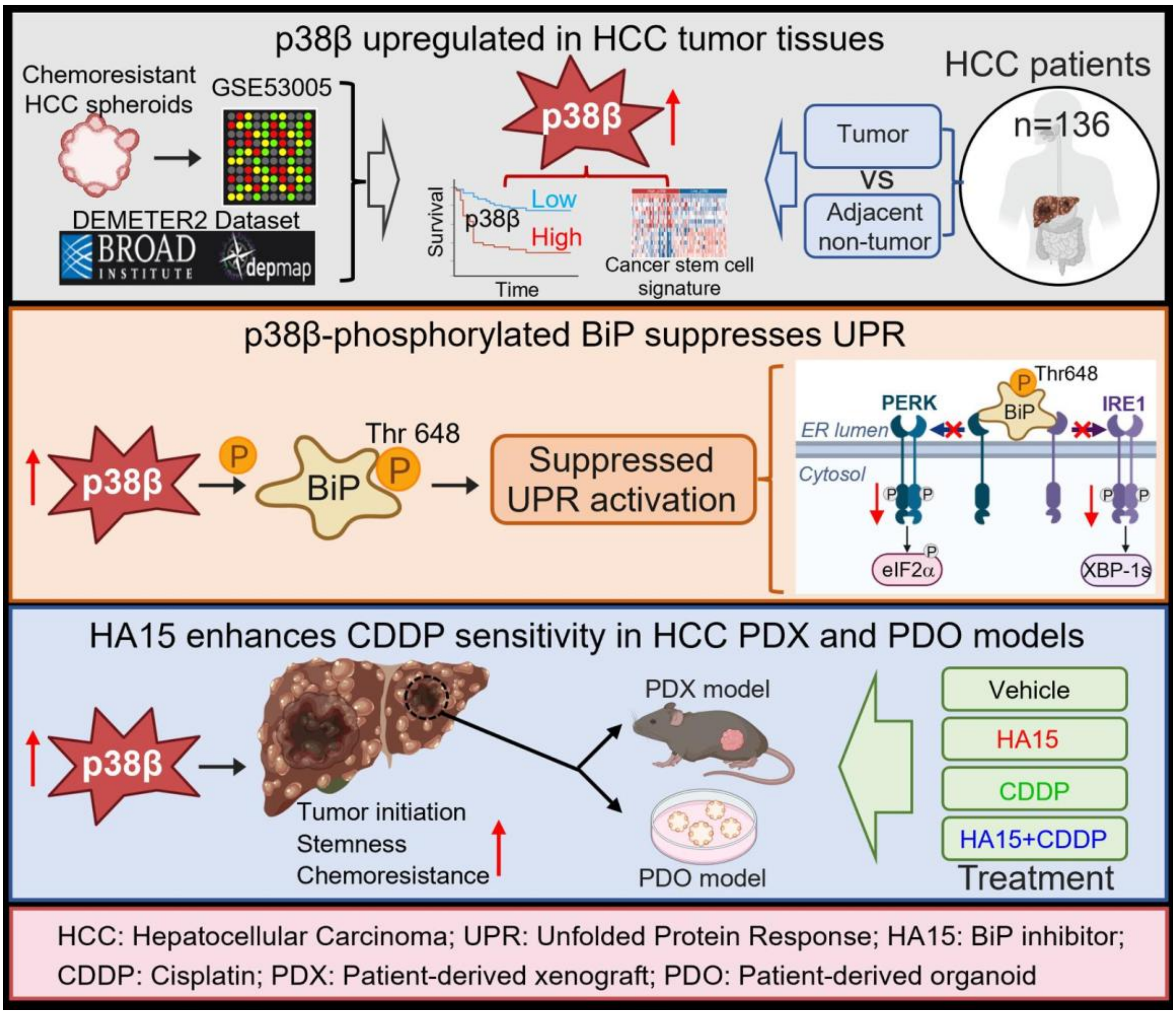

Reviewer #3 (Remarks to the Author):
In this manuscript, Huang et al. reported the identification of the stress kinase p38b as a putative regulator tumour-initiating cells and drug resistance in HCC. They proposed that p38b promotes BiP phosphorylation, increasing its association with the unfolded protein response sensors PERK and IRE1-a. Such modification suppresses unfolded protein response activation and reduces unfolded protein accumulation. Potential therapeutic relevance of these findings is proposed using PDX and tumoroid models. The manuscript is well structured and written, fluent in the way to document outcomes and strategies undertaken to address specific points. There are several concerns that should be addressed.

We thank the reviewer for the constructive comments. Please refer to our response to each specific comment.

Specifically:
1) Introduction: I strongly recommend to provide a better-deeper introduction about ER stress and UPR, rather than a simplistic overview.
Reply: We have revised Introduction to provide a more detailed and mechanistically grounded overview of ER stress and UPR signaling. Specifically, we have added the following text:
"As one of the most extensively characterized cellular stress–adaptive programs, the ER stress response enables cancer cells to survive under hostile microenvironmental conditions, including hypoxia, nutrient deprivation, and oxidative stress (Xi Chen, *et al*. Nat Rev Cancer, 2021; PMID: 33214692). Persistent ER stress activates the unfolded protein response (UPR), a tripartite signaling network centered on the ER transmembrane sensors PERK, IRE1α, and ATF6, which collectively coordinate translational attenuation, transcriptional reprogramming, and proteostasis restoration (David Ron, *et al*. Nat Rev Mol Cell Biol, 2007; PMID: 17565364; Claudio Hetz, *et al*. Nat Rev Drug Discov, 2013; PMID: 23989796; Peter Walter, *et al*. Science, 2011; PMID: 22116877; R Luke Wiseman, *et al*. Mol Cell, 2022; PMID: 35452616). Under homeostatic conditions, all three sensors are maintained in an inactive state through binding to the chaperone BiP/GRP78, positioning BiP as the master gatekeeper of UPR activation (A Bertolotti, *et al*. Nat Cell Biol, 2000; PMID: 10854322; David Ron, *et al*. Nat Rev Mol Cell Biol, 2007; PMID: 17565364; Peter Walter, *et al*. Science, 2011; PMID: 22116877). Upon stress-induced dissociation of BiP, PERK suppresses global protein synthesis via eIF2α phosphorylation while selectively inducing ATF4-dependent adaptive programs (H P Harding, *et al*. Mol Cell, 2000; PMID: 11106749); IRE1α initiates unconventional splicing of XBP1 to reinforce ER folding and lipid biosynthesis capacity (Marcella Calfon, *et al*. Nature, 2002; PMID: 11780124; Ann-Hwee Lee, *et al*. Science, 2008; PMID: 18556558); and ATF6 translocates to the Golgi to activate chaperone and ER-associated degradation pathways (Jingshi Shen, *et al*. Dev Cell, 2002; PMID: 12110171).
Importantly, UPR signaling displays a context-dependent duality in cancer. While sustained or excessive activation of PERK–CHOP or IRE1–JNK pathways can trigger apoptosis (Claudio Hetz, *et al*. Mol Cell, 2018; PMID: 29107536). In contrast, a finely tuned, adaptive UPR supports tumour cell survival by enforcing a low-protein-synthesis state, enhancing metabolic plasticity, and conferring resistance to cytotoxic stress—features that are particularly critical for TICs (Claudio Hetz, *et al*. Mol Cell, 2018; PMID: 29107536; Xi Chen, *et al*. Nat Rev Cancer, 2021; PMID: 33214692; Claudio Hetz, *et al*. Nat Chem Biol, 2019; PMID: 31320759). Recent studies further implicate UPR signaling in the maintenance of TICs by enhancing redox homeostasis, and rewiring transcriptional programs linked to stemness, plasticity, and immune evasion (Dong Liang, *et al*. Biochem Pharmacol, 2021; PMID: 34411568; Mohamed Ariff

Iqbal, *et al*. Aging Cell, 2024; PMID: 38757355; Claudio Hetz, *et al*. Mol Cell, 2018; PMID: 29107536). These observations underscore the UPR, particularly its BiP-centered regulatory node, as a critical stress-buffering system that may cooperate with oncogenic kinase signaling to sustain TIC phenotypes under therapeutic pressure." (lines 67-88, pages 4-5).

2) Western blot results should be supported by quantifications and statistical analyses performed using independent experiments (and graphs should be shown in Supplementary Figures).

Reply: We have now included quantitative analyses for all Western blot data, with densitometric intensities normalized to their respective loading controls.

3) The authors should corroborate their study by analysing p38b levels (and relevant signals involved in the circuit they identified) using available proteomic data from a HCC patient cohort.

Reply: We systematically surveyed all major publicly available large-scale HCC proteomic resources, including datasets from the Clinical Proteomic Tumor Analysis Consortium (CPTAC), the Human Protein Atlas, and other mass-spectrometry–based studies. Among these, the CPTAC dataset (PDC000199) provides the most comprehensive phosphoproteomic coverage; however, it does not contain protein-level quantification for MAPK11 (p38β) and phospho-specific information for MK2, PERK, or IRE1α. Importantly, we have already included a Kaplan-Meier analysis of overall survival in the HCC phosphoproteomic cohort (PDC000199) according to site-specific BiP phosphorylation at Thr648 in Fig. 4G.

To the best of our knowledge, no currently available HCC patient proteomic or phosphoproteomic dataset permits reliable analysis of p38β protein abundance or the specific phosphorylation states of the downstream UPR signaling components (e.g., PERK, IRE1α, or their direct targets) implicated in our proposed pathway. We will continue to monitor emerging proteomic resources and, where feasible, plan to validate these signaling relationships experimentally in additional patient-derived cohorts.

4) In the first section of results, using HCC spheroid cultures under repeated cisplatin and doxorubicin treatment, the authors must clarify which cells were used, how HCC spheroid cultures were established, and provide representative pictures in the Supplementary Figures, specifying which doses of cisplatin and doxorubicin were used. Moreover, they should show comparative drug dose responses of parental versus TIC-like cells, and western blots analysis (with quantifications) to confirm p38b enrichment in TIC-like versus parental cells.

Reply: We apologize for or the lack of clarity in the original manuscript. In this study, we used our previously published cDNA microarray profiling of chemoresistant hepatic tumorsphere (TIC-enriched) model generated by repeated cisplatin/doxorubicin selection (GSE53005) (Lee et al., Hepatology, 2014; PMID: 24523067) and identified differentially expressed kinase genes between chemoresistant hepatic tumorspheres and their differentiated counterpart. Briefly, tumour-initiating cells were enriched from HCC cell line PLC/PRF/5 cells by serial passages in serum-free spheroid culture supplemented with doxorubicin (2 mg/mL) and cisplatin (1 mg/mL), selecting for a self-renewing, chemoresistant population. The chemoresistant tumorsphere and their differentiated counterpart were subjected to cDNA microarray profiling. To avoid any potential misunderstanding, we have now revised Fig. 1A and made clarifications to Results (lines 111-116, page 6).

5) line 102-104: The authors stated that "p38β protein levels were markedly increased in multiple HCC cell lines relative to the normal liver cell line MIHA". However, the results reported in Fig. S1A do not support this statement, as p38b is overexpressed only in 2 cell lines, whereas slightly upregulated in 3/5 lines. Moreover, in relation to this and the pertinence of the cellular model used for studies, the authors performed most experiments using MHCC97L cells, with barely detectable levels of p38b. Findings should be supported using other cell lines, including intermediate (HepG2 and Huh7) and very high (PLC8024 and Hep3B) p38b levels. I also recommend to indicate in Figure panels which cell lines were used for different experiments, notably as there is no homogeneity on the cellular system used through studies.

Reply: We thank the reviewer for the careful critique and constructive suggestions. We have revised the text to more accurately describe the relative expression levels of p38β across HCC cell lines compared with the normal liver cell line MIHA. The revised sentence now states:
"p38β protein levels were moderately to highly upregulated in most HCC cell lines relative to MIHA, with the highest expression observed in PLC8024 and Hep3B cells." (lines 138-140, page 7).

We agree that findings should be supported across a range of endogenous p38β expression levels. We have already performed loss-of-function experiments by CRISPR-Cas9 mediated knockout of p38β in PLC8024 cells with high p38β expression (Supplementary Fig. 1A). Knockout of p38β resulted in reduced colony formation, increased cisplatin sensitivity, and suppressed tumorsphere formation (Fig. 1L-N, Supplementary Fig. 2C).

In addition to the original results using MHCC97L (low p38β) and PLC8024 (high p38β) cells, we have now extended our core functional assays to an additional HCC cell line, Huh7, which exhibits relatively low endogenous p38β expression. Consistent with the results observed in MHCC97L, overexpression of p38β WT, but not p38β Mut in Huh7 cells, significantly promoted colony formation, sphere formation, and resistance to cisplatin (Supplementary Fig. 3D-G; lines 197-199, page 9). These results confirm that the oncogenic effects of p38β are reproducible across multiple HCC cell lines.

We have now clearly labeled the specific cell lines used for each experiment directly within the figure panels and have ensured consistent annotation in the corresponding figure legends throughout the manuscript. This should resolve any ambiguity regarding the cellular system used across different experiments.

6) Figure 1H-K: A large set of studies to assess the functional requirement of p38b has been essentially performed in PLC8024 HCC cells, in which p38b is overexpressed, thus possibly representing a specific signalling context. Moreover, cells have not been treated with chemotherapeutic, therefore the chosen model exemplify the requirement of p38b in HCC and not in relation to acquired chemo-resistance following treatments. Studies should be complemented with cellular settings modelling resistance acquisition following drug-treatment.

Reply: We would like to clarify as above that CRISPR-mediated knockout of p38β was performed using PLC8024 cells expressing high level of p38β. flow cytometry analysis has already demonstrated a robust increase in Annexin V-positive apoptotic cells following CDDP treatment in p38β-deficient cells compared to controls (Fig. 1N). We have now repeated the foci-formation assay under cisplatin (CDDP) treatment. The result showed that knockout of p38β reduced foci-forming capacity compared with control cells following CDDP exposure (Fig. 1M). Collectively, these results support a functional requirement for p38β in HCC cells under chemotherapeutic stress.

Corresponding updates have been made to Results (lines 151-152, page 7).

7) In Figures reporting patient data (e.g. Fig 1B-G, 2C-E, ...), I recommend to indicate the cohort used for analysis in panels (or in Figure legends).

Reply: Thank you for the suggestion. We have now supplemented the cohort information for patient-based analyses in the corresponding figure legends (Fig. 1B-H, lines 762-769, page 34; Fig. 2A-D; lines 784-790, page 35; Fig. 4G-I, lines 854-861, page 37).

8) The graph in Figure 4B reports a reduced CDDP sensitivity of about 5%, thus documenting a marginal rather than a "markedly reduced» sensitivity. Additional analyses are needed to strengthen the conclusion. As above, findings should be supported using other HCC cellular models.

Reply: We agree that the change in CDDP sensitivity detected by the short-term apoptosis assay appears modest (~10%) in Fig. 4B (now Supplementary Fig. 3A). As short-term apoptosis assays mainly capture acute cytotoxic responses within 24-48 hours and may not fully reflect long-term survival under chemotherapeutic pressure, we employed additional assays capable of assessing cumulative drug tolerance. Clonogenic growth and *in vitro* limiting-dilution tumorsphere assays were therefore performed under CDDP treatment. Consistently, p38β WT expression led to a pronounced increase in colony-forming efficiency and sphere-initiating capacity relative to EV or Mut controls (Supplementary Fig. 3B, C), demonstrating a significant functional contribution of p38β to chemoresistance. Together, these results indicate that although the short-term apoptotic response is modest, the long-term clonogenic survival and sphere-initiating capacity of p38β-expressing cells are significantly enhanced, supporting a key role for p38β in driving CDDP resistance in HCC.

Corresponding updates have been made to Results (lines 194-197, page 8-9).

9) line 289: The authors claimed that they used PDX and PDO from advanced HCC specimens exhibiting high p38b expression. Western blot analyses should document this statement.

Reply: We apologize for the lack of clarity in the original manuscript. We have already performed Western blot analyses to document p38β expression levels in the PDX and PDO specimens derived from advanced HCC patients. These data are presented in Fig. 3F. Specifically, Patient #1, #2, and #3, which exhibit high p38β expression, correspond to PDO Org#1, PDO Org#2, and PDX#1, respectively. Patient #1 with low p38β expression corresponds to PDX#2.

10) line 292-294: The authors reported that "As expected, CDDP monotherapy moderately inhibited tumor growth, whereas the combination treatment led to a significantly greater reduction in both tumor volume and weight compared to CDDP alone". However, results reported in Fig. 6A-C show no significant difference between CDDP alone versus CDDP+HA15. Considering the different capability of cells shown in ex vivo limiting dilution spheroid formation assay (Figure 6D), it is possible that the addition of HA15 to CDDP essentially depletes self-renewal capacity of HCC cells, without impacting tumour growth. Additional experiments should strengthen this hypothesis, for example: a) assessing tumour evolution over time after stopping the treatment in mice; b) using cells from treated tumours not only for ex vivo limiting spheroid formation assays, but as well for new in vivo xenograft studies. Moreover, cancer stem cell like markers from these tumours should be reported.

Reply: We thank the reviewer for this insight interpretation and constructive suggestions. We agree that the modest effect on endpoint tumor volume and weight (Fig. 6A-C) does not fully capture the functional impact of HA15 on self-renewal property. To address this, we have performed the additional experiments recommended by the reviewer.

We repeated the *in vivo* treatment experiment with a drug-withdrawal design. Briefly, mice bearing HCC patient-derived xenografts (PDXs) were treated for 2 weeks with vehicle, HA15 alone, CDDP alone, or CDDP+HA15, after which treatment was withdrawn and tumors were monitored longitudinally. CDDP alone produced moderate suppression of tumor growth during the treatment phase. However, upon treatment withdrawal and longitudinal follow-up, the CDDP+HA15 combination group exhibited significantly more durable suppression of tumor regrowth following treatment withdrawal, indicating impaired tumor re-initiation capacity (Fig. 6D).

To directly assess the functional self-renewal capacity of residual tumor cells, we performed secondary implantation. Residual tumor cells harvested from each treatment group were transplanted into new recipient mice. Mice receiving cells from the CDDP+HA15 combination group showed significantly longer tumor-free survival compared to those receiving cells from the CDDP-alone group (Fig. 6E). We also profiled CSC surface markers in tumors harvested from each treatment group. Flow cytometry analysis revealed a reduced proportion of cells positive for the CSC markers CD133 and EpCAM in the combination group compared to CDDP alone (Supplementary Fig. 9B).

Together, these results support the interpretation that HA15 and CDDP combination treatment reduces TICs self-renewal ability, which is more faithfully captured by tumor regrowth kinetics and secondary transplantation assays than by endpoint tumor volume measurements within the on-treatment window.

These new findings have been added to Results (lines 401-405, 408-410, page 16).

11) Based on tumoroid studies, the authors claimed that there is "a strong synergistic interaction" between CDDP and HA15, although synergy is essentially observed at high doses. Findings should be corroborated using additional tumoroids, and importantly using as well liver organoids to assess toxicity effects.

Reply: We agree that the CDDP–HA15 interaction is dose-dependent and that synergy is most apparent at intermediate-to-high concentrations. We therefore revised the wording from "strong synergy" to "dose-dependent positive synergy predominantly at intermediate-to-high concentrations" (lines 418-419, page 16). Consistently, SynergyFinder analysis shows positive synergy in this range (Bliss/ZIP scores ~35–46) across the tested PDOs, with no evidence of antagonism (Supplementary Fig. 9C).

To assess the safety of the combination treatment, we have extended the combination testing to normal human liver epithelial cell lines MIHA and THLE-3, in which viability was only modestly reduced (remaining >50%) within the synergy-associated concentration range (e.g., CDDP 10 μM + HA15 5 μM) (Supplementary Figure 10A, B). Moreover, treatment in normal human hepatocyte-derived organoids showed no overt hepatotoxicity at synergy-associated concentrations, supporting a favorable therapeutic window (Supplementary Figure 10C).

These new findings have been added to Results and Methods – Cell lines, PDOs and PDX (lines 420-428, pages 16-17; lines 546-547, 556-558, page 22).

12) In consideration to the current HCC treatment essentially based on ICI and RTK inhibitors, the authors should discuss the relevance of their findings in the context of treatments other than with CDDP, and in relation to the still putative relevance of CDDP for liver cancer therapy (citing recent and pertinent references, e.g. PMID: 37446035).

Reply: Thank you for this important suggestion. We have revised the Discussion accordingly. The revised text also explicitly addresses the ongoing clinical relevance of cisplatin in

locoregional and combination regimens, citing the recommended reference (PMID: 37446035). The updated Discussion section now reads as follows:

"We extend this knowledge by demonstrating that its phosphorylation status critically shapes UPR signaling outcomes and thereby modulates TIC-associated stress tolerance. In the current therapeutic era, first-line systemic treatment for advanced HCC is largely based on immune checkpoint inhibitor– and multikinase/RTK inhibitor–containing regimens, such as atezolizumab plus bevacizumab or durvalumab plus tremelimumab, with TKIs including sorafenib and lenvatinib remaining important options in selected patients (John D Gordan, *et al*. J Clin Oncol, 2024; PMID: 38502889; Khi Yung Fong, *et al*. Liver Cancer, 2023; PMID: 36872922). Although systemic cisplatin monotherapy is no longer a standard first-line approach, cisplatin remains clinically relevant through its central role in conventional and drug-eluting TACE, cisplatin-based HAIC protocols, and emerging sequential or combination strategies with TKIs and ICIs in unresectable or refractory disease (Sae Hamaya, *et al*. Int J Mol Sci, 2023; PMID: 37446035; Tengfei Si, *et al*. Front Bioeng Biotechnol, 2022; PMID: 36237208). Notably, cisplatin, multikinase inhibitors, and immune-mediated cytotoxicity can all impose substantial ER stress on tumour cells, and ER stress/UPR signaling is increasingly recognized as a determinant of HCC progression and therapeutic resistance (Hery Urra, *et al*. Trends Cancer, 2016; PMID: 28741511; Aleksandra Mandic, *et al*. J Biol Chem, 2003; PMID: 12509415; Ying-Hong Shi, *et al*. Autophagy, 2011; PMID: 21691147) . Our data indicate that the p38β–BiP axis functions as a core ER stress–adaptive module that enables TICs to withstand proteotoxic pressure. Thus, beyond explaining cisplatin tolerance, this pathway may represent a broader TIC survival program engaged across multiple treatment modalities in the current ICI/TKI-based treatment landscape.

Given this broader role of the p38β–BiP axis in buffering ER stress across diverse therapeutic pressures, we next assessed whether pharmacological disruption of BiP could translate into tangible therapeutic benefit." (lines 494-512, page 20).

13) Some material and method parts must be implemented. For example, concerning "in vitro limiting dilution tumorsphere assay": which medium was used to perform the assays? After how many days samples were processed and quantified? Moreover, drug doses used in all experiments must be reported in figure legends or panels, notably as apparently different according to experiments performed.

Reply: Thank you for the suggestions. We have now revised the Methods - *In vitro* limiting dilution tumorsphere assay accordingly (lines 590-597, page 27).

"Cells were seeded at densities of 100, 50, 25, 10, and 5 cells per 100 µL in PolyHEMA-coated 96-well plates using DMEM medium supplemented with 0.25% methylcellulose, insulin (4 µg/mL), B27 (1X), EGF (15 ng/mL), and FGF (20 ng/mL). Sphere formation was monitored for 10–14 days, and wells containing spheres (>50 µm in diameter) were quantified using ELDA software (https://bioinf.wehi.edu.au/software/elda/) to calculate stem cell sphere-forming frequency."

In addition, we have now carefully reviewed all experiments involving drug treatments and have standardized the reporting of drug concentrations and treatment durations. All drug concentrations and treatment durations are now clearly indicated in the corresponding figure legends.

Reviewer #4 (Remarks to the Author)

The fundamental relevant observation of this work is that Bip is phosphorylated at threonine 648 (Thr648) (Figures 4C,D), near the C-terminus of the protein, a modification not previously linked to p38β signaling. This phosphorylation site is novel and given the large number of known BiP interactors this point itself is important and at the same time – surprising. The current work links it by several experiments to the oncogenic phenotypes, showing that expression of TA648 mutated BiP increased significantly the tumor-initiating frequency and tumor burden (Fig. 4 panels). Collectively, the authors suggest based on their findings that BiP Thr648 phosphorylation is required for the observed p38β-driven stemness and drug resistance. The manuscript claims that the authors have previously demonstrated that the novel phosphorylation is due to the p38β kinase, and that the kinase is stress-induced, though this important and novel point is not elaborated and data supporting these claims are thin in the current data set.

We thank the reviewer for the constructive comments. Please refer to our response to each specific comment.

These data include exclusive reliance on western blots; more direct gels that report on phosphorylation (e.g. Phostag gels) are absent. There is also only cursory characterization of the antibodies used; This is pertinent as multiple Isoforms of various HSP70 isoforms exist and the current work does not show the BiP is specifically the recipient of the phosphorylation.

Reply: Thank you for the comments. We further employed phosphate-affinity (Phos-tag) SDS-PAGE to directly visualize phosphorylation-dependent mobility shifts of BiP in p38β-overexpressing MHCC97L cells. BiP displayed a significantly slower-migrating band in p38β-overexpressing cells compared with EV and Mut, consistent with the expected shift caused by phosphorylation (Fig. 4C). To validate whether BiP is directly phosphorylated by p38β, an *in vitro* kinase assay using an activated recombinant p38 β and BiP proteins demonstrated that p38β could directly phosphorylate BiP at Threonine residue (Fig. 4D). Consistently, BiP immunoprecipitation followed by immunoblotting with a pan-phospho-Threonine antibody revealed increased threonine phosphorylation of immunoprecipitated BiP upon p38β WT overexpression, but not upon overexpression of the kinase-dead Mut (Fig. 4E).

To address potential antibody cross-reactivity within the HSP70 family, we examined if HSPA8 as an abundant HSP70 isoform interacts with p38β. In contrast to BiP, p38β could not be co-immunoprecipitated with HSPA8 (Supplementary Fig. 6E), and p38β WT overexpression did not measurably increase threonine phosphorylation of HSPA8 (Supplementary Fig. 6D), supporting that the p38β-dependent phosphorylation event is selective for BiP rather than a general effect on HSP70 proteins.

Corresponding updates have been made to Results (lines 274-284, 290-294, pages 11-12).

A second and third major issues are that most, if not all the evidence is derived from a) the use of cell lines (e.g. MHCC97L cells) and b) is based on over- and under-expression in them. Even the in vivo models involve animals in which the in vitro-altered cells affect tumor cell properties after implantation. There is no verification of the interventions used, for example via endogenous specific gene disruption. Thus, the experimental system is inherently complex the biochemical characterization is vague and is not backed up by in vivo experiments that cleanly show the novel phosphorylation of BiP (rather than a related HSP70) by p38β.

Reply: Thank you for the comment. To further demonstrate that the interaction between p38β and BiP is specific *in vivo*, we have now incorporated an additional mouse model. Using adeno-

associated virus serotype 8 (AAV8)-mediated transduction of wild-type (WT) p38β, kinase-dead mutant (Mut) p38β, or empty vector (EV) into human HCC PDX, we showed a stronger tumorigenic potential in p38β WT-overexpressed tumors (Fig. 2O-P) and an enhanced tumorsphere initiating capacity of resected p38β WT-overexpressed tumor cells (Fig. 2Q; Supplementary Fig. 4I). IHC staining of AAV8-transduced PDX tumors confirmed that p38β WT overexpression resulted in increased proliferative activity, as indicated by elevated PCNA staining in p38β WT group compared with EV and Mut groups (Supplementary Fig. 4J-L). In addition, *in vivo* delivery of AAV8-p38β-WT into established HCC PDX tumors resulted in detectable threonine phosphorylation of immunoprecipitated BiP, which was significantly reduced in tumors expressing kinase-dead p38β Mut or EV (Fig. 4F).
These new findings have been added to Results (lines 210-217, page 9; lines 284-286, page 12).

The bulk of other data is concerned with the effects of the phosphorylation event on downstream UPR sensors and on consequent signaling and tumor biology. These data are extensive but, in my opinion, the central conclusion that "BiP phosphorylation status critically shapes UPR signaling outcomes" is not sufficiently supported by the data for the reasons detailed above.
Reply: The additional experiments described above, including Phos-tag SDS-PAGE, *in vitro* kinase and binding assays, detection of phospho-Thr on immunoprecipitated BiP, HSPA8 immunoprecipitation, *in vivo* AAV8-mediated p38β overexpression, and detection of endogenous BiP phosphorylation, collectively demonstrate that the p38β–BiP phosphorylation axis is functionally specific both *in vitro* and *in vivo*. Furthermore, examining downstream UPR effectors (CHOP and XBP-1s) in cells overexpressing or lacking p38β, as well as in cells expressing BiP T648 mutants or with BiP silencing/inhibition (Fig. 5B, E, H; Supplementary Fig. 8A, E), yielded consistent results showing that p38β-dependent BiP phosphorylation regulates UPR signaling. Taken together with our original findings and these new data, we believe that the evidence robustly supports the conclusion that the p38β–BiP phosphorylation axis controls downstream UPR signaling.

Reviewer #5 (Remarks to the Author)

General assessment

This manuscript identifies p38β (MAPK11) as a driver of chemoresistance and tumor-initiating capacity in hepatocellular carcinoma (HCC) via phosphorylation of BiP at Thr648, suppression of the unfolded protein response (UPR), and maintenance of stemness. The authors provide a comprehensive dataset integrating screening, phosphoproteomics, functional assays, and validation in PDX/PDO models. The translational relevance is highlighted by pharmacological targeting with HA15.

The work is innovative and of potential impact. However, several critical gaps remain that need to be addressed to consolidate the mechanistic claims and ensure reproducibility.

We thank the reviewer for the constructive comments. Please refer to our response to each specific comment below.

Major comments

1. Isoform specificity of p38β

Current experiments focus solely on p38β. Although p38β ranked highest in the DEMETER2 RNAi screen, other isoforms (p38α/γ/δ) were not examined. No data compare whether the observed effects are unique to p38β. This leaves open the possibility of redundancy. Additional knockdown/overexpression or pharmacological inhibition experiments are needed to establish isoform specificity.

Reply: Thank you for the comment and suggestion. To directly address potential isoform redundancy, we selectively silenced other p38 MAPK family members. Knockdown of p38γ (MAPK12), or p38δ (MAPK13) failed to alter foci formation and CDDP sensitivity in PLC8024 cells, whereas knockdown of p38α (MAPK14) showed a slight inhibitory effect on both phenotypes (Supplementary Fig. 1D-I), underscoring a non-redundant dependence on p38β. In parallel, we performed pharmacological inhibition using the p38α/β inhibitor SB203580 in MHCC97L cells engineered with p38α silencing and p38β WT overexpression. SB203580 treatment significantly reduced tumorsphere formation in p38β WT overexpressing cells with p38α silencing, but not in empty vector (EV) control (Supplementary Fig. 2A, B). Collectively, these genetic and pharmacological data support a non-redundant, isoform-specific role of p38β in sustaining TIC-associated stemness and chemoresistance.

These new findings have been added to Results (lines 152-157, page 7; lines 173-179, page 8).

2. Direct substrate validation of BiP Thr648

The authors detected BiP Thr648 phosphorylation in phosphoproteomics and confirmed the site by MS/MS. Functional rescue with T648A and T648D mutants further supports its role. However, the causal link between p38β activity and BiP Thr648 phosphorylation remains indirect. Stronger evidence could be provided by demonstrating loss of Thr648 phosphorylation following genetic or pharmacological inhibition of p38β, or by generating endogenous knock-in BiP mutants (T648A/D) to validate physiological relevance.

Reply: We thank the reviewer for this important mechanistic point. To directly establish BiP Thr648 as a phosphorylation substrate of p38β, we performed four additional lines of validation: (1) *In vitro* kinase assay: Purified recombinant p38β directly phosphorylated recombinant BiP, demonstrating that no other cellular components are required for this modification (Fig. 4D; lines 278-281, page 11);

(2) Cellular p38β activity dependence: Overexpression of wild-type (WT) p38β followed by BiP immunoprecipitation yielded a strong phospho-threonine signal, whereas the kinase-dead mutant (Mut) showed minimal phosphorylation (Fig. 4E; lines 281-284, pages 11-12).
(3) *In vivo* tumor context validation: AAV8-mediated delivery of p38β-WT into HCC patient-derived xenograft (PDX) tumors robustly induced BiP threonine phosphorylation, while phosphorylation was significantly reduced in empty-vector (EV) and kinase-dead p38β mutant groups (Fig. 4F; lines 284-286, page 12), confirming physiological relevance and p38β kinase dependence under native tumor conditions.
(4) Functional rescue with BiP phospho-mutants: In the context of p38β overexpression, the phosphorylation-deficient BiP T648A (TA) mutant abrogated, whereas the phosphomimetic BiP T648D (TD) mutant phenocopied, the p38β-dependent suppression of UPR sensor phosphorylation and downstream UPR effectors (Fig. 5H; lines 372-378, page 15). These data link p38β-mediated BiP Thr648 phosphorylation to downstream UPR regulation. Importantly, across all assays, BiP phosphorylation strongly correlated with p38β activity, demonstrating that Thr648 is a direct and functional substrate of p38β.

3. Exclusion of other candidate genes

The initial screen identified four additional candidates (TYK2, FGFR4, PRKD2, SRPK3). These are listed but not functionally tested. Without even minimal validation, it remains uncertain whether their contribution to chemoresistance and TIC maintenance has been excluded. Comparative knockdown assays (±cisplatin, sphere formation, UPR readouts) would help rule out alternative explanations and reinforce the uniqueness of p38β.

Reply: In our initial analysis of kinome-focused transcriptomic profiling of chemotherapy-enriched, TIC-like HCC spheroids with DepMap (DEMETER2) dependency data, p38β emerged as the strongest candidate. It also showed the largest expression fold-change between HCC tumor and non-tumor samples from TCGA-LIHC database, providing a rationale for prioritizing it for further investigation.
Although TYK2, FGFR4, and PRKD2 were also recovered in this analysis, each has been previously implicated in therapy response or stemness in other tumour contexts. TYK2 promotes survival via BCL-2/MCL-1 and its silencing enhances chemosensitivity (Catarina R Carmo, *et al*. PLoS One, 2011; PMID: 21625473), while predictive modelling has associated high TYK2 expression with lower IC_{50} values for cisplatin and paclitaxel (Yaodong He, *et al*. Front Genet, 2022; PMID: 36531252). FGFR4 has been linked to 5-FU/oxaliplatin resistance in colorectal cancer (R C Turkington, *et al*. Cell Death Dis, 2014; PMID: 24503538) and doxorubicin resistance in breast cancer (Min Xu, *et al*. Cell Physiol Biochem, 2018; PMID: 29763898), and FGF19–FGFR4 signalling has been further associated with sorafenib resistance in HCC (Lixia Gao, *et al*. J Exp Clin Cancer Res, 2017; PMID: 28069043). Likewise, PRKD2 has been reported to contribute to chemoresistance in AML (Qian Li, *et al*. Onco Targets Ther, 2019; PMID: 31849496) and cervical cancer models (Ruijing Feng, *et al*. Curr Cancer Drug Targets, 2023; PMID: 36017858). By contrast, we found no clear evidence linking SRPK3 to chemoresistance to date, although its potential role in HCC remains of interest. SRPK3 is therefore being explored as a separate project in our laboratory; however, those data are still under active investigation and cannot be included in the present study. Given this landscape, our aim was to define a previously unrecognized kinase axis governing TIC maintenance and chemoresistance in HCC. MAPK11/p38β uniquely fulfilled this criterion and, notably, lacked any known mechanistic connection to BiP–UPR signalling. We therefore focused our efforts on elucidating the basis of p38β-driven chemoresistance.

4. Comprehensive UPR readouts

Mechanistic experiments mainly assess p-PERK, p-IRE1α, and unfolded protein accumulation by TPE-MI. ATF6 was reported as undetectable, but other canonical UPR markers (ATF4, CHOP, phospho-eIF2α, spliced XBP1) were not measured. Including these readouts would strengthen the claim that p38β–BiP signaling broadly suppresses the UPR.

Reply: Thank you for the suggestion. We have expanded our analyses to include additional canonical UPR markers, including phospho-eIF2α, ATF4, CHOP, and spliced XBP1, as shown in Fig. 5B, E, H and Supplementary Fig. 8A, E.

(1) Knockout of p38β resulted in a coordinated increase in phospho-PERK, phospho-eIF2α, ATF4, and CHOP, together with a significant elevation in spliced XBP1 (Supplementary Fig. 8A), indicating activation of both the PERK-eIF2α-ATF4 and IRE1α-XBP1 branches of the UPR (lines 334-337, page 13).

(2) Conversely, ectopic overexpression of p38β WT, but not the kinase-dead Mut, particularly under CDDP treatment, suppressed phosphorylation of PERK, eIF2α, and IRE1α, and reduced the levels of ATF4, CHOP, and spliced XBP1(Fig. 5B), confirming that p38β activation restrains global UPR signaling (lines 328-333, page 13).

(3) Genetic silencing or pharmacological inhibition of BiP by HA15 in p38β overexpressing cells, as well as the phosphorylation-deficient BiP T648A mutant, abrogated the effects of p38β overexpression (Fig. 5E, H; Supplementary Fig. 8E), supporting that BiP acts downstream of p38β in mediating UPR activation (lines 354-358, 362-365, page 14; lines 372-378, page 15).

Taken together, these findings demonstrate that p38β-mediated BiP activity functions as a central brake on both the PERK-eIF2α-ATF4 and IRE1α-XBP1 branches of the UPR, thereby conferring broad suppressive control over UPR signaling.

5. Clinical data analysis

Patient cohort data are analyzed by correlation tables and Kaplan–Meier survival curves. However, no multivariate Cox regression was presented to confirm whether p38β (or p-BiP T648) is an independent prognostic factor after adjustment for TNM stage, vascular invasion, AFP, etc. Such analysis is required to establish prognostic independence.

Reply: Thank you for the comment. We have now included multivariate Cox proportional hazards analyses incorporating key clinicopathological covariates, including TNM stage, vascular invasion, serum AFP level, tumor size, and number of nodules in the revised manuscript. The results demonstrate that both high p38β expression and elevated p-BiP Thr648 levels remain significantly associated with poor overall survival after adjustment for these variables, indicating that they serve as independent prognostic predictors of adverse outcome (Fig. 1I, J and Fig. 4H, I).

Descriptions have been made to Results (lines 133-136, page 6; lines 297-299, page 12).

Minor comments

1. Provide more detail on phosphoproteomic data analysis (localization probability thresholds, FDR, multiple testing correction) and make raw spectra available as supplementary data.

Reply: MHCC97L cells co-expressing SFB-tagged BiP with p38β WT, kinase-dead p38β Mut, or empty-vector (EV) controls, were immunoprecipitated with streptavidin beads for SFB-tagged BiP pull-down. SFB-tagged BiP immunoprecipitates were subjected to SDS-PAGE and Coomassie Blue staining, using a single biological replicate per condition. The gel lanes were separately cut into slices and subjected to in-gel digestion. The tryptic peptides were subjected to phosphoproteomic profiling by LC-MS/MS.

Raw mass spectrometry data were processed using MaxQuant 1.6.0.13. Raw data was searched against Human UniProt FASTA database (April 2020) containing 31, 165 entries, and also targeted HSPA5/BiP targeted full length protein sequence, using settings as below: oxidized methionine (M), acetylation (Protein N-term) were selected as dynamic modifications, and Phospho STY, carbamidomethyl (C) as fixed modifications with minimum peptide length of 7 amino acids was enabled. Confident proteins were identified using a target-decoy approach with a reversed database, strict false-discovery rate 1% at peptide and peptide spectrum matches (PSMs) level; minimum ≥2 unique peptides, ≥2 peptide spectral matches (PSMs), localization probability score > 0.75, Delta score > 80. All identified BiP phosphorylation sites and associated quantitative values are provided in Supplementary Table S5, and the raw MS/MS data have been deposited in the PRIDE repository (accession PXD067039).

The above update has been made to Methods - Phosphoproteomic analysis by LC–MS/MS (lines 654-675, pages 29-30).

2. Include baseline clinical and demographic characteristics of the PDX donor patients, including age, sex, disease stage, treatment history, and race/ethnicity, so that readers can better appreciate the representativeness of the models used.

Reply: Clinicopathological characteristics of the PDX donor patients are summarized in Supplementary Table S2.

3. Ensure transparent reporting of sample sizes, replicates, effect sizes, and exact p-values in figure legends, specifying whether randomization and blinding were applied in PDX studies.

Reply: In the revised manuscript, all figures and figure legends have been standardized to transparently report sample size (n), number of replicates, statistical tests, and exact p-values.

For PDX experiments, mice bearing PDX were randomly allocated to treatment groups (n = 8 per group). Tumor measurements and endpoint assessments were conducted in a non-blinded manner.

These details are now explicitly stated in the corresponding figure legends and in Methods - Pharmacological treatment and intratumoral AAV8 delivery in HCC PDX (lines 695-704, page 31).

4. Expand the discussion to differentiate p38β-specific biology from the better-studied p38α isoform, citing prior literature.

Reply: In the revised Discussion, we have now expanded the section comparing p38β (MAPK11) with the more extensively studied p38α (MAPK14), emphasizing their distinct biological functions, substrate specificities, and tissue distributions. Specifically, we have added the following text:

“Although p38α is the best-characterized p38 MAPK isoform and a canonical mediator of cellular stress signaling (Ana Igea, *et al*. Cancer Res, 2015; PMID: 26377941. Ignacio Dolado, *et al*. Cancer Cell, 2007; PMID: 17292829), its functions in cancer are highly context dependent. p38α activation can enforce tumor-suppressive programs, such as p53-dependent cell-cycle arrest (Fang Zheng, *et al*. J Exp Clin Cancer Res, 2014; PMID: 24766860), senescence (Konstantin Slobodnyuk, *et al*. Cell Death Dis, 2019; PMID: 31092814), and DNA-damage responses (Begoña Cánovas, *et al*. Cancer Cell, 2018; PMID: 29805078), and genetic deletion of p38α accelerates tumour initiation in multiple tissues (Lijian Hui, *et al*. Nat Genet, 2007; PMID: 17468757). Conversely, in established cancers, p38α can promote metabolic adaptation, invasion, and therapy resistance, reflecting its ability to support tumour survival under chronic stress (Ana Igea, *et al*. Cancer Res, 2015; PMID: 26377941). Given this duality, recent studies

have emphasized the importance of isoform-specific dissection of p38 MAPK biology, as many phenotypes historically attributed to "p38 MAPK" are now recognized to be isoform-selective and not exclusively driven by p38α. In contrast to p38α, p38β possesses distinct biochemical and regulatory properties, including cis-autophosphorylation, prolonged activation under chronic stress, and divergent substrate and scaffolding interactions (Jonah Beenstock, *et al*. J Biol Chem, 2014; PMID: 25006254. Wang Yuan, *et al*. Nat Commun, 2023; PMID: 37507441. Qiang Yuan, *et al*. Nat Commun, 2025; PMID: 40195298.). These differences suggest non-redundant signalling architectures, yet the contribution of p38β to cancer biology has remained largely unexplored. Prior studies have implicated p38β—not p38α—in selective stress-response programs, but its role in tumour-initiating cell (TIC) maintenance has been unknown."
The above update has been added to Discussion (lines 439-455, page 18).

Summary recommendation
The manuscript is promising but requires major revision. Specifically, demonstration of p38β isoform specificity, stronger evidence linking BiP Thr648 phosphorylation to p38β activity, minimal functional testing of other candidate genes, expansion of UPR readouts, and multivariate survival analysis are necessary. Addressing these points will significantly strengthen the mechanistic depth, originality, and translational relevance of the study.
Reply: We thank the reviewer for the thoughtful summary and constructive recommendations. We have carefully addressed each of the major points raised through the additional experiments and data described in our point-by-point responses above. We believe that these revisions, together with those made in response to the other reviewers, have substantially strengthened the mechanistic depth, originality, and translational relevance of the study. We are grateful for the reviewer's guidance in improving our manuscript.

Reviewer #1 (Remarks to the Author):
The revised manuscript satisfactorily addresses all of my previous concerns, primarily through the inclusion of additional data that strengthen the authors' conclusions. I therefore recommend publication.
Reply: We sincerely thank the reviewer for the positive evaluation and recommendation for publication.

Reviewer #2 (Remarks to the Author):
I thank the authors for their careful and substantial revision of the manuscript. The revised version is significantly improved, and I find that the authors have addressed the major scientific concerns raised in the previous round of review in a satisfactory manner. In particular, the additional mechanistic experiments, the strengthened validation of the p38β–BiP axis, and the expanded in vitro and in vivo analyses have considerably reinforced the study and improved the overall rigor of the work.
In my view, the manuscript is now acceptable in principle. I have only one remaining point that I would ask the authors to address before final acceptance.
Because the proteomics/interactome dataset is used to support a central mechanistic conclusion, namely the prioritization of BiP as a key p38β interactor, I think this part of the manuscript should be presented in a more statistically rigorous manner. Specifically, I would request that the authors provide a proper volcano plot for the TAP-MS/interactome experiment, based on replicate-level quantitative protein intensity values and accompanied by the appropriate statistical analysis. This would allow the reader to directly evaluate both enrichment and statistical significance relative to the control condition and would substantially strengthen the presentation of the proteomics evidence.
Aside from this point, I consider the revision satisfactory and believe that the manuscript is suitable for publication after this minor final improvement.

Reply: We sincerely thank the reviewer for the positive evaluation and the constructive suggestion.

We agree that a volcano plot based on replicate-level quantitative protein intensity values, accompanied by appropriate statistical analysis, would represent the ideal presentation for a fully quantitative interactome dataset. However, we would like to clarify that the TAP-MS experiment was performed on replicates of whole-cell lysates from HEK293T cells expressing tagged p38β, with the goal to identify its potential interacting proteins. Specific binders were distinguished from non-specific interacting proteins on the beads by cross-comparison against the list of common contaminant proteins derived from our previous studies (Chan et al., 2018; Che et al., 2021). As a result, the data presented in Figure 3A and Supplementary Table S4 already exclude non-specific binding proteins, and the available quantitative information does not permit the generation of a statistically meaningful volcano plot.

We have now incorporated mean protein intensity values into the plot (revised Figure 3A) and have included replicate-level intensity values in Supplementary Table S4. Additionally, we have clarified in the Method section (lines 666-669, page 29) the exclusion of common contaminant proteins, and in the main text (lines 228–240, page 10) the rationale for selecting BiP as a

potential downstream target of p38β. The revised text now reads:

"To elucidate the mechanism by which elevated p38β drives stemness and chemoresistance in HCC, we performed tandem affinity purification coupled with mass spectrometry (TAP-MS) on whole-cell lysates from HEK293T cells expressing tagged p38β, aiming to identify its potential interacting proteins. This proteomic analysis identified multiple reported p38 targets and interacting proteins, including MAPKAPK2/3, RPS6KA4/5, MKNK1/2, and DUSP6/9 (Figure 3A; Supplementary Table S4). Among the putative p38β-interacting proteins, HSPA5 (BiP), which is an ER-resident chaperone, was prioritized as one of the previously unreported binding proteins of p38β, exhibiting comparable intensity and peptide numbers to those of established p38β interactors (Figure 3A). BiP is a master regulator of the UPR and has been implicated in the maintenance of cancer stem-like traits and chemoresistance across multiple tumor types 11, 31. Given the documented oncogenic functions of BiP and the availability of a specific inhibitor HA15, we proposed that BiP may represent a potential downstream target of p38β with therapeutic translation potential."

Chan LH, Zhou L, Ng KY, Wong TL, Lee TK, Sharma R, Loong JH, Ching YP, Yuan YF, Xie D, Lo CM, Man K, Artegiani B, Clevers H, Yan HH, Leung SY, Richard S, Guan XY, Huen MSY, Ma S. PRMT6 Regulates RAS/RAF Binding and MEK/ERK-Mediated Cancer Stemness Activities in Hepatocellular Carcinoma through CRAF Methylation. Cell Rep. 2018;25(3):690-701.e8.

Che N, Ng KY, Wong TL, Tong M, Kau PW, Chan LH, Lee TK, Huen MS, Yun JP, Ma S. PRMT6 deficiency induces autophagy in hostile microenvironments of hepatocellular carcinoma tumors by regulating BAG5-associated HSC70 stability. Cancer Lett. 2021;501:247-262.

Reviewer #3 (Remarks to the Author):
The revised version of this manuscript addresses most of my main concerns. However, some points still need to be addressed. Specifically:
- I requested that the western blot results be supported by quantifications and statistical analyses performed using independent experiments, with graphs shown in the Supplementary Figures. The authors replied: "We have now included quantitative analyses for all Western blot data, with densitometric intensities normalized to their respective loading controls". Unless I am mistaken, I am unable to find these quantifications and statistical analyses in the Supplementary Figures of the revised manuscript.

Reply: We thank the reviewer for pointing this out and apologize for the lack of clarity in our previous presentation. In the previous revised version, Western blot densitometric quantifications were performed and indicated below the corresponding blot bands. In the current revision, we have further presented these analyses as bar graphs in the Supplementary Figures, as requested.

Specifically, the quantifications for Figure 5B are shown in Supplementary Figure 8A-F; those for Supplementary Figure 8G are shown in Supplementary Figure 8H-M; those for Figure 5E are shown in Supplementary Figure 9A-F; those for Supplementary Figure 9H are shown in Supplementary Figure 9I-N; and those for Figure 5H are shown in Supplementary Figure 9O-

T.

- The authors now clarify the use of their previously published cDNA microarray profiling of chemoresistant hepatic tumourspheres generated by repeated cisplatin/doxorubicin selection. They specified that tumour-initiating cells were enriched from the HCC PLC/PRF/5 cell line by serial passages in serum-free spheroid culture supplemented with doxorubicin (2 mg/mL) and cisplatin (1 mg/mL), selecting for a self-renewing, chemoresistant population. In my opinion, this is a major concern for the study, as these doses correspond to about 3 mM, whereas these drugs are generally used at concentrations between 0.3 and 10 µM (thus 300 to 10,000 times higher than those generally used). For example, in a recent paper by Wang et al. (Cell Death Discovery 2024), the authors reported a dose response using doxorubicin within a range of 100 ng/mL up to 5 µM. The use of cells and data obtained at these extremely high doses, which are far from being physiologically and clinically relevant, might affect the pertinence of the outcomes and bias their relevance. The authors must clearly state this experimental context at the beginning of the results section where the data are mentioned, with a paragraph such as: "Briefly, tumour-initiating cells were enriched from the HCC cell line PLC/PRF/5 by serial passages in serum-free spheroid culture supplemented with doxorubicin (2 mg/mL) and cisplatin (1 mg/mL), selecting for a self-renewing, chemoresistant population. The chemoresistant tumourspheres and their differentiated counterparts were subjected to cDNA microarray profiling. We acknowledge that this transcriptomic profiling of chemoresistant hepatic tumourspheres was obtained using drug doses 300–10,000 times higher than those generally employed in experimental and clinical settings".

Reply: We thank the reviewer for this important comment. We have revised the beginning of the relevant Results section as follows:
"we leveraged our previously published transcriptomic profiling of chemoresistant hepatic tumorsphere (TIC-enriched) model (GSE53005) [27]. Briefly, tumor-initiating cells were enriched from the HCC cell line PLC/PRF/5 by serial passages in serum-free spheroid culture supplemented with doxorubicin (2 mg/mL) and cisplatin (1 mg/mL), selecting for a self-renewing, chemoresistant population. The chemoresistant tumorspheres and their differentiated counterparts were subjected to cDNA microarray profiling. We acknowledge that this transcriptomic profiling of chemoresistant hepatic tumorspheres was obtained using drug doses 300–10,000 times higher than those generally employed in experimental and clinical settings. This high-drug selection model was used here as a candidate-generating resource rather than as a direct short-term pharmacological response model. Using this dataset, we identified 37 differentially expressed kinase genes (log2 fold-change > 0.5) between chemoresistant hepatic tumorspheres and their differentiated counterparts. (lines 111-123, pages 6)

Reviewer #5 (Remarks to the Author):
The revised manuscript has been substantially improved. The authors have addressed many of the previous concerns, particularly regarding kinase activity validation, mechanistic linkage between p38β and BiP, and expansion of UPR-related readouts. The additional biochemical and in vivo data have strengthened the central conclusions, and overall the study is now more rigorous and convincing.

However, one important issue remains insufficiently resolved.

Major comment

Prioritization of p38β among the six candidate kinases remains insufficiently supported

The revised manuscript now provides a clearer description of the screening pipeline and explains how six overlapping kinase candidates were identified through integrated transcriptomic and dependency analyses. The authors further justify the selection of p38β based on its expression fold-change and the novelty of its link to BiP–UPR signaling.

While this rationale for prioritization is reasonable, it does not experimentally address the key concern: whether p38β is uniquely responsible for the observed phenotypes, or whether other shortlisted kinases (e.g., TYK2, FGFR4, PRKD2, SRPK3) may also contribute to chemoresistance and tumor-initiating capacity in HCC.

At present, no functional validation of these additional candidates has been provided. The response therefore represents a justification for focus, rather than a resolution of the concern. Given that several of these kinases are already implicated in cancer progression or drug resistance in other contexts, it remains difficult to exclude alternative or parallel drivers within the same system.

To strengthen the manuscript, the authors should consider:

Performing limited functional validation of one or two additional top candidates (e.g., knockdown ± cisplatin, sphere formation assays, or UPR-related readouts) to demonstrate that they do not recapitulate the p38β-dependent phenotypes;

or

Explicitly tempering the claims throughout the manuscript to indicate that p38β is a prioritized candidate from the shortlist, rather than the uniquely dominant regulator identified by the screen.

Addressing this point would significantly enhance the rigor of candidate selection and strengthen the overall conclusions.

Reply: We thank the reviewer for this important comment. We have revised the manuscript to temper the claim and clarify that p38β was prioritized from the shortlisted kinases for mechanistic validation, rather than the uniquely dominant regulator identified by the screen. (lines 129-130, pages 6)

Reviewer #6 (Remarks to the Author):

The authors have adequately addressed the concerns raised in the previous review of rewiever 4 and have provided well-argued responses supported by additional experimental data. In particular, the inclusion of Phos-tag SDS-PAGE, in vitro kinase assays, phospho-threonine detection on immunoprecipitated BiP, and additional controls addressing HSP70 family specificity substantially strengthens the evidence that BiP is phosphorylated by p38β at Thr648. The authors have also responded appropriately to the concerns regarding the reliance on cell-line-based systems by adding in vivo data using AAV8-mediated p38β expression in HCC PDX models. These experiments provide further support for the specificity and functional relevance of the p38β–BiP phosphorylation axis in tumor growth and stemness-related phenotypes.

In addition, the authors appear to have carefully addressed the comments raised by the other reviewers, and the revised manuscript is overall clearer, better supported, and more balanced.

The additional data and clarifications provided in the rebuttal adequately strengthen the main conclusions of the study.
Overall, I consider that the authors have responded appropriately and convincingly to the reviewers' concerns. In my opinion, the manuscript can now be accepted in its present form for publication in Nature Communications, without the need for further major revisions.

Reply: We sincerely thank the reviewers for their positive evaluation of our revised manuscript and for recommending acceptance for publication.

Reviewer #7 (Remarks to the Author):
I co-reviewed this manuscript with one of the reviewers who provided the listed reports. This is part of the Nature Communications initiative to facilitate training in peer review and to provide appropriate recognition for Early Career Researchers who co-review manuscripts.

Reply: We thank the co-reviewer for the time and contribution to the peer-review process.

Reviewer #2 (Remarks to the Author):
The authors have satisfactorily addressed the substantive points raised in Reviewer 2. Although the final request regarding the statistical presentation of the proteomics data was not implemented exactly in the form suggested, the authors provided a reasonable explanation for this limitation and improved the presentation accordingly. Overall, I consider the revision satisfactory.
Reply: We thank the reviewer for their careful evaluation and positive assessment of our revision.

Reviewer #3 (Remarks to the Author):
The authors have addressed the points raised adequately and provided quantification and statistical analysis for some, although not all, western blot analyses.
Reply: We thank the reviewer for their positive assessment of our revision.
