# Supplementary material for: p38β-mediated BiP phosphorylation drives stemness and chemoresistance by suppressing UPR activation in hepatocellular carcinoma
Source: Nat Commun. 2026 Aug 5;17:9415. doi: 10.1038/s41467-026-76073-7 (PMC13538570; doi:10.1038/s41467-026-76073-7)
Supplement: Supplementary file 5 [file 41467_2026_76073_MOESM3_ESM.zip › R6-Supplementary Data/Supplementary Data Descriptions.docx]

**Supplementary Data for**

**p38β-mediated BiP phosphorylation drives stemness and chemoresistance by suppressing UPR activation in hepatocellular carcinoma**

Liang Xu^1*^, Ianto Bosheng Huang^2,3*^, Minghe Zhang^1*^, Yunong Xie^1^, Bing Li^4^, Linglin Liu^1^, Long Hin Ng^1^, Yimiao He^1^, Yan Liu^1^, Tin-Lok Wong^2^, Xiaoyun Lu^5^, Terence Kin-Wah Lee^6^, Jing-Ping Yun^7^, Lei Jin^4^, Stephanie Ma^2,8,9^, Man Tong^1^^

^^^ Correspondence: Man Tong, Ph.D.

E-mail: carolmantong@cuhk.edu.hk

This data file includes:

**Supplementary Data 1.** Correlation of p38β expression with clinicopathological characteristics in a cohort of 136 HCC patients.

**Supplementary Data 2.** List of p38β-immunoprecipited proteins identified by LC–MS/MS.

**Supplementary Data 3.** BiP phosphorylation sites identified by LC–MS/MS.

**Supplementary Data 4.** Clinicopathological characteristics of the HCC PDX donor patients.

**Supplementary Data 5.** List of reagents and resources.

**Supplementary Data 6.** List of siRNAs.
